# Factors affecting asphalt concrete permanent deformation: Experimental dataset for uniaxial repeated load test

**DOI:** 10.1016/j.dib.2024.110224

**Published:** 2024-02-20

**Authors:** Amjad H. Albayati

**Affiliations:** Department of civil engineering, University of Baghdad, Baghdad 10071, Iraq

**Keywords:** Permanent deformation, Asphalt concrete, Uniaxial test, Traffic condition, Temperature

## Abstract

Permanent deformation in asphalt concrete pavements is pervasive distress [1], influenced by various factors such as environmental conditions, traffic loading, and mixture properties. A meticulous investigation into these factors has been conducted, yielding a robust dataset from uniaxial repeated load tests on 108 asphalt concrete samples. Each sample underwent systematic evaluation under varied test temperatures, loading conditions, and mixture properties, ensuring the data's comprehensiveness and reliability. The materials used, sourced locally, were selected to enhance the studyʼs relevance to pavement constructions in hot climate areas, considering different asphalt cement grades and contents to understand material variability effects on deformation. The detailed dataset created from the experimental program acts as a pivotal resource for refining predictive models and optimizing asphalt concrete mixtures and pavement design strategies, aimed at improving pavement performance and longevity under diverse operational and environmental conditions.

Specifications Table

**Table:** 

Subject area	Transportation engineering
Specific subject area	Asphalt concrete pavement
Data format	Raw
Type of data	Table
Data collection	The data obtained from testing 108 asphalt concrete samples in Uniaxial repeated load test to characterize permanent deformation in asphalt concrete. The factors considered are three levels of testing temperature (20 °C, 40 °C, 60 °C), and three level of uniaxial stress (10 psi, 20 psi, 30 psi) with two durations (0.1 s., 0.4 s.). The mix variables are (Two asphalt grades: 40–50, 60–70, and three asphalt contents: Opt.−0.6, Opt., Opt.+0.6).
Data source location	Pavement material lab in the Civil Engineering Department of the University of Baghdad
Data accessibility	Data is available at: Repository name: Mendeley Data Doi: 10.17632/sctmp96v7n.2 Direct URL to data: https://data.mendeley.com/datasets/sctmp96v7n/1

## 1 Value of the Data

• **Facilitates understanding:** The comprehensive dataset serves as an essential tool for elucidating the nuanced effects of critical factors like temperature, stress level, and asphalt composition on the susceptibility of asphalt concrete pavements to permanent deformation. Its detailed nature facilitates a level of understanding that is imperative for advancing pavement technology.
• **Enhancement of predictive models**: Utilizing this rich dataset, existing predictive models can be refined and enhanced, enabling more accurate predictions concerning asphalt concrete pavement performance under various conditions.
• **Optimization of material selection and mixture design:** Insights from this dataset can guide the selection of materials and the design of asphalt concrete mixtures, optimizing them for enhanced performance and longevity.
• **Informed pavement design strategies:** The data serves as a critical resource for developing pavement design strategies that are resilient to the diverse operational and environmental challenges encountered in hot climate areas.
• **Promotes further research:** This dataset acts as a foundation, encouraging further research and exploration into mitigating the challenges associated with permanent deformation in asphalt concrete pavements.

## 2 Data Description

The dataset presented in this study is a compilation of experimental results aimed at investigating the factors affecting the permanent deformation of asphalt concrete pavements. Comprehensively curated, the dataset comprises outcomes from uniaxial repeated load tests conducted on 108 asphalt concrete samples, with considerations of variable factors such as temperature, stress level, asphalt grade, and asphalt content. A meticulous examination under varying conditions, temperatures (20 °C, 40 °C, 60 °C), stress levels (10 psi, 20 psi, 30 psi), and asphalt grades and contents, has been performed to ensure a broad spectrum of data.

In a structured format, the data encapsulates detailed insights into the behavioral tendencies of asphalt concrete under specific operational scenarios, making it a pivotal resource for enhancing the empirical understanding and predictive modeling of pavement performance. This dataset is instrumental for researchers, pavement engineers, and decision-makers aiming to derive meaningful interpretations that can foster improved pavement design, material selection, and overall asphalt concrete pavement longevity in hot climate regions.

## 3 Material Characterization

The materials used in the experimental work, namely asphalt cement, aggregate, and fillers were characterized using a routine type of tests and results were compared with the Iraqi State Corporation for Roads and Bridges specifications, SCRB [1], [2].

### 3.1 Asphalt cement

To determine the effect of asphalt cement type upon deformation, two asphalt cement grades were considered: AC 40–50 and 60–70. Both types are obtained from the Dora refinery, southwest of Baghdad. The asphalt cement properties are shown in Table 1 below.

**Table 1:** Properties of asphalt cement.

Property	ASTM designation	Penetration grade 40–50		Penetration grade 60–70	
		Test results	SCRB specification [2]	Test results	SCRB specification [2]
1-Penetration At 25C,100 gm,5 s. (0.1 mm)	D-5	44	40–50	65	60–70
2- Softening Point. (°C)	D-36	51	……	44	…..
3-Ductility at 25 C, 5 cm/min,(cm)	D-113	>100	>100	….	>100
4-Flash Point, (°C)	D-92	294	Min.232	290	Min.232
5-Specific Gravity	D-70	1.042	……	1.027	……
6- Residue from thin film oven test - Retained penetration,% of original - Ductility at 25 C, 5 cm/min,(cm)	D-1754 D-5 D-113	59.3 70	55^+^ 25^+^	62.1 100^+^	52^+^ 50^+^

### 3.2 Aggregate

**Fig. 1:**
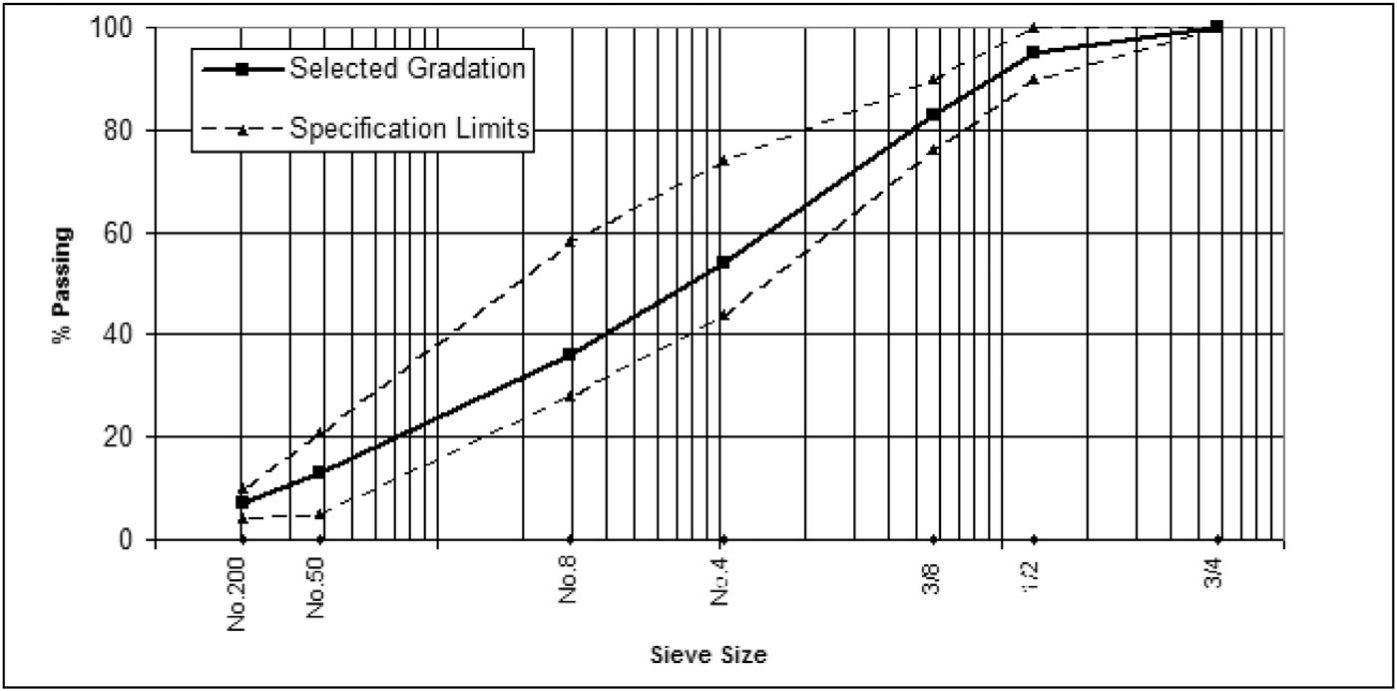
Aggregate gradation chart.

The aggregate used in the experimental work was crushed quartz obtained from the Amanat Baghdad asphalt concrete mix plant located in Taji, north of Baghdad. This aggregate is widely used in Baghdad city for asphaltic mixes. The coarse and fine aggregates used in this work were sieved and recombined in the proper proportions to meet the wearing course gradation as required by SCRB specification [2]. The gradation curve for the aggregate is shown in Fig. 1.

Routine tests were performed on the aggregate to evaluate their physical properties. The results together with the specification limits as set by the SCRB are summarized in Table 2. Test results show that the chosen aggregate met the SCRB specifications [2].

**Table 2:** Physical properties of aggregates.

Property	ASTM designation	Test results	SCRB specification [2]
Coarse aggregate 1. Bulk specific gravity 2. Apparent specific gravity 3. Water absorption,% 4. Percent wear by Los Angeles abrasion,% 5. Soundness loss by sodium sulfate solution,% 6. Fractured pieces,%	C-127 C-131 C-88	2.631 2.691 0.458 19.3 3.9 94	…… …… …… 30 Max 10 Max 95 Min
Fine aggregate 1. Bulk specific gravity 2. Apparent specific gravity 3. Water absorption,% 4. Sand equivalent,%	C-127 D-2419	2.661 2.695 0.901 52	…… …… …… 45 Min.

### 3.3 Mineral filler

The filler is a nonplastic material passing sieve No.200 (0.075 mm). The filler used in the experimental work is limestone dust obtained from the Ammanat Baghdad asphalt concrete mix plant; its source is the lime factory in Karbala governorate (south of Baghdad). The physical properties of the used filler are presented in Table 3 below:

**Table 3:** Physical properties of mineral filler.

Property	Test results
Specific gravity	2.787
Passing sieve No.200 (0.075 mm)	96

## 4 Preliminary Test: Marshall Mix Design

To fulfill the requirements of the factorial experiment design, tests were conducted at optimum asphalt content and asphalt contents 0.6 percent above, and 0.6 percent below optimum. To determine the optimum asphalt content, a complete mix design was conducted using the Marshall method as outlined in AI's manual series No.2 [3]. Based upon this method, the optimum asphalt content is determined by averaging the three values shown below:

• Asphalt content at maximum unit weight
• Asphalt content at maximum stability
• Asphalt content at 4% air voids

**Fig. 2:**
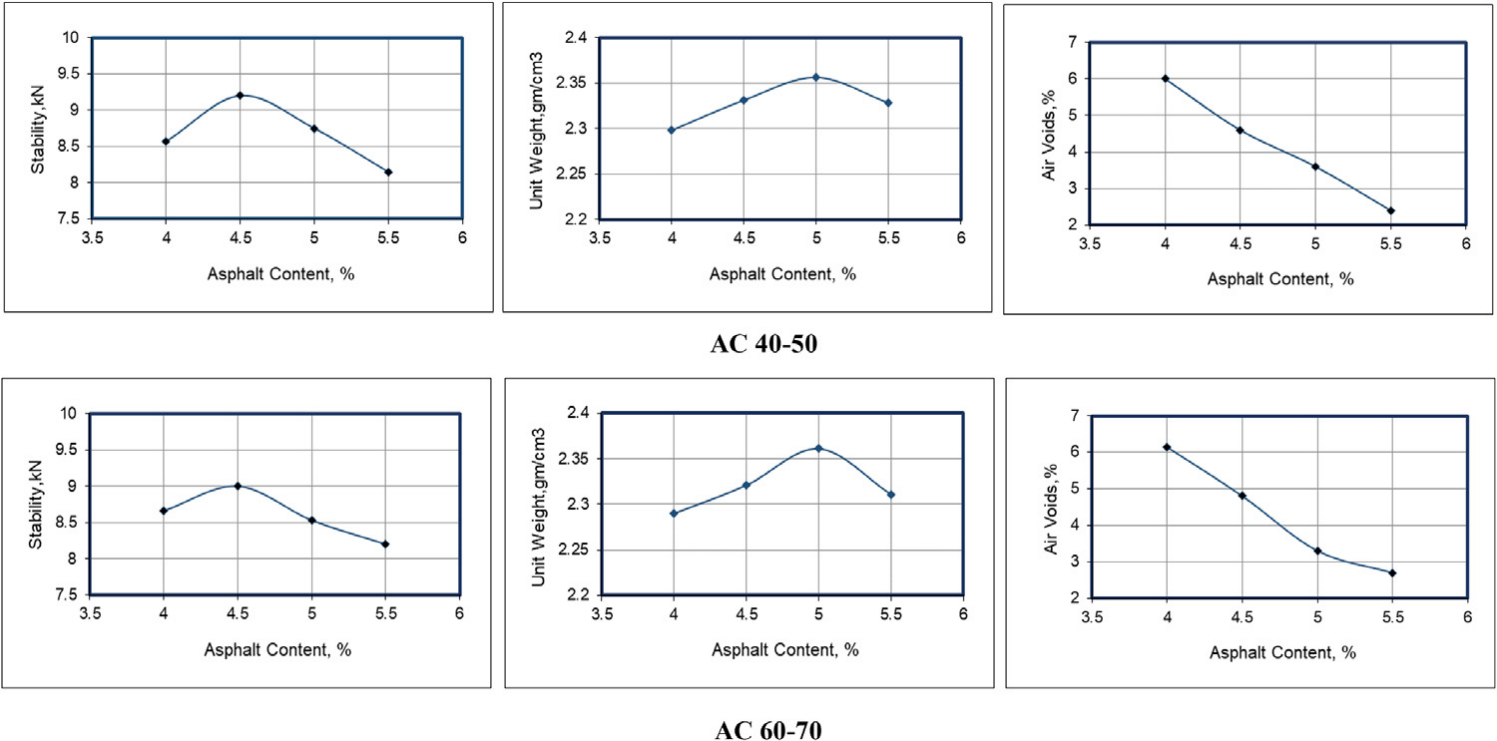
Marshall plots for mixtures.

Fig. 2 shows the plots of the Marshall data for each type of asphalt cement with an optimum asphalt content of 4.6 percent for both grades of asphalt. Table 4 presents the properties of the mixture at optimum asphalt contents for the design criteria of 75 Marshall blows. The data shown in Table 4 indicate that both mixtures (i.e., containing AC 40–50 and AC 60–70) meet the SCRB specifications [2].

**Table 4:** Mix properties at optimum asphalt content and specification requirements.

Marshall property	Mix type		SCRB specifications [2]
	AC 40–50	AC 60–70	
Stability, kN	9.25	9.02	8 min.
Flow, mm	3.50	3.75	2–4
Percent air voids	4.20	4.35	3–5
Percent VMA	15.46	16.00	14 min.

## 5 Preparation of Specimens

The cylindrical specimens used in this study have dimensions of 101.6 mm (4 in.) in diameter and 203.2 mm (8 in.) in height. The various aggregate fractions, as received from the mixing plant, were grouped according to retention on specific sieves (½, 3/8, No. 4, No. 8, No. 50, No. 200, and pan) using dry sieve analysis. Aggregates retained on the pan were discarded and replaced with mineral filler (limestone dust).

Aligning with the gradation requirements depicted in Fig. 1, the aggregates were batched at 3800 g in a mixing bowl and heated to 150 °C in a temperature-controlled oven. The asphalt cement was also heated to a temperature ranging between 140 and 145 °C, and the precise amount of asphalt cement was added to the mixing bowl on an electrical balance. The contents were mixed thoroughly on a hot plate for two minutes. To ensure a uniform compaction temperature, the bowl and its contents were transferred to an oven and maintained at 140 °C for 10 min.

Simultaneously, the compaction mold (4 inches in diameter and 10 inches in height), preheated to 100 °C, was prepared. A 4-inch paper disk was placed to cover the mold's base plate, and the interior edge of the mold was lubricated to facilitate later specimen extraction. Using a funnel, approximately half of the mixture was added to the mold, followed by vigorous spading with a heated spatula or trowel, 15 times around the perimeter and 10 times over the interior. This procedure was repeated for the remaining mixture.

The specimen underwent compaction using the double plunger method, where a 65,000 lb (29,491 kg) load was applied by a hydraulic compression machine. The load was applied to each end of the specimen for one minute. Lastly, the specimen was gently moved to a smooth, flat surface, allowed to cool overnight at room temperature, and then removed from the mold using a hydraulic extractor. Each specimen was labeled and stored in a bag until ready for testing.

## 6 Uniaxial Repeated Loading Test

**Fig. 3:**
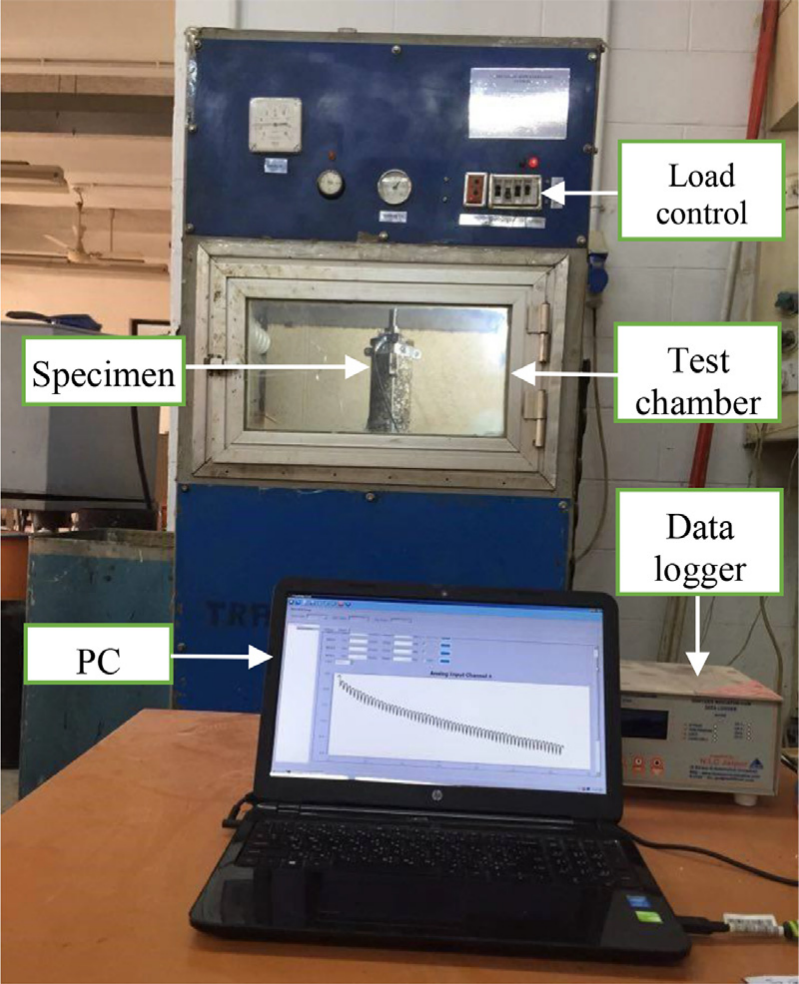
Photograph for the PRLS.

The axial repeated load tests were executed utilizing the Pneumatic Repeated Load System (PRLS), as shown in Fig. 3. Repetitive compressive stress was applied on the specimens, and the resulting axial permanent deformations were recorded across various loading repetitions. Each compressive applied was applied as a rectangular wave at a steady frequency of 60 cycles per minute, using two distinct loading sequences: one with a 0.1 s. load duration followed by a 0.9 s. rest period, and another with a 0.4 s. load duration followed by a 0.6 s. rest period. The experiments utilized three stress levels (10, 20, and 30 psi) and were conducted at three different temperatures (20, 40, and 60 °C).

The permanent strain (εp) is calculated by applying the following equation:

(1) $$\varepsilon_{p}=\frac{p_{d}\times 10^{6}}{h}$$

where

εp = axial permanent microstrain

pd = axial permanent deformation

*h* = specimen height

## 7 Factors Affecting Permanent Deformation

**Fig. 4:**
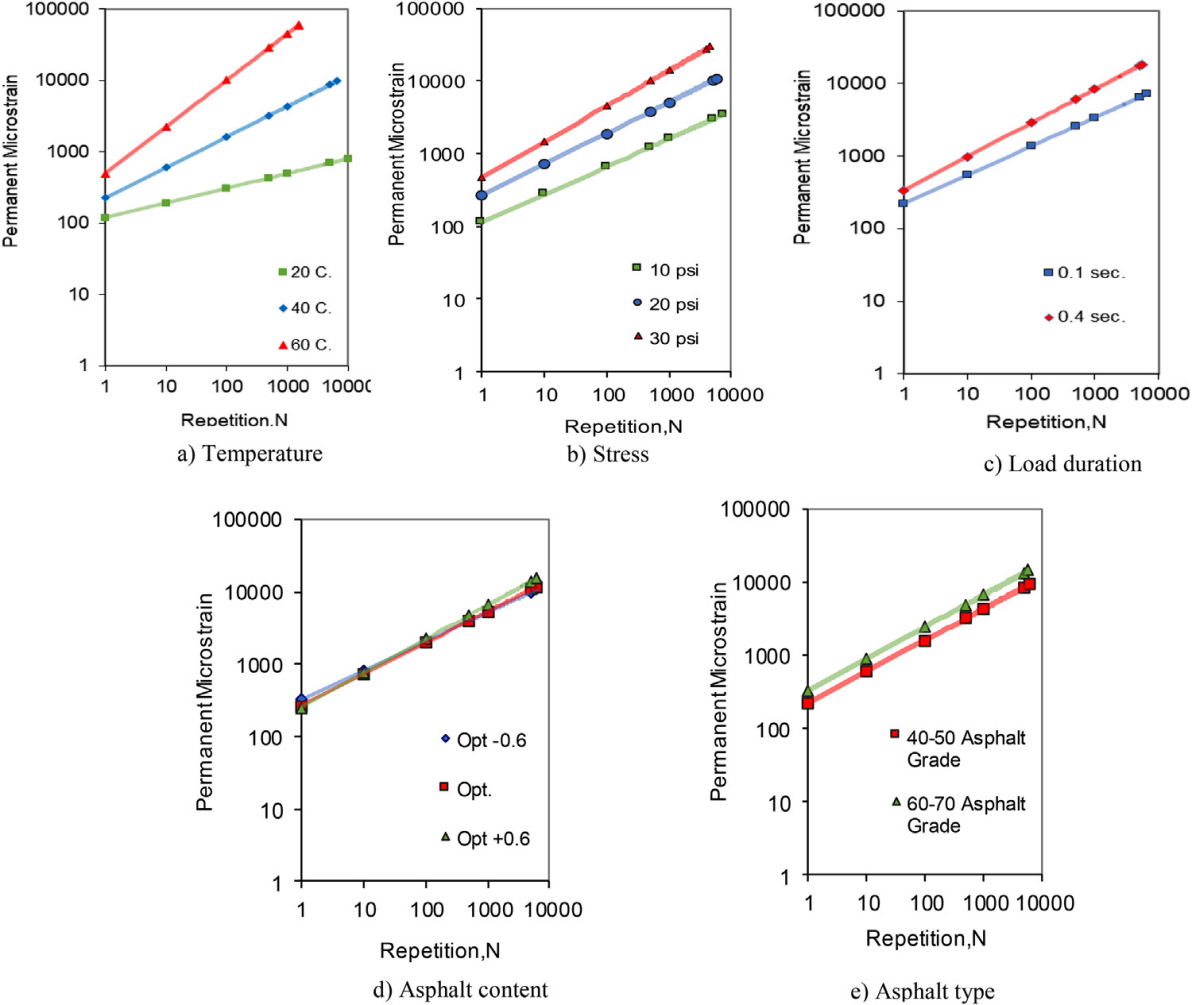
Effect of variables on permanent deformation.

To ascertain the influence of investigated factors on permanent deformation, the dataset was meticulously sorted by key variables, including temperature, stress magnitude, load duration, asphalt type, and content. For example, data were categorized by test temperature, and the mean permanent deformation at each temperature was computed to elucidate the relationship between permanent deformation (εp) and the number of load repetitions (N), which followed the trend line equation εp = aN^b, where ‘a’ and ‘b’ are fitting coefficients. Graphical representations depicting the effects of these variables on permanent deformation are presented in Fig. 4.

Fig. 4a clearly demonstrates that an increase in temperature from 20 °C to 60 °C results in a substantial increase in permanent deformation, with a staggering rise of approximately 2465%, highlighting a very high sensitivity to temperature variations. As shown in Fig. 4b, stress level is a pivotal factor; deformation at 30 psi is over four times (an increase of more than 304%) the deformation at 10 psi, indicating a pronounced correlation between stress levels and deformation. Fig. 4c reveals that extending load duration from 0.1 to 0.4 s correlates with a 65% increase in permanent deformation, implying a moderate dependency on the duration of applied load. According to Fig. 4d, changes in asphalt content significantly affect deformation, which increases by about 26% when the content is adjusted from Opt. −0.6% to Opt. +0.6%, marking a noticeable but comparatively smaller impact. Moreover, as Fig. 4e indicates, different asphalt grades have differential effects on deformation; the 60–70 grade exhibits around 35% more deformation than the 40–50 grade, indicating a considerable effect, though it is less pronounced than other variables.

In summary, this analysis suggests that plastic strain (εp) is highly sensitive to temperature and stress, with a moderate influence from load duration. While asphalt content and grade have a less marked effect, they nonetheless play a role in the plastic response of asphalt concrete. These factors should be prioritized accordingly—temperature, stress, load duration, followed by asphalt properties—when assessing and designing asphalt concrete pavements for optimal performance as related to permanent deformation.

## 8 Data Presentation and Availability

The test results of permanent deformation are presented in the form of permanent strain (microstrain) as a function of loading repetition (N) for mixes with different penetration grades of asphalt cement (P), percent absorbed asphalt, by weight of aggregate (B), percent effective asphalt, by volume of mix (E), percent air voids (A), percent voids in the mineral aggregate, (M), voids filed with asphalt (F), percent asphalt cement content (C). The tests were carried out under different testing temperature tests in degree centigrade (T), stress level, psi (S), and applied stress duration, s. (D). The data were used in [4,5] and [6] to explore and model the temperature effect on asphalt concrete permanent deformation. All the data for test results could be accessed via the following URL link https://data.mendeley.com/datasets/sctmp96v7n/1

## Limitations

The dataset primarily focuses on distinct asphalt concrete material properties and testing conditions, which narrows its applicability to material with similar characteristics and test conditions.

## Ethics Statement

The author has followed the ethical guidelines for publication in Data in Brief. It is confirmed that the present work has not engaged in experiments involving human subjects or animals, nor has it utilized data gathered from social media platforms.

## CRediT Author Statement

Amjad H. Albayati is Solely responsible for all aspects of the study including conceptualization, methodology, data curation, writing, and revision of the manuscript.

## Data Availability

Uniaxial Repeated Load Test Data of Asphalt concrete (Original data) (Mendeley Data)

## Appendix Supplementary materials

Image, application 1
