# Supplementary material for: Factors affecting asphalt concrete permanent deformation: Experimental dataset for uniaxial repeated load test
Source: Data Brief. 2024 Feb 20;53:110224. doi: 10.1016/j.dib.2024.110224 (PMC10907153; doi:10.1016/j.dib.2024.110224)
Supplement: Supplementary file 1 [file mmc1.docx]

**Appendix A**

**Uniaxial Repeated Load Test Data**

Legend

N = load repetition

T = test temperature in degree centigrade (^◦^C)

S = stress level (psi)

D = applied stress duration (sec.)

P = penetration grade

B = percent absorbed asphalt (by weight of aggregate)

E = percent effective asphalt (by volume of mix)

A = percent air voids

M = percent voids in the mineral aggregate

F = voids filed with asphalt

G = asphalt cement grade

C = percent asphalt cement content

E_p_ = ε_p_, permanent microstrain at load repetition N

| **N** | **E_p_** | **T** | **S** | **P** | **C** | **D** | **A** | **M** | **F** | **B** | **E** |
| --- | --- | --- | --- | --- | --- | --- | --- | --- | --- | --- | --- |
| 1 | 30 | 20 | 10 | 44 | 4 | 0.1 | 6.36 | 16.3 | 60.95 | 0.061 | 8.725 |
| 2 | 32 | 20 | 10 | 44 | 4 | 0.1 | 6.36 | 16.3 | 60.95 | 0.061 | 8.725 |
| 10 | 35 | 20 | 10 | 44 | 4 | 0.1 | 6.36 | 16.3 | 60.95 | 0.061 | 8.725 |
| 100 | 43 | 20 | 10 | 44 | 4 | 0.1 | 6.36 | 16.3 | 60.95 | 0.061 | 8.725 |
| 500 | 50 | 20 | 10 | 44 | 4 | 0.1 | 6.36 | 16.3 | 60.95 | 0.061 | 8.725 |
| 1000 | 51 | 20 | 10 | 44 | 4 | 0.1 | 6.36 | 16.3 | 60.95 | 0.061 | 8.725 |
| 2000 | 55 | 20 | 10 | 44 | 4 | 0.1 | 6.36 | 16.3 | 60.95 | 0.061 | 8.725 |
| 3000 | 55 | 20 | 10 | 44 | 4 | 0.1 | 6.36 | 16.3 | 60.95 | 0.061 | 8.725 |
| 4000 | 57 | 20 | 10 | 44 | 4 | 0.1 | 6.36 | 16.3 | 60.95 | 0.061 | 8.725 |
| 5000 | 58 | 20 | 10 | 44 | 4 | 0.1 | 6.36 | 16.3 | 60.95 | 0.061 | 8.725 |
| 6000 | 59 | 20 | 10 | 44 | 4 | 0.1 | 6.36 | 16.3 | 60.95 | 0.061 | 8.725 |
| 7000 | 60 | 20 | 10 | 44 | 4 | 0.1 | 6.36 | 16.3 | 60.95 | 0.061 | 8.725 |
| 8000 | 60 | 20 | 10 | 44 | 4 | 0.1 | 6.36 | 16.3 | 60.95 | 0.061 | 8.725 |
| 9000 | 61 | 20 | 10 | 44 | 4 | 0.1 | 6.36 | 16.3 | 60.95 | 0.061 | 8.725 |
| 10000 | 62 | 20 | 10 | 44 | 4 | 0.1 | 6.36 | 16.3 | 60.95 | 0.061 | 8.725 |
| 1 | 10 | 20 | 10 | 44 | 4.6 | 0.1 | 4.06 | 15.37 | 73.57 | 0.061 | 10.23 |
| 2 | 10 | 20 | 10 | 44 | 4.6 | 0.1 | 4.06 | 15.37 | 73.57 | 0.061 | 10.23 |
| 10 | 13 | 20 | 10 | 44 | 4.6 | 0.1 | 4.06 | 15.37 | 73.57 | 0.061 | 10.23 |
| 100 | 15 | 20 | 10 | 44 | 4.6 | 0.1 | 4.06 | 15.37 | 73.57 | 0.061 | 10.23 |
| 500 | 18 | 20 | 10 | 44 | 4.6 | 0.1 | 4.06 | 15.37 | 73.57 | 0.061 | 10.23 |
| 1000 | 20 | 20 | 10 | 44 | 4.6 | 0.1 | 4.06 | 15.37 | 73.57 | 0.061 | 10.23 |
| 2000 | 21 | 20 | 10 | 44 | 4.6 | 0.1 | 4.06 | 15.37 | 73.57 | 0.061 | 10.23 |
| 3000 | 22 | 20 | 10 | 44 | 4.6 | 0.1 | 4.06 | 15.37 | 73.57 | 0.061 | 10.23 |
| 4000 | 23 | 20 | 10 | 44 | 4.6 | 0.1 | 4.06 | 15.37 | 73.57 | 0.061 | 10.23 |
| 5000 | 23 | 20 | 10 | 44 | 4.6 | 0.1 | 4.06 | 15.37 | 73.57 | 0.061 | 10.23 |
| 6000 | 23 | 20 | 10 | 44 | 4.6 | 0.1 | 4.06 | 15.37 | 73.57 | 0.061 | 10.23 |
| 7000 | 24 | 20 | 10 | 44 | 4.6 | 0.1 | 4.06 | 15.37 | 73.57 | 0.061 | 10.23 |
| 8000 | 24 | 20 | 10 | 44 | 4.6 | 0.1 | 4.06 | 15.37 | 73.57 | 0.061 | 10.23 |
| 9000 | 24 | 20 | 10 | 44 | 4.6 | 0.1 | 4.06 | 15.37 | 73.57 | 0.061 | 10.23 |
| 10000 | 25 | 20 | 10 | 44 | 4.6 | 0.1 | 4.06 | 15.37 | 73.57 | 0.061 | 10.23 |
| 1 | 25 | 20 | 10 | 44 | 5.2 | 0.1 | 2.81 | 15.08 | 81.38 | 0.061 | 11.695 |
| 2 | 27 | 20 | 10 | 44 | 5.2 | 0.1 | 2.81 | 15.08 | 81.38 | 0.061 | 11.695 |
| 10 | 32 | 20 | 10 | 44 | 5.2 | 0.1 | 2.81 | 15.08 | 81.38 | 0.061 | 11.695 |
| 100 | 42 | 20 | 10 | 44 | 5.2 | 0.1 | 2.81 | 15.08 | 81.38 | 0.061 | 11.695 |
| 500 | 50 | 20 | 10 | 44 | 5.2 | 0.1 | 2.81 | 15.08 | 81.38 | 0.061 | 11.695 |
| 1000 | 55 | 20 | 10 | 44 | 5.2 | 0.1 | 2.81 | 15.08 | 81.38 | 0.061 | 11.695 |
| 2000 | 59 | 20 | 10 | 44 | 5.2 | 0.1 | 2.81 | 15.08 | 81.38 | 0.061 | 11.695 |
| 3000 | 62 | 20 | 10 | 44 | 5.2 | 0.1 | 2.81 | 15.08 | 81.38 | 0.061 | 11.695 |
| 4000 | 64 | 20 | 10 | 44 | 5.2 | 0.1 | 2.81 | 15.08 | 81.38 | 0.061 | 11.695 |
| 5000 | 65 | 20 | 10 | 44 | 5.2 | 0.1 | 2.81 | 15.08 | 81.38 | 0.061 | 11.695 |
| 6000 | 67 | 20 | 10 | 44 | 5.2 | 0.1 | 2.81 | 15.08 | 81.38 | 0.061 | 11.695 |
| 7000 | 68 | 20 | 10 | 44 | 5.2 | 0.1 | 2.81 | 15.08 | 81.38 | 0.061 | 11.695 |
| 8000 | 70 | 20 | 10 | 44 | 5.2 | 0.1 | 2.81 | 15.08 | 81.38 | 0.061 | 11.695 |
| 9000 | 70 | 20 | 10 | 44 | 5.2 | 0.1 | 2.81 | 15.08 | 81.38 | 0.061 | 11.695 |
| 10000 | 71 | 20 | 10 | 44 | 5.2 | 0.1 | 2.81 | 15.08 | 81.38 | 0.061 | 11.695 |
| 1 | 75 | 20 | 20 | 44 | 4 | 0.1 | 6.34 | 16.28 | 61.06 | 0.061 | 8.727 |
| 2 | 80 | 20 | 20 | 44 | 4 | 0.1 | 6.34 | 16.28 | 61.06 | 0.061 | 8.727 |
| 10 | 100 | 20 | 20 | 44 | 4 | 0.1 | 6.34 | 16.28 | 61.06 | 0.061 | 8.727 |
| 100 | 140 | 20 | 20 | 44 | 4 | 0.1 | 6.34 | 16.28 | 61.06 | 0.061 | 8.727 |
| 500 | 170 | 20 | 20 | 44 | 4 | 0.1 | 6.34 | 16.28 | 61.06 | 0.061 | 8.727 |
| 1000 | 190 | 20 | 20 | 44 | 4 | 0.1 | 6.34 | 16.28 | 61.06 | 0.061 | 8.727 |
| **N** | **E_p_** | **T** | **S** | **P** | **C** | **D** | **A** | **M** | **F** | **B** | **E** |
| 2000 | 210 | 20 | 20 | 44 | 4 | 0.1 | 6.34 | 16.28 | 61.06 | 0.061 | 8.727 |
| 3000 | 220 | 20 | 20 | 44 | 4 | 0.1 | 6.34 | 16.28 | 61.06 | 0.061 | 8.727 |
| 4000 | 230 | 20 | 20 | 44 | 4 | 0.1 | 6.34 | 16.28 | 61.06 | 0.061 | 8.727 |
| 5000 | 235 | 20 | 20 | 44 | 4 | 0.1 | 6.34 | 16.28 | 61.06 | 0.061 | 8.727 |
| 6000 | 240 | 20 | 20 | 44 | 4 | 0.1 | 6.34 | 16.28 | 61.06 | 0.061 | 8.727 |
| 7000 | 245 | 20 | 20 | 44 | 4 | 0.1 | 6.34 | 16.28 | 61.06 | 0.061 | 8.727 |
| 8000 | 250 | 20 | 20 | 44 | 4 | 0.1 | 6.34 | 16.28 | 61.06 | 0.061 | 8.727 |
| 9000 | 255 | 20 | 20 | 44 | 4 | 0.1 | 6.34 | 16.28 | 61.06 | 0.061 | 8.727 |
| 10000 | 260 | 20 | 20 | 44 | 4 | 0.1 | 6.34 | 16.28 | 61.06 | 0.061 | 8.727 |
| 1 | 35 | 20 | 20 | 44 | 4.6 | 0.1 | 3.93 | 15.25 | 74.23 | 0.061 | 10.244 |
| 2 | 40 | 20 | 20 | 44 | 4.6 | 0.1 | 3.93 | 15.25 | 74.23 | 0.061 | 10.244 |
| 10 | 55 | 20 | 20 | 44 | 4.6 | 0.1 | 3.93 | 15.25 | 74.23 | 0.061 | 10.244 |
| 100 | 80 | 20 | 20 | 44 | 4.6 | 0.1 | 3.93 | 15.25 | 74.23 | 0.061 | 10.244 |
| 500 | 110 | 20 | 20 | 44 | 4.6 | 0.1 | 3.93 | 15.25 | 74.23 | 0.061 | 10.244 |
| 1000 | 120 | 20 | 20 | 44 | 4.6 | 0.1 | 3.93 | 15.25 | 74.23 | 0.061 | 10.244 |
| 2000 | 140 | 20 | 20 | 44 | 4.6 | 0.1 | 3.93 | 15.25 | 74.23 | 0.061 | 10.244 |
| 3000 | 150 | 20 | 20 | 44 | 4.6 | 0.1 | 3.93 | 15.25 | 74.23 | 0.061 | 10.244 |
| 4000 | 155 | 20 | 20 | 44 | 4.6 | 0.1 | 3.93 | 15.25 | 74.23 | 0.061 | 10.244 |
| 5000 | 165 | 20 | 20 | 44 | 4.6 | 0.1 | 3.93 | 15.25 | 74.23 | 0.061 | 10.244 |
| 6000 | 170 | 20 | 20 | 44 | 4.6 | 0.1 | 3.93 | 15.25 | 74.23 | 0.061 | 10.244 |
| 7000 | 175 | 20 | 20 | 44 | 4.6 | 0.1 | 3.93 | 15.25 | 74.23 | 0.061 | 10.244 |
| 8000 | 180 | 20 | 20 | 44 | 4.6 | 0.1 | 3.93 | 15.25 | 74.23 | 0.061 | 10.244 |
| 9000 | 180 | 20 | 20 | 44 | 4.6 | 0.1 | 3.93 | 15.25 | 74.23 | 0.061 | 10.244 |
| 10000 | 185 | 20 | 20 | 44 | 4.6 | 0.1 | 3.93 | 15.25 | 74.23 | 0.061 | 10.244 |
| 1 | 15 | 20 | 20 | 44 | 5.2 | 0.1 | 2.84 | 15.1 | 81.22 | 0.061 | 11.691 |
| 2 | 18 | 20 | 20 | 44 | 5.2 | 0.1 | 2.84 | 15.1 | 81.22 | 0.061 | 11.691 |
| 10 | 25 | 20 | 20 | 44 | 5.2 | 0.1 | 2.84 | 15.1 | 81.22 | 0.061 | 11.691 |
| 100 | 40 | 20 | 20 | 44 | 5.2 | 0.1 | 2.84 | 15.1 | 81.22 | 0.061 | 11.691 |
| 500 | 60 | 20 | 20 | 44 | 5.2 | 0.1 | 2.84 | 15.1 | 81.22 | 0.061 | 11.691 |
| 1000 | 75 | 20 | 20 | 44 | 5.2 | 0.1 | 2.84 | 15.1 | 81.22 | 0.061 | 11.691 |
| 2000 | 85 | 20 | 20 | 44 | 5.2 | 0.1 | 2.84 | 15.1 | 81.22 | 0.061 | 11.691 |
| 3000 | 95 | 20 | 20 | 44 | 5.2 | 0.1 | 2.84 | 15.1 | 81.22 | 0.061 | 11.691 |
| 4000 | 105 | 20 | 20 | 44 | 5.2 | 0.1 | 2.84 | 15.1 | 81.22 | 0.061 | 11.691 |
| 5000 | 105 | 20 | 20 | 44 | 5.2 | 0.1 | 2.84 | 15.1 | 81.22 | 0.061 | 11.691 |
| 6000 | 110 | 20 | 20 | 44 | 5.2 | 0.1 | 2.84 | 15.1 | 81.22 | 0.061 | 11.691 |
| 7000 | 120 | 20 | 20 | 44 | 5.2 | 0.1 | 2.84 | 15.1 | 81.22 | 0.061 | 11.691 |
| 8000 | 120 | 20 | 20 | 44 | 5.2 | 0.1 | 2.84 | 15.1 | 81.22 | 0.061 | 11.691 |
| 9000 | 125 | 20 | 20 | 44 | 5.2 | 0.1 | 2.84 | 15.1 | 81.22 | 0.061 | 11.691 |
| 10000 | 130 | 20 | 20 | 44 | 5.2 | 0.1 | 2.84 | 15.1 | 81.22 | 0.061 | 11.691 |
| 1 | 165 | 20 | 30 | 44 | 4 | 0.1 | 6.36 | 16.3 | 60.95 | 0.061 | 8.725 |
| 2 | 185 | 20 | 30 | 44 | 4 | 0.1 | 6.36 | 16.3 | 60.95 | 0.061 | 8.725 |
| 10 | 245 | 20 | 30 | 44 | 4 | 0.1 | 6.36 | 16.3 | 60.95 | 0.061 | 8.725 |
| 100 | 360 | 20 | 30 | 44 | 4 | 0.1 | 6.36 | 16.3 | 60.95 | 0.061 | 8.725 |
| 500 | 475 | 20 | 30 | 44 | 4 | 0.1 | 6.36 | 16.3 | 60.95 | 0.061 | 8.725 |
| 1000 | 535 | 20 | 30 | 44 | 4 | 0.1 | 6.36 | 16.3 | 60.95 | 0.061 | 8.725 |
| 2000 | 600 | 20 | 30 | 44 | 4 | 0.1 | 6.36 | 16.3 | 60.95 | 0.061 | 8.725 |
| 3000 | 645 | 20 | 30 | 44 | 4 | 0.1 | 6.36 | 16.3 | 60.95 | 0.061 | 8.725 |
| 4000 | 675 | 20 | 30 | 44 | 4 | 0.1 | 6.36 | 16.3 | 60.95 | 0.061 | 8.725 |
| 5000 | 700 | 20 | 30 | 44 | 4 | 0.1 | 6.36 | 16.3 | 60.95 | 0.061 | 8.725 |
| 6000 | 725 | 20 | 30 | 44 | 4 | 0.1 | 6.36 | 16.3 | 60.95 | 0.061 | 8.725 |
| 7000 | 745 | 20 | 30 | 44 | 4 | 0.1 | 6.36 | 16.3 | 60.95 | 0.061 | 8.725 |
| 8000 | 760 | 20 | 30 | 44 | 4 | 0.1 | 6.36 | 16.3 | 60.95 | 0.061 | 8.725 |
| **N** | **E_p_** | **T** | **S** | **P** | **C** | **D** | **A** | **M** | **F** | **B** | **E** |
| 9000 | 775 | 20 | 30 | 44 | 4 | 0.1 | 6.36 | 16.3 | 60.95 | 0.061 | 8.725 |
| 10000 | 790 | 20 | 30 | 44 | 4 | 0.1 | 6.36 | 16.3 | 60.95 | 0.061 | 8.725 |
| 1 | 95 | 20 | 30 | 44 | 4.6 | 0.1 | 3.91 | 15.23 | 74.35 | 0.061 | 10.246 |
| 2 | 110 | 20 | 30 | 44 | 4.6 | 0.1 | 3.91 | 15.23 | 74.35 | 0.061 | 10.246 |
| 10 | 155 | 20 | 30 | 44 | 4.6 | 0.1 | 3.91 | 15.23 | 74.35 | 0.061 | 10.246 |
| 100 | 260 | 20 | 30 | 44 | 4.6 | 0.1 | 3.91 | 15.23 | 74.35 | 0.061 | 10.246 |
| 500 | 370 | 20 | 30 | 44 | 4.6 | 0.1 | 3.91 | 15.23 | 74.35 | 0.061 | 10.246 |
| 1000 | 430 | 20 | 30 | 44 | 4.6 | 0.1 | 3.91 | 15.23 | 74.35 | 0.061 | 10.246 |
| 2000 | 500 | 20 | 30 | 44 | 4.6 | 0.1 | 3.91 | 15.23 | 74.35 | 0.061 | 10.246 |
| 3000 | 545 | 20 | 30 | 44 | 4.6 | 0.1 | 3.91 | 15.23 | 74.35 | 0.061 | 10.246 |
| 4000 | 580 | 20 | 30 | 44 | 4.6 | 0.1 | 3.91 | 15.23 | 74.35 | 0.061 | 10.246 |
| 5000 | 610 | 20 | 30 | 44 | 4.6 | 0.1 | 3.91 | 15.23 | 74.35 | 0.061 | 10.246 |
| 6000 | 630 | 20 | 30 | 44 | 4.6 | 0.1 | 3.91 | 15.23 | 74.35 | 0.061 | 10.246 |
| 7000 | 650 | 20 | 30 | 44 | 4.6 | 0.1 | 3.91 | 15.23 | 74.35 | 0.061 | 10.246 |
| 8000 | 670 | 20 | 30 | 44 | 4.6 | 0.1 | 3.91 | 15.23 | 74.35 | 0.061 | 10.246 |
| 9000 | 690 | 20 | 30 | 44 | 4.6 | 0.1 | 3.91 | 15.23 | 74.35 | 0.061 | 10.246 |
| 10000 | 710 | 20 | 30 | 44 | 4.6 | 0.1 | 3.91 | 15.23 | 74.35 | 0.061 | 10.246 |
| 1 | 65 | 20 | 30 | 44 | 5.2 | 0.1 | 2.71 | 15 | 81.9 | 0.061 | 11.706 |
| 2 | 80 | 20 | 30 | 44 | 5.2 | 0.1 | 2.71 | 15 | 81.9 | 0.061 | 11.706 |
| 10 | 125 | 20 | 30 | 44 | 5.2 | 0.1 | 2.71 | 15 | 81.9 | 0.061 | 11.706 |
| 100 | 235 | 20 | 30 | 44 | 5.2 | 0.1 | 2.71 | 15 | 81.9 | 0.061 | 11.706 |
| 500 | 365 | 20 | 30 | 44 | 5.2 | 0.1 | 2.71 | 15 | 81.9 | 0.061 | 11.706 |
| 1000 | 445 | 20 | 30 | 44 | 5.2 | 0.1 | 2.71 | 15 | 81.9 | 0.061 | 11.706 |
| 2000 | 540 | 20 | 30 | 44 | 5.2 | 0.1 | 2.71 | 15 | 81.9 | 0.061 | 11.706 |
| 3000 | 600 | 20 | 30 | 44 | 5.2 | 0.1 | 2.71 | 15 | 81.9 | 0.061 | 11.706 |
| 4000 | 650 | 20 | 30 | 44 | 5.2 | 0.1 | 2.71 | 15 | 81.9 | 0.061 | 11.706 |
| 5000 | 695 | 20 | 30 | 44 | 5.2 | 0.1 | 2.71 | 15 | 81.9 | 0.061 | 11.706 |
| 6000 | 730 | 20 | 30 | 44 | 5.2 | 0.1 | 2.71 | 15 | 81.9 | 0.061 | 11.706 |
| 7000 | 760 | 20 | 30 | 44 | 5.2 | 0.1 | 2.71 | 15 | 81.9 | 0.061 | 11.706 |
| 8000 | 790 | 20 | 30 | 44 | 5.2 | 0.1 | 2.71 | 15 | 81.9 | 0.061 | 11.706 |
| 9000 | 820 | 20 | 30 | 44 | 5.2 | 0.1 | 2.71 | 15 | 81.9 | 0.061 | 11.706 |
| 10000 | 845 | 20 | 30 | 44 | 5.2 | 0.1 | 2.71 | 15 | 81.9 | 0.061 | 11.706 |
| 1 | 50 | 20 | 10 | 44 | 4 | 0.4 | 6.36 | 16.3 | 60.95 | 0.061 | 8.725 |
| 2 | 55 | 20 | 10 | 44 | 4 | 0.4 | 6.36 | 16.3 | 60.95 | 0.061 | 8.725 |
| 10 | 65 | 20 | 10 | 44 | 4 | 0.4 | 6.36 | 16.3 | 60.95 | 0.061 | 8.725 |
| 100 | 80 | 20 | 10 | 44 | 4 | 0.4 | 6.36 | 16.3 | 60.95 | 0.061 | 8.725 |
| 500 | 95 | 20 | 10 | 44 | 4 | 0.4 | 6.36 | 16.3 | 60.95 | 0.061 | 8.725 |
| 1000 | 100 | 20 | 10 | 44 | 4 | 0.4 | 6.36 | 16.3 | 60.95 | 0.061 | 8.725 |
| 2000 | 110 | 20 | 10 | 44 | 4 | 0.4 | 6.36 | 16.3 | 60.95 | 0.061 | 8.725 |
| 3000 | 115 | 20 | 10 | 44 | 4 | 0.4 | 6.36 | 16.3 | 60.95 | 0.061 | 8.725 |
| 4000 | 120 | 20 | 10 | 44 | 4 | 0.4 | 6.36 | 16.3 | 60.95 | 0.061 | 8.725 |
| 5000 | 120 | 20 | 10 | 44 | 4 | 0.4 | 6.36 | 16.3 | 60.95 | 0.061 | 8.725 |
| 6000 | 125 | 20 | 10 | 44 | 4 | 0.4 | 6.36 | 16.3 | 60.95 | 0.061 | 8.725 |
| 7000 | 125 | 20 | 10 | 44 | 4 | 0.4 | 6.36 | 16.3 | 60.95 | 0.061 | 8.725 |
| 8000 | 127 | 20 | 10 | 44 | 4 | 0.4 | 6.36 | 16.3 | 60.95 | 0.061 | 8.725 |
| 9000 | 129 | 20 | 10 | 44 | 4 | 0.4 | 6.36 | 16.3 | 60.95 | 0.061 | 8.725 |
| 10000 | 130 | 20 | 10 | 44 | 4 | 0.4 | 6.36 | 16.3 | 60.95 | 0.061 | 8.725 |
| 1 | 10 | 20 | 10 | 44 | 4.6 | 0.4 | 4.03 | 15.33 | 73.75 | 0.061 | 10.234 |
| 2 | 11 | 20 | 10 | 44 | 4.6 | 0.4 | 4.03 | 15.33 | 73.75 | 0.061 | 10.234 |
| 10 | 13 | 20 | 10 | 44 | 4.6 | 0.4 | 4.03 | 15.33 | 73.75 | 0.061 | 10.234 |
| 100 | 18 | 20 | 10 | 44 | 4.6 | 0.4 | 4.03 | 15.33 | 73.75 | 0.061 | 10.234 |
| 500 | 20 | 20 | 10 | 44 | 4.6 | 0.4 | 4.03 | 15.33 | 73.75 | 0.061 | 10.234 |
| **N** | **E_p_** | **T** | **S** | **P** | **C** | **D** | **A** | **M** | **F** | **B** | **E** |
| 1000 | 23 | 20 | 10 | 44 | 4.6 | 0.4 | 4.03 | 15.33 | 73.75 | 0.061 | 10.234 |
| 2000 | 25 | 20 | 10 | 44 | 4.6 | 0.4 | 4.03 | 15.33 | 73.75 | 0.061 | 10.234 |
| 3000 | 27 | 20 | 10 | 44 | 4.6 | 0.4 | 4.03 | 15.33 | 73.75 | 0.061 | 10.234 |
| 4000 | 27 | 20 | 10 | 44 | 4.6 | 0.4 | 4.03 | 15.33 | 73.75 | 0.061 | 10.234 |
| 5000 | 28 | 20 | 10 | 44 | 4.6 | 0.4 | 4.03 | 15.33 | 73.75 | 0.061 | 10.234 |
| 6000 | 29 | 20 | 10 | 44 | 4.6 | 0.4 | 4.03 | 15.33 | 73.75 | 0.061 | 10.234 |
| 7000 | 30 | 20 | 10 | 44 | 4.6 | 0.4 | 4.03 | 15.33 | 73.75 | 0.061 | 10.234 |
| 8000 | 30 | 20 | 10 | 44 | 4.6 | 0.4 | 4.03 | 15.33 | 73.75 | 0.061 | 10.234 |
| 9000 | 30 | 20 | 10 | 44 | 4.6 | 0.4 | 4.03 | 15.33 | 73.75 | 0.061 | 10.234 |
| 10000 | 31 | 20 | 10 | 44 | 4.6 | 0.4 | 4.03 | 15.33 | 73.75 | 0.061 | 10.234 |
| 1 | 35 | 20 | 10 | 44 | 5.2 | 0.4 | 3.01 | 15.25 | 80.27 | 0.061 | 11.671 |
| 2 | 43 | 20 | 10 | 44 | 5.2 | 0.4 | 3.01 | 15.25 | 80.27 | 0.061 | 11.671 |
| 10 | 70 | 20 | 10 | 44 | 5.2 | 0.4 | 3.01 | 15.25 | 80.27 | 0.061 | 11.671 |
| 100 | 140 | 20 | 10 | 44 | 5.2 | 0.4 | 3.01 | 15.25 | 80.27 | 0.061 | 11.671 |
| 500 | 225 | 20 | 10 | 44 | 5.2 | 0.4 | 3.01 | 15.25 | 80.27 | 0.061 | 11.671 |
| 1000 | 275 | 20 | 10 | 44 | 5.2 | 0.4 | 3.01 | 15.25 | 80.27 | 0.061 | 11.671 |
| 2000 | 335 | 20 | 10 | 44 | 5.2 | 0.4 | 3.01 | 15.25 | 80.27 | 0.061 | 11.671 |
| 3000 | 380 | 20 | 10 | 44 | 5.2 | 0.4 | 3.01 | 15.25 | 80.27 | 0.061 | 11.671 |
| 4000 | 415 | 20 | 10 | 44 | 5.2 | 0.4 | 3.01 | 15.25 | 80.27 | 0.061 | 11.671 |
| 5000 | 440 | 20 | 10 | 44 | 5.2 | 0.4 | 3.01 | 15.25 | 80.27 | 0.061 | 11.671 |
| 6000 | 460 | 20 | 10 | 44 | 5.2 | 0.4 | 3.01 | 15.25 | 80.27 | 0.061 | 11.671 |
| 7000 | 490 | 20 | 10 | 44 | 5.2 | 0.4 | 3.01 | 15.25 | 80.27 | 0.061 | 11.671 |
| 8000 | 510 | 20 | 10 | 44 | 5.2 | 0.4 | 3.01 | 15.25 | 80.27 | 0.061 | 11.671 |
| 9000 | 525 | 20 | 10 | 44 | 5.2 | 0.4 | 3.01 | 15.25 | 80.27 | 0.061 | 11.671 |
| 10000 | 545 | 20 | 10 | 44 | 5.2 | 0.4 | 3.01 | 15.25 | 80.27 | 0.061 | 11.671 |
| 1 | 180 | 20 | 20 | 44 | 4 | 0.4 | 6.44 | 16.37 | 60.65 | 0.061 | 8.718 |
| 2 | 205 | 20 | 20 | 44 | 4 | 0.4 | 6.44 | 16.37 | 60.65 | 0.061 | 8.718 |
| 10 | 280 | 20 | 20 | 44 | 4 | 0.4 | 6.44 | 16.37 | 60.65 | 0.061 | 8.718 |
| 100 | 430 | 20 | 20 | 44 | 4 | 0.4 | 6.44 | 16.37 | 60.65 | 0.061 | 8.718 |
| 500 | 580 | 20 | 20 | 44 | 4 | 0.4 | 6.44 | 16.37 | 60.65 | 0.061 | 8.718 |
| 1000 | 660 | 20 | 20 | 44 | 4 | 0.4 | 6.44 | 16.37 | 60.65 | 0.061 | 8.718 |
| 2000 | 750 | 20 | 20 | 44 | 4 | 0.4 | 6.44 | 16.37 | 60.65 | 0.061 | 8.718 |
| 3000 | 815 | 20 | 20 | 44 | 4 | 0.4 | 6.44 | 16.37 | 60.65 | 0.061 | 8.718 |
| 4000 | 860 | 20 | 20 | 44 | 4 | 0.4 | 6.44 | 16.37 | 60.65 | 0.061 | 8.718 |
| 5000 | 895 | 20 | 20 | 44 | 4 | 0.4 | 6.44 | 16.37 | 60.65 | 0.061 | 8.718 |
| 6000 | 925 | 20 | 20 | 44 | 4 | 0.4 | 6.44 | 16.37 | 60.65 | 0.061 | 8.718 |
| 7000 | 950 | 20 | 20 | 44 | 4 | 0.4 | 6.44 | 16.37 | 60.65 | 0.061 | 8.718 |
| 8000 | 980 | 20 | 20 | 44 | 4 | 0.4 | 6.44 | 16.37 | 60.65 | 0.061 | 8.718 |
| 9000 | 1000 | 20 | 20 | 44 | 4 | 0.4 | 6.44 | 16.37 | 60.65 | 0.061 | 8.718 |
| 10000 | 1025 | 20 | 20 | 44 | 4 | 0.4 | 6.44 | 16.37 | 60.65 | 0.061 | 8.718 |
| 1 | 70 | 20 | 20 | 44 | 4.6 | 0.4 | 4.07 | 15.38 | 73.51 | 0.061 | 10.229 |
| 2 | 80 | 20 | 20 | 44 | 4.6 | 0.4 | 4.07 | 15.38 | 73.51 | 0.061 | 10.229 |
| 10 | 120 | 20 | 20 | 44 | 4.6 | 0.4 | 4.07 | 15.38 | 73.51 | 0.061 | 10.229 |
| 100 | 205 | 20 | 20 | 44 | 4.6 | 0.4 | 4.07 | 15.38 | 73.51 | 0.061 | 10.229 |
| 500 | 300 | 20 | 20 | 44 | 4.6 | 0.4 | 4.07 | 15.38 | 73.51 | 0.061 | 10.229 |
| 1000 | 355 | 20 | 20 | 44 | 4.6 | 0.4 | 4.07 | 15.38 | 73.51 | 0.061 | 10.229 |
| 2000 | 415 | 20 | 20 | 44 | 4.6 | 0.4 | 4.07 | 15.38 | 73.51 | 0.061 | 10.229 |
| 3000 | 460 | 20 | 20 | 44 | 4.6 | 0.4 | 4.07 | 15.38 | 73.51 | 0.061 | 10.229 |
| 4000 | 490 | 20 | 20 | 44 | 4.6 | 0.4 | 4.07 | 15.38 | 73.51 | 0.061 | 10.229 |
| 5000 | 520 | 20 | 20 | 44 | 4.6 | 0.4 | 4.07 | 15.38 | 73.51 | 0.061 | 10.229 |
| 6000 | 540 | 20 | 20 | 44 | 4.6 | 0.4 | 4.07 | 15.38 | 73.51 | 0.061 | 10.229 |
| 7000 | 560 | 20 | 20 | 44 | 4.6 | 0.4 | 4.07 | 15.38 | 73.51 | 0.061 | 10.229 |
| **N** | **E_p_** | **T** | **S** | **P** | **C** | **D** | **A** | **M** | **F** | **B** | **E** |
| 8000 | 575 | 20 | 20 | 44 | 4.6 | 0.4 | 4.07 | 15.38 | 73.51 | 0.061 | 10.229 |
| 9000 | 595 | 20 | 20 | 44 | 4.6 | 0.4 | 4.07 | 15.38 | 73.51 | 0.061 | 10.229 |
| 10000 | 610 | 20 | 20 | 44 | 4.6 | 0.4 | 4.07 | 15.38 | 73.51 | 0.061 | 10.229 |
| 1 | 25 | 20 | 20 | 44 | 5.2 | 0.4 | 2.95 | 15.2 | 80.58 | 0.061 | 11.678 |
| 2 | 30 | 20 | 20 | 44 | 5.2 | 0.4 | 2.95 | 15.2 | 80.58 | 0.061 | 11.678 |
| 10 | 50 | 20 | 20 | 44 | 5.2 | 0.4 | 2.95 | 15.2 | 80.58 | 0.061 | 11.678 |
| 100 | 90 | 20 | 20 | 44 | 5.2 | 0.4 | 2.95 | 15.2 | 80.58 | 0.061 | 11.678 |
| 500 | 145 | 20 | 20 | 44 | 5.2 | 0.4 | 2.95 | 15.2 | 80.58 | 0.061 | 11.678 |
| 1000 | 180 | 20 | 20 | 44 | 5.2 | 0.4 | 2.95 | 15.2 | 80.58 | 0.061 | 11.678 |
| 2000 | 215 | 20 | 20 | 44 | 5.2 | 0.4 | 2.95 | 15.2 | 80.58 | 0.061 | 11.678 |
| 3000 | 240 | 20 | 20 | 44 | 5.2 | 0.4 | 2.95 | 15.2 | 80.58 | 0.061 | 11.678 |
| 4000 | 260 | 20 | 20 | 44 | 5.2 | 0.4 | 2.95 | 15.2 | 80.58 | 0.061 | 11.678 |
| 5000 | 270 | 20 | 20 | 44 | 5.2 | 0.4 | 2.95 | 15.2 | 80.58 | 0.061 | 11.678 |
| 6000 | 290 | 20 | 20 | 44 | 5.2 | 0.4 | 2.95 | 15.2 | 80.58 | 0.061 | 11.678 |
| 7000 | 305 | 20 | 20 | 44 | 5.2 | 0.4 | 2.95 | 15.2 | 80.58 | 0.061 | 11.678 |
| 8000 | 315 | 20 | 20 | 44 | 5.2 | 0.4 | 2.95 | 15.2 | 80.58 | 0.061 | 11.678 |
| 9000 | 330 | 20 | 20 | 44 | 5.2 | 0.4 | 2.95 | 15.2 | 80.58 | 0.061 | 11.678 |
| 10000 | 340 | 20 | 20 | 44 | 5.2 | 0.4 | 2.95 | 15.2 | 80.58 | 0.061 | 11.678 |
| 1 | 310 | 20 | 30 | 44 | 4 | 0.4 | 6.34 | 16.28 | 61.06 | 0.061 | 8.727 |
| 2 | 375 | 20 | 30 | 44 | 4 | 0.4 | 6.34 | 16.28 | 61.06 | 0.061 | 8.727 |
| 10 | 580 | 20 | 30 | 44 | 4 | 0.4 | 6.34 | 16.28 | 61.06 | 0.061 | 8.727 |
| 100 | 1095 | 20 | 30 | 44 | 4 | 0.4 | 6.34 | 16.28 | 61.06 | 0.061 | 8.727 |
| 500 | 1705 | 20 | 30 | 44 | 4 | 0.4 | 6.34 | 16.28 | 61.06 | 0.061 | 8.727 |
| 1000 | 2060 | 20 | 30 | 44 | 4 | 0.4 | 6.34 | 16.28 | 61.06 | 0.061 | 8.727 |
| 2000 | 2495 | 20 | 30 | 44 | 4 | 0.4 | 6.34 | 16.28 | 61.06 | 0.061 | 8.727 |
| 3000 | 2780 | 20 | 30 | 44 | 4 | 0.4 | 6.34 | 16.28 | 61.06 | 0.061 | 8.727 |
| 4000 | 3015 | 20 | 30 | 44 | 4 | 0.4 | 6.34 | 16.28 | 61.06 | 0.061 | 8.727 |
| 5000 | 3200 | 20 | 30 | 44 | 4 | 0.4 | 6.34 | 16.28 | 61.06 | 0.061 | 8.727 |
| 6000 | 3370 | 20 | 30 | 44 | 4 | 0.4 | 6.34 | 16.28 | 61.06 | 0.061 | 8.727 |
| 7000 | 3515 | 20 | 30 | 44 | 4 | 0.4 | 6.34 | 16.28 | 61.06 | 0.061 | 8.727 |
| 8000 | 3645 | 20 | 30 | 44 | 4 | 0.4 | 6.34 | 16.28 | 61.06 | 0.061 | 8.727 |
| 9000 | 3765 | 20 | 30 | 44 | 4 | 0.4 | 6.34 | 16.28 | 61.06 | 0.061 | 8.727 |
| 10000 | 3875 | 20 | 30 | 44 | 4 | 0.4 | 6.34 | 16.28 | 61.06 | 0.061 | 8.727 |
| 1 | 180 | 20 | 30 | 44 | 4.6 | 0.4 | 4.07 | 15.38 | 73.51 | 0.061 | 10.229 |
| 2 | 225 | 20 | 30 | 44 | 4.6 | 0.4 | 4.07 | 15.38 | 73.51 | 0.061 | 10.229 |
| 10 | 380 | 20 | 30 | 44 | 4.6 | 0.4 | 4.07 | 15.38 | 73.51 | 0.061 | 10.229 |
| 100 | 805 | 20 | 30 | 44 | 4.6 | 0.4 | 4.07 | 15.38 | 73.51 | 0.061 | 10.229 |
| 500 | 1360 | 20 | 30 | 44 | 4.6 | 0.4 | 4.07 | 15.38 | 73.51 | 0.061 | 10.229 |
| 1000 | 1700 | 20 | 30 | 44 | 4.6 | 0.4 | 4.07 | 15.38 | 73.51 | 0.061 | 10.229 |
| 2000 | 2130 | 20 | 30 | 44 | 4.6 | 0.4 | 4.07 | 15.38 | 73.51 | 0.061 | 10.229 |
| 3000 | 2430 | 20 | 30 | 44 | 4.6 | 0.4 | 4.07 | 15.38 | 73.51 | 0.061 | 10.229 |
| 4000 | 2670 | 20 | 30 | 44 | 4.6 | 0.4 | 4.07 | 15.38 | 73.51 | 0.061 | 10.229 |
| 5000 | 2870 | 20 | 30 | 44 | 4.6 | 0.4 | 4.07 | 15.38 | 73.51 | 0.061 | 10.229 |
| 6000 | 3050 | 20 | 30 | 44 | 4.6 | 0.4 | 4.07 | 15.38 | 73.51 | 0.061 | 10.229 |
| 7000 | 3205 | 20 | 30 | 44 | 4.6 | 0.4 | 4.07 | 15.38 | 73.51 | 0.061 | 10.229 |
| 8000 | 3350 | 20 | 30 | 44 | 4.6 | 0.4 | 4.07 | 15.38 | 73.51 | 0.061 | 10.229 |
| 9000 | 3480 | 20 | 30 | 44 | 4.6 | 0.4 | 4.07 | 15.38 | 73.51 | 0.061 | 10.229 |
| 10000 | 3600 | 20 | 30 | 44 | 4.6 | 0.4 | 4.07 | 15.38 | 73.51 | 0.061 | 10.229 |
| 1 | 85 | 20 | 30 | 44 | 5.2 | 0.4 | 2.71 | 15 | 81.9 | 0.061 | 11.706 |
| 2 | 110 | 20 | 30 | 44 | 5.2 | 0.4 | 2.71 | 15 | 81.9 | 0.061 | 11.706 |
| 10 | 205 | 20 | 30 | 44 | 5.2 | 0.4 | 2.71 | 15 | 81.9 | 0.061 | 11.706 |
| 100 | 495 | 20 | 30 | 44 | 5.2 | 0.4 | 2.71 | 15 | 81.9 | 0.061 | 11.706 |
| **N** | **E_p_** | **T** | **S** | **P** | **C** | **D** | **A** | **M** | **F** | **B** | **E** |
| 500 | 910 | 20 | 30 | 44 | 5.2 | 0.4 | 2.71 | 15 | 81.9 | 0.061 | 11.706 |
| 1000 | 1180 | 20 | 30 | 44 | 5.2 | 0.4 | 2.71 | 15 | 81.9 | 0.061 | 11.706 |
| 2000 | 1545 | 20 | 30 | 44 | 5.2 | 0.4 | 2.71 | 15 | 81.9 | 0.061 | 11.706 |
| 3000 | 1800 | 20 | 30 | 44 | 5.2 | 0.4 | 2.71 | 15 | 81.9 | 0.061 | 11.706 |
| 4000 | 2015 | 20 | 30 | 44 | 5.2 | 0.4 | 2.71 | 15 | 81.9 | 0.061 | 11.706 |
| 5000 | 2195 | 20 | 30 | 44 | 5.2 | 0.4 | 2.71 | 15 | 81.9 | 0.061 | 11.706 |
| 6000 | 2355 | 20 | 30 | 44 | 5.2 | 0.4 | 2.71 | 15 | 81.9 | 0.061 | 11.706 |
| 7000 | 2495 | 20 | 30 | 44 | 5.2 | 0.4 | 2.71 | 15 | 81.9 | 0.061 | 11.706 |
| 8000 | 2620 | 20 | 30 | 44 | 5.2 | 0.4 | 2.71 | 15 | 81.9 | 0.061 | 11.706 |
| 9000 | 2740 | 20 | 30 | 44 | 5.2 | 0.4 | 2.71 | 15 | 81.9 | 0.061 | 11.706 |
| 10000 | 2860 | 20 | 30 | 44 | 5.2 | 0.4 | 2.71 | 15 | 81.9 | 0.061 | 11.706 |
| 1 | 15 | 20 | 10 | 65 | 4 | 0.1 | 6.5 | 17.1 | 62 | 0.06 | 8.753 |
| 2 | 16 | 20 | 10 | 65 | 4 | 0.1 | 6.5 | 17.1 | 62 | 0.06 | 8.753 |
| 10 | 18 | 20 | 10 | 65 | 4 | 0.1 | 6.5 | 17.1 | 62 | 0.06 | 8.753 |
| 100 | 22 | 20 | 10 | 65 | 4 | 0.1 | 6.5 | 17.1 | 62 | 0.06 | 8.753 |
| 500 | 25 | 20 | 10 | 65 | 4 | 0.1 | 6.5 | 17.1 | 62 | 0.06 | 8.753 |
| 1000 | 25 | 20 | 10 | 65 | 4 | 0.1 | 6.5 | 17.1 | 62 | 0.06 | 8.753 |
| 2000 | 27 | 20 | 10 | 65 | 4 | 0.1 | 6.5 | 17.1 | 62 | 0.06 | 8.753 |
| 3000 | 28 | 20 | 10 | 65 | 4 | 0.1 | 6.5 | 17.1 | 62 | 0.06 | 8.753 |
| 4000 | 29 | 20 | 10 | 65 | 4 | 0.1 | 6.5 | 17.1 | 62 | 0.06 | 8.753 |
| 5000 | 29 | 20 | 10 | 65 | 4 | 0.1 | 6.5 | 17.1 | 62 | 0.06 | 8.753 |
| 6000 | 30 | 20 | 10 | 65 | 4 | 0.1 | 6.5 | 17.1 | 62 | 0.06 | 8.753 |
| 7000 | 30 | 20 | 10 | 65 | 4 | 0.1 | 6.5 | 17.1 | 62 | 0.06 | 8.753 |
| 8000 | 30 | 20 | 10 | 65 | 4 | 0.1 | 6.5 | 17.1 | 62 | 0.06 | 8.753 |
| 9000 | 31 | 20 | 10 | 65 | 4 | 0.1 | 6.5 | 17.1 | 62 | 0.06 | 8.753 |
| 10000 | 31 | 20 | 10 | 65 | 4 | 0.1 | 6.5 | 17.1 | 62 | 0.06 | 8.753 |
| 1 | 15 | 20 | 10 | 65 | 4.6 | 0.1 | 4.28 | 15.97 | 73.22 | 0.06 | 10.287 |
| 2 | 16 | 20 | 10 | 65 | 4.6 | 0.1 | 4.28 | 15.97 | 73.22 | 0.06 | 10.287 |
| 10 | 19 | 20 | 10 | 65 | 4.6 | 0.1 | 4.28 | 15.97 | 73.22 | 0.06 | 10.287 |
| 100 | 25 | 20 | 10 | 65 | 4.6 | 0.1 | 4.28 | 15.97 | 73.22 | 0.06 | 10.287 |
| 500 | 28 | 20 | 10 | 65 | 4.6 | 0.1 | 4.28 | 15.97 | 73.22 | 0.06 | 10.287 |
| 1000 | 30 | 20 | 10 | 65 | 4.6 | 0.1 | 4.28 | 15.97 | 73.22 | 0.06 | 10.287 |
| 2000 | 32 | 20 | 10 | 65 | 4.6 | 0.1 | 4.28 | 15.97 | 73.22 | 0.06 | 10.287 |
| 3000 | 33 | 20 | 10 | 65 | 4.6 | 0.1 | 4.28 | 15.97 | 73.22 | 0.06 | 10.287 |
| 4000 | 34 | 20 | 10 | 65 | 4.6 | 0.1 | 4.28 | 15.97 | 73.22 | 0.06 | 10.287 |
| 5000 | 35 | 20 | 10 | 65 | 4.6 | 0.1 | 4.28 | 15.97 | 73.22 | 0.06 | 10.287 |
| 6000 | 35 | 20 | 10 | 65 | 4.6 | 0.1 | 4.28 | 15.97 | 73.22 | 0.06 | 10.287 |
| 7000 | 36 | 20 | 10 | 65 | 4.6 | 0.1 | 4.28 | 15.97 | 73.22 | 0.06 | 10.287 |
| 8000 | 37 | 20 | 10 | 65 | 4.6 | 0.1 | 4.28 | 15.97 | 73.22 | 0.06 | 10.287 |
| 9000 | 37 | 20 | 10 | 65 | 4.6 | 0.1 | 4.28 | 15.97 | 73.22 | 0.06 | 10.287 |
| 10000 | 37 | 20 | 10 | 65 | 4.6 | 0.1 | 4.28 | 15.97 | 73.22 | 0.06 | 10.287 |
| 1 | 10 | 20 | 10 | 65 | 5.2 | 0.1 | 2.83 | 15.55 | 81.81 | 0.06 | 11.78 |
| 2 | 11 | 20 | 10 | 65 | 5.2 | 0.1 | 2.83 | 15.55 | 81.81 | 0.06 | 11.78 |
| 10 | 13 | 20 | 10 | 65 | 5.2 | 0.1 | 2.83 | 15.55 | 81.81 | 0.06 | 11.78 |
| 100 | 17 | 20 | 10 | 65 | 5.2 | 0.1 | 2.83 | 15.55 | 81.81 | 0.06 | 11.78 |
| 500 | 20 | 20 | 10 | 65 | 5.2 | 0.1 | 2.83 | 15.55 | 81.81 | 0.06 | 11.78 |
| 1000 | 22 | 20 | 10 | 65 | 5.2 | 0.1 | 2.83 | 15.55 | 81.81 | 0.06 | 11.78 |
| 2000 | 25 | 20 | 10 | 65 | 5.2 | 0.1 | 2.83 | 15.55 | 81.81 | 0.06 | 11.78 |
| 3000 | 25 | 20 | 10 | 65 | 5.2 | 0.1 | 2.83 | 15.55 | 81.81 | 0.06 | 11.78 |
| 4000 | 26 | 20 | 10 | 65 | 5.2 | 0.1 | 2.83 | 15.55 | 81.81 | 0.06 | 11.78 |
| 5000 | 26 | 20 | 10 | 65 | 5.2 | 0.1 | 2.83 | 15.55 | 81.81 | 0.06 | 11.78 |
| 6000 | 27 | 20 | 10 | 65 | 5.2 | 0.1 | 2.83 | 15.55 | 81.81 | 0.06 | 11.78 |
| **N** | **E_p_** | **T** | **S** | **P** | **C** | **D** | **A** | **M** | **F** | **B** | **E** |
| 7000 | 27 | 20 | 10 | 65 | 5.2 | 0.1 | 2.83 | 15.55 | 81.81 | 0.06 | 11.78 |
| 8000 | 28 | 20 | 10 | 65 | 5.2 | 0.1 | 2.83 | 15.55 | 81.81 | 0.06 | 11.78 |
| 9000 | 28 | 20 | 10 | 65 | 5.2 | 0.1 | 2.83 | 15.55 | 81.81 | 0.06 | 11.78 |
| 10000 | 29 | 20 | 10 | 65 | 5.2 | 0.1 | 2.83 | 15.55 | 81.81 | 0.06 | 11.78 |
| 1 | 115 | 20 | 20 | 65 | 4 | 0.1 | 6.36 | 16.97 | 62.55 | 0.06 | 8.766 |
| 2 | 125 | 20 | 20 | 65 | 4 | 0.1 | 6.36 | 16.97 | 62.55 | 0.06 | 8.766 |
| 10 | 155 | 20 | 20 | 65 | 4 | 0.1 | 6.36 | 16.97 | 62.55 | 0.06 | 8.766 |
| 100 | 215 | 20 | 20 | 65 | 4 | 0.1 | 6.36 | 16.97 | 62.55 | 0.06 | 8.766 |
| 500 | 270 | 20 | 20 | 65 | 4 | 0.1 | 6.36 | 16.97 | 62.55 | 0.06 | 8.766 |
| 1000 | 295 | 20 | 20 | 65 | 4 | 0.1 | 6.36 | 16.97 | 62.55 | 0.06 | 8.766 |
| 2000 | 325 | 20 | 20 | 65 | 4 | 0.1 | 6.36 | 16.97 | 62.55 | 0.06 | 8.766 |
| 3000 | 340 | 20 | 20 | 65 | 4 | 0.1 | 6.36 | 16.97 | 62.55 | 0.06 | 8.766 |
| 4000 | 355 | 20 | 20 | 65 | 4 | 0.1 | 6.36 | 16.97 | 62.55 | 0.06 | 8.766 |
| 5000 | 365 | 20 | 20 | 65 | 4 | 0.1 | 6.36 | 16.97 | 62.55 | 0.06 | 8.766 |
| 6000 | 375 | 20 | 20 | 65 | 4 | 0.1 | 6.36 | 16.97 | 62.55 | 0.06 | 8.766 |
| 7000 | 380 | 20 | 20 | 65 | 4 | 0.1 | 6.36 | 16.97 | 62.55 | 0.06 | 8.766 |
| 8000 | 390 | 20 | 20 | 65 | 4 | 0.1 | 6.36 | 16.97 | 62.55 | 0.06 | 8.766 |
| 9000 | 395 | 20 | 20 | 65 | 4 | 0.1 | 6.36 | 16.97 | 62.55 | 0.06 | 8.766 |
| 10000 | 400 | 20 | 20 | 65 | 4 | 0.1 | 6.36 | 16.97 | 62.55 | 0.06 | 8.766 |
| 1 | 50 | 20 | 20 | 65 | 4.6 | 0.1 | 4.36 | 16.04 | 72.82 | 0.06 | 10.278 |
| 2 | 55 | 20 | 20 | 65 | 4.6 | 0.1 | 4.36 | 16.04 | 72.82 | 0.06 | 10.278 |
| 10 | 75 | 20 | 20 | 65 | 4.6 | 0.1 | 4.36 | 16.04 | 72.82 | 0.06 | 10.278 |
| 100 | 110 | 20 | 20 | 65 | 4.6 | 0.1 | 4.36 | 16.04 | 72.82 | 0.06 | 10.278 |
| 500 | 155 | 20 | 20 | 65 | 4.6 | 0.1 | 4.36 | 16.04 | 72.82 | 0.06 | 10.278 |
| 1000 | 175 | 20 | 20 | 65 | 4.6 | 0.1 | 4.36 | 16.04 | 72.82 | 0.06 | 10.278 |
| 2000 | 200 | 20 | 20 | 65 | 4.6 | 0.1 | 4.36 | 16.04 | 72.82 | 0.06 | 10.278 |
| 3000 | 215 | 20 | 20 | 65 | 4.6 | 0.1 | 4.36 | 16.04 | 72.82 | 0.06 | 10.278 |
| 4000 | 230 | 20 | 20 | 65 | 4.6 | 0.1 | 4.36 | 16.04 | 72.82 | 0.06 | 10.278 |
| 5000 | 235 | 20 | 20 | 65 | 4.6 | 0.1 | 4.36 | 16.04 | 72.82 | 0.06 | 10.278 |
| 6000 | 245 | 20 | 20 | 65 | 4.6 | 0.1 | 4.36 | 16.04 | 72.82 | 0.06 | 10.278 |
| 7000 | 250 | 20 | 20 | 65 | 4.6 | 0.1 | 4.36 | 16.04 | 72.82 | 0.06 | 10.278 |
| 8000 | 260 | 20 | 20 | 65 | 4.6 | 0.1 | 4.36 | 16.04 | 72.82 | 0.06 | 10.278 |
| 9000 | 260 | 20 | 20 | 65 | 4.6 | 0.1 | 4.36 | 16.04 | 72.82 | 0.06 | 10.278 |
| 10000 | 270 | 20 | 20 | 65 | 4.6 | 0.1 | 4.36 | 16.04 | 72.82 | 0.06 | 10.278 |
| 1 | 40 | 20 | 20 | 65 | 5.2 | 0.1 | 2.62 | 15.37 | 82.97 | 0.06 | 11.805 |
| 2 | 45 | 20 | 20 | 65 | 5.2 | 0.1 | 2.62 | 15.37 | 82.97 | 0.06 | 11.805 |
| 10 | 70 | 20 | 20 | 65 | 5.2 | 0.1 | 2.62 | 15.37 | 82.97 | 0.06 | 11.805 |
| 100 | 120 | 20 | 20 | 65 | 5.2 | 0.1 | 2.62 | 15.37 | 82.97 | 0.06 | 11.805 |
| 500 | 170 | 20 | 20 | 65 | 5.2 | 0.1 | 2.62 | 15.37 | 82.97 | 0.06 | 11.805 |
| 1000 | 200 | 20 | 20 | 65 | 5.2 | 0.1 | 2.62 | 15.37 | 82.97 | 0.06 | 11.805 |
| 2000 | 235 | 20 | 20 | 65 | 5.2 | 0.1 | 2.62 | 15.37 | 82.97 | 0.06 | 11.805 |
| 3000 | 260 | 20 | 20 | 65 | 5.2 | 0.1 | 2.62 | 15.37 | 82.97 | 0.06 | 11.805 |
| 4000 | 280 | 20 | 20 | 65 | 5.2 | 0.1 | 2.62 | 15.37 | 82.97 | 0.06 | 11.805 |
| 5000 | 290 | 20 | 20 | 65 | 5.2 | 0.1 | 2.62 | 15.37 | 82.97 | 0.06 | 11.805 |
| 6000 | 305 | 20 | 20 | 65 | 5.2 | 0.1 | 2.62 | 15.37 | 82.97 | 0.06 | 11.805 |
| 7000 | 315 | 20 | 20 | 65 | 5.2 | 0.1 | 2.62 | 15.37 | 82.97 | 0.06 | 11.805 |
| 8000 | 330 | 20 | 20 | 65 | 5.2 | 0.1 | 2.62 | 15.37 | 82.97 | 0.06 | 11.805 |
| 9000 | 340 | 20 | 20 | 65 | 5.2 | 0.1 | 2.62 | 15.37 | 82.97 | 0.06 | 11.805 |
| 10000 | 350 | 20 | 20 | 65 | 5.2 | 0.1 | 2.62 | 15.37 | 82.97 | 0.06 | 11.805 |
| 1 | 320 | 20 | 30 | 65 | 4 | 0.1 | 6.33 | 16.95 | 62.66 | 0.06 | 8.769 |
| 2 | 360 | 20 | 30 | 65 | 4 | 0.1 | 6.33 | 16.95 | 62.66 | 0.06 | 8.769 |
| 10 | 475 | 20 | 30 | 65 | 4 | 0.1 | 6.33 | 16.95 | 62.66 | 0.06 | 8.769 |
| **N** | **E_p_** | **T** | **S** | **P** | **C** | **D** | **A** | **M** | **F** | **B** | **E** |
| 100 | 705 | 20 | 30 | 65 | 4 | 0.1 | 6.33 | 16.95 | 62.66 | 0.06 | 8.769 |
| 500 | 930 | 20 | 30 | 65 | 4 | 0.1 | 6.33 | 16.95 | 62.66 | 0.06 | 8.769 |
| 1000 | 7045 | 20 | 30 | 65 | 4 | 0.1 | 6.33 | 16.95 | 62.66 | 0.06 | 8.769 |
| 2000 | 1180 | 20 | 30 | 65 | 4 | 0.1 | 6.33 | 16.95 | 62.66 | 0.06 | 8.769 |
| 3000 | 1265 | 20 | 30 | 65 | 4 | 0.1 | 6.33 | 16.95 | 62.66 | 0.06 | 8.769 |
| 4000 | 1330 | 20 | 30 | 65 | 4 | 0.1 | 6.33 | 16.95 | 62.66 | 0.06 | 8.769 |
| 5000 | 1380 | 20 | 30 | 65 | 4 | 0.1 | 6.33 | 16.95 | 62.66 | 0.06 | 8.769 |
| 6000 | 1425 | 20 | 30 | 65 | 4 | 0.1 | 6.33 | 16.95 | 62.66 | 0.06 | 8.769 |
| 7000 | 1460 | 20 | 30 | 65 | 4 | 0.1 | 6.33 | 16.95 | 62.66 | 0.06 | 8.769 |
| 8000 | 1500 | 20 | 30 | 65 | 4 | 0.1 | 6.33 | 16.95 | 62.66 | 0.06 | 8.769 |
| 9000 | 1530 | 20 | 30 | 65 | 4 | 0.1 | 6.33 | 16.95 | 62.66 | 0.06 | 8.769 |
| 10000 | 1560 | 20 | 30 | 65 | 4 | 0.1 | 6.33 | 16.95 | 62.66 | 0.06 | 8.769 |
| 1 | 240 | 20 | 30 | 65 | 4.6 | 0.1 | 4.36 | 16.04 | 72.82 | 0.06 | 10.278 |
| 2 | 280 | 20 | 30 | 65 | 4.6 | 0.1 | 4.36 | 16.04 | 72.82 | 0.06 | 10.278 |
| 10 | 400 | 20 | 30 | 65 | 4.6 | 0.1 | 4.36 | 16.04 | 72.82 | 0.06 | 10.278 |
| 100 | 665 | 20 | 30 | 65 | 4.6 | 0.1 | 4.36 | 16.04 | 72.82 | 0.06 | 10.278 |
| 500 | 945 | 20 | 30 | 65 | 4.6 | 0.1 | 4.36 | 16.04 | 72.82 | 0.06 | 10.278 |
| 1000 | 1105 | 20 | 30 | 65 | 4.6 | 0.1 | 4.36 | 16.04 | 72.82 | 0.06 | 10.278 |
| 2000 | 1290 | 20 | 30 | 65 | 4.6 | 0.1 | 4.36 | 16.04 | 72.82 | 0.06 | 10.278 |
| 3000 | 1405 | 20 | 30 | 65 | 4.6 | 0.1 | 4.36 | 16.04 | 72.82 | 0.06 | 10.278 |
| 4000 | 1500 | 20 | 30 | 65 | 4.6 | 0.1 | 4.36 | 16.04 | 72.82 | 0.06 | 10.278 |
| 5000 | 1575 | 20 | 30 | 65 | 4.6 | 0.1 | 4.36 | 16.04 | 72.82 | 0.06 | 10.278 |
| 6000 | 1640 | 20 | 30 | 65 | 4.6 | 0.1 | 4.36 | 16.04 | 72.82 | 0.06 | 10.278 |
| 7000 | 1690 | 20 | 30 | 65 | 4.6 | 0.1 | 4.36 | 16.04 | 72.82 | 0.06 | 10.278 |
| 8000 | 1750 | 20 | 30 | 65 | 4.6 | 0.1 | 4.36 | 16.04 | 72.82 | 0.06 | 10.278 |
| 9000 | 1800 | 20 | 30 | 65 | 4.6 | 0.1 | 4.36 | 16.04 | 72.82 | 0.06 | 10.278 |
| 10000 | 1840 | 20 | 30 | 65 | 4.6 | 0.1 | 4.36 | 16.04 | 72.82 | 0.06 | 10.278 |
| 1 | 320 | 20 | 30 | 65 | 5.2 | 0.1 | 2.62 | 15.37 | 82.97 | 0.06 | 11.805 |
| 2 | 390 | 20 | 30 | 65 | 5.2 | 0.1 | 2.62 | 15.37 | 82.97 | 0.06 | 11.805 |
| 10 | 610 | 20 | 30 | 65 | 5.2 | 0.1 | 2.62 | 15.37 | 82.97 | 0.06 | 11.805 |
| 100 | 1170 | 20 | 30 | 65 | 5.2 | 0.1 | 2.62 | 15.37 | 82.97 | 0.06 | 11.805 |
| 500 | 1840 | 20 | 30 | 65 | 5.2 | 0.1 | 2.62 | 15.37 | 82.97 | 0.06 | 11.805 |
| 1000 | 2230 | 20 | 30 | 65 | 5.2 | 0.1 | 2.62 | 15.37 | 82.97 | 0.06 | 11.805 |
| 2000 | 2720 | 20 | 30 | 65 | 5.2 | 0.1 | 2.62 | 15.37 | 82.97 | 0.06 | 11.805 |
| 3000 | 3050 | 20 | 30 | 65 | 5.2 | 0.1 | 2.62 | 15.37 | 82.97 | 0.06 | 11.805 |
| 4000 | 3300 | 20 | 30 | 65 | 5.2 | 0.1 | 2.62 | 15.37 | 82.97 | 0.06 | 11.805 |
| 5000 | 3520 | 20 | 30 | 65 | 5.2 | 0.1 | 2.62 | 15.37 | 82.97 | 0.06 | 11.805 |
| 6000 | 3700 | 20 | 30 | 65 | 5.2 | 0.1 | 2.62 | 15.37 | 82.97 | 0.06 | 11.805 |
| 7000 | 3870 | 20 | 30 | 65 | 5.2 | 0.1 | 2.62 | 15.37 | 82.97 | 0.06 | 11.805 |
| 8000 | 4020 | 20 | 30 | 65 | 5.2 | 0.1 | 2.62 | 15.37 | 82.97 | 0.06 | 11.805 |
| 9000 | 4150 | 20 | 30 | 65 | 5.2 | 0.1 | 2.62 | 15.37 | 82.97 | 0.06 | 11.805 |
| 10000 | 4280 | 20 | 30 | 65 | 5.2 | 0.1 | 2.62 | 15.37 | 82.97 | 0.06 | 11.805 |
| 1 | 20 | 20 | 10 | 65 | 4 | 0.4 | 6.66 | 17.25 | 61.36 | 0.06 | 8.737 |
| 2 | 22 | 20 | 10 | 65 | 4 | 0.4 | 6.66 | 17.25 | 61.36 | 0.06 | 8.737 |
| 10 | 25 | 20 | 10 | 65 | 4 | 0.4 | 6.66 | 17.25 | 61.36 | 0.06 | 8.737 |
| 100 | 30 | 20 | 10 | 65 | 4 | 0.4 | 6.66 | 17.25 | 61.36 | 0.06 | 8.737 |
| 500 | 40 | 20 | 10 | 65 | 4 | 0.4 | 6.66 | 17.25 | 61.36 | 0.06 | 8.737 |
| 1000 | 42 | 20 | 10 | 65 | 4 | 0.4 | 6.66 | 17.25 | 61.36 | 0.06 | 8.737 |
| 2000 | 45 | 20 | 10 | 65 | 4 | 0.4 | 6.66 | 17.25 | 61.36 | 0.06 | 8.737 |
| 3000 | 47 | 20 | 10 | 65 | 4 | 0.4 | 6.66 | 17.25 | 61.36 | 0.06 | 8.737 |
| 4000 | 48 | 20 | 10 | 65 | 4 | 0.4 | 6.66 | 17.25 | 61.36 | 0.06 | 8.737 |
| 5000 | 49 | 20 | 10 | 65 | 4 | 0.4 | 6.66 | 17.25 | 61.36 | 0.06 | 8.737 |
| **N** | **E_p_** | **T** | **S** | **P** | **C** | **D** | **A** | **M** | **F** | **B** | **E** |
| 6000 | 50 | 20 | 10 | 65 | 4 | 0.4 | 6.66 | 17.25 | 61.36 | 0.06 | 8.737 |
| 7000 | 51 | 20 | 10 | 65 | 4 | 0.4 | 6.66 | 17.25 | 61.36 | 0.06 | 8.737 |
| 8000 | 52 | 20 | 10 | 65 | 4 | 0.4 | 6.66 | 17.25 | 61.36 | 0.06 | 8.737 |
| 9000 | 52 | 20 | 10 | 65 | 4 | 0.4 | 6.66 | 17.25 | 61.36 | 0.06 | 8.737 |
| 10000 | 53 | 20 | 10 | 65 | 4 | 0.4 | 6.66 | 17.25 | 61.36 | 0.06 | 8.737 |
| 1 | 25 | 20 | 10 | 65 | 4.6 | 0.4 | 4.44 | 16.11 | 72.45 | 0.06 | 10.27 |
| 2 | 27 | 20 | 10 | 65 | 4.6 | 0.4 | 4.44 | 16.11 | 72.45 | 0.06 | 10.27 |
| 10 | 33 | 20 | 10 | 65 | 4.6 | 0.4 | 4.44 | 16.11 | 72.45 | 0.06 | 10.27 |
| 100 | 45 | 20 | 10 | 65 | 4.6 | 0.4 | 4.44 | 16.11 | 72.45 | 0.06 | 10.27 |
| 500 | 55 | 20 | 10 | 65 | 4.6 | 0.4 | 4.44 | 16.11 | 72.45 | 0.06 | 10.27 |
| 1000 | 60 | 20 | 10 | 65 | 4.6 | 0.4 | 4.44 | 16.11 | 72.45 | 0.06 | 10.27 |
| 2000 | 65 | 20 | 10 | 65 | 4.6 | 0.4 | 4.44 | 16.11 | 72.45 | 0.06 | 10.27 |
| 3000 | 68 | 20 | 10 | 65 | 4.6 | 0.4 | 4.44 | 16.11 | 72.45 | 0.06 | 10.27 |
| 4000 | 70 | 20 | 10 | 65 | 4.6 | 0.4 | 4.44 | 16.11 | 72.45 | 0.06 | 10.27 |
| 5000 | 72 | 20 | 10 | 65 | 4.6 | 0.4 | 4.44 | 16.11 | 72.45 | 0.06 | 10.27 |
| 6000 | 74 | 20 | 10 | 65 | 4.6 | 0.4 | 4.44 | 16.11 | 72.45 | 0.06 | 10.27 |
| 7000 | 75 | 20 | 10 | 65 | 4.6 | 0.4 | 4.44 | 16.11 | 72.45 | 0.06 | 10.27 |
| 8000 | 76 | 20 | 10 | 65 | 4.6 | 0.4 | 4.44 | 16.11 | 72.45 | 0.06 | 10.27 |
| 9000 | 77 | 20 | 10 | 65 | 4.6 | 0.4 | 4.44 | 16.11 | 72.45 | 0.06 | 10.27 |
| 10000 | 78 | 20 | 10 | 65 | 4.6 | 0.4 | 4.44 | 16.11 | 72.45 | 0.06 | 10.27 |
| 1 | 15 | 20 | 10 | 65 | 5.2 | 0.4 | 2.8 | 15.52 | 81.98 | 0.06 | 11.783 |
| 2 | 20 | 20 | 10 | 65 | 5.2 | 0.4 | 2.8 | 15.52 | 81.98 | 0.06 | 11.783 |
| 10 | 30 | 20 | 10 | 65 | 5.2 | 0.4 | 2.8 | 15.52 | 81.98 | 0.06 | 11.783 |
| 100 | 60 | 20 | 10 | 65 | 5.2 | 0.4 | 2.8 | 15.52 | 81.98 | 0.06 | 11.783 |
| 500 | 100 | 20 | 10 | 65 | 5.2 | 0.4 | 2.8 | 15.52 | 81.98 | 0.06 | 11.783 |
| 1000 | 120 | 20 | 10 | 65 | 5.2 | 0.4 | 2.8 | 15.52 | 81.98 | 0.06 | 11.783 |
| 2000 | 145 | 20 | 10 | 65 | 5.2 | 0.4 | 2.8 | 15.52 | 81.98 | 0.06 | 11.783 |
| 3000 | 165 | 20 | 10 | 65 | 5.2 | 0.4 | 2.8 | 15.52 | 81.98 | 0.06 | 11.783 |
| 4000 | 180 | 20 | 10 | 65 | 5.2 | 0.4 | 2.8 | 15.52 | 81.98 | 0.06 | 11.783 |
| 5000 | 195 | 20 | 10 | 65 | 5.2 | 0.4 | 2.8 | 15.52 | 81.98 | 0.06 | 11.783 |
| 6000 | 205 | 20 | 10 | 65 | 5.2 | 0.4 | 2.8 | 15.52 | 81.98 | 0.06 | 11.783 |
| 7000 | 215 | 20 | 10 | 65 | 5.2 | 0.4 | 2.8 | 15.52 | 81.98 | 0.06 | 11.783 |
| 8000 | 225 | 20 | 10 | 65 | 5.2 | 0.4 | 2.8 | 15.52 | 81.98 | 0.06 | 11.783 |
| 9000 | 230 | 20 | 10 | 65 | 5.2 | 0.4 | 2.8 | 15.52 | 81.98 | 0.06 | 11.783 |
| 10000 | 240 | 20 | 10 | 65 | 5.2 | 0.4 | 2.8 | 15.52 | 81.98 | 0.06 | 11.783 |
| 1 | 225 | 20 | 20 | 65 | 4 | 0.4 | 6.66 | 17.25 | 61.36 | 0.06 | 8.737 |
| 2 | 260 | 20 | 20 | 65 | 4 | 0.4 | 6.66 | 17.25 | 61.36 | 0.06 | 8.737 |
| 10 | 350 | 20 | 20 | 65 | 4 | 0.4 | 6.66 | 17.25 | 61.36 | 0.06 | 8.737 |
| 100 | 545 | 20 | 20 | 65 | 4 | 0.4 | 6.66 | 17.25 | 61.36 | 0.06 | 8.737 |
| 500 | 740 | 20 | 20 | 65 | 4 | 0.4 | 6.66 | 17.25 | 61.36 | 0.06 | 8.737 |
| 1000 | 845 | 20 | 20 | 65 | 4 | 0.4 | 6.66 | 17.25 | 61.36 | 0.06 | 8.737 |
| 2000 | 970 | 20 | 20 | 65 | 4 | 0.4 | 6.66 | 17.25 | 61.36 | 0.06 | 8.737 |
| 3000 | 1045 | 20 | 20 | 65 | 4 | 0.4 | 6.66 | 17.25 | 61.36 | 0.06 | 8.737 |
| 4000 | 1100 | 20 | 20 | 65 | 4 | 0.4 | 6.66 | 17.25 | 61.36 | 0.06 | 8.737 |
| 5000 | 1155 | 20 | 20 | 65 | 4 | 0.4 | 6.66 | 17.25 | 61.36 | 0.06 | 8.737 |
| 6000 | 1195 | 20 | 20 | 65 | 4 | 0.4 | 6.66 | 17.25 | 61.36 | 0.06 | 8.737 |
| 7000 | 1235 | 20 | 20 | 65 | 4 | 0.4 | 6.66 | 17.25 | 61.36 | 0.06 | 8.737 |
| 8000 | 1265 | 20 | 20 | 65 | 4 | 0.4 | 6.66 | 17.25 | 61.36 | 0.06 | 8.737 |
| 9000 | 1290 | 20 | 20 | 65 | 4 | 0.4 | 6.66 | 17.25 | 61.36 | 0.06 | 8.737 |
| 10000 | 1320 | 20 | 20 | 65 | 4 | 0.4 | 6.66 | 17.25 | 61.36 | 0.06 | 8.737 |
| 1 | 125 | 20 | 20 | 65 | 4.6 | 0.4 | 4.04 | 15.77 | 74.35 | 0.06 | 10.312 |
| 2 | 145 | 20 | 20 | 65 | 4.6 | 0.4 | 4.04 | 15.77 | 74.35 | 0.06 | 10.312 |
| **N** | **E_p_** | **T** | **S** | **P** | **C** | **D** | **A** | **M** | **F** | **B** | **E** |
| 10 | 215 | 20 | 20 | 65 | 4.6 | 0.4 | 4.04 | 15.77 | 74.35 | 0.06 | 10.312 |
| 100 | 375 | 20 | 20 | 65 | 4.6 | 0.4 | 4.04 | 15.77 | 74.35 | 0.06 | 10.312 |
| 500 | 550 | 20 | 20 | 65 | 4.6 | 0.4 | 4.04 | 15.77 | 74.35 | 0.06 | 10.312 |
| 1000 | 645 | 20 | 20 | 65 | 4.6 | 0.4 | 4.04 | 15.77 | 74.35 | 0.06 | 10.312 |
| 2000 | 765 | 20 | 20 | 65 | 4.6 | 0.4 | 4.04 | 15.77 | 74.35 | 0.06 | 10.312 |
| 3000 | 840 | 20 | 20 | 65 | 4.6 | 0.4 | 4.04 | 15.77 | 74.35 | 0.06 | 10.312 |
| 4000 | 900 | 20 | 20 | 65 | 4.6 | 0.4 | 4.04 | 15.77 | 74.35 | 0.06 | 10.312 |
| 5000 | 950 | 20 | 20 | 65 | 4.6 | 0.4 | 4.04 | 15.77 | 74.35 | 0.06 | 10.312 |
| 6000 | 990 | 20 | 20 | 65 | 4.6 | 0.4 | 4.04 | 15.77 | 74.35 | 0.06 | 10.312 |
| 7000 | 1030 | 20 | 20 | 65 | 4.6 | 0.4 | 4.04 | 15.77 | 74.35 | 0.06 | 10.312 |
| 8000 | 1060 | 20 | 20 | 65 | 4.6 | 0.4 | 4.04 | 15.77 | 74.35 | 0.06 | 10.312 |
| 9000 | 1090 | 20 | 20 | 65 | 4.6 | 0.4 | 4.04 | 15.77 | 74.35 | 0.06 | 10.312 |
| 10000 | 1120 | 20 | 20 | 65 | 4.6 | 0.4 | 4.04 | 15.77 | 74.35 | 0.06 | 10.312 |
| 1 | 100 | 20 | 20 | 65 | 5.2 | 0.4 | 2.99 | 15.69 | 80.97 | 0.06 | 11.78 |
| 2 | 120 | 20 | 20 | 65 | 5.2 | 0.4 | 2.99 | 15.69 | 80.97 | 0.06 | 11.78 |
| 10 | 195 | 20 | 20 | 65 | 5.2 | 0.4 | 2.99 | 15.69 | 80.97 | 0.06 | 11.78 |
| 100 | 370 | 20 | 20 | 65 | 5.2 | 0.4 | 2.99 | 15.69 | 80.97 | 0.06 | 11.78 |
| 500 | 590 | 20 | 20 | 65 | 5.2 | 0.4 | 2.99 | 15.69 | 80.97 | 0.06 | 11.78 |
| 1000 | 715 | 20 | 20 | 65 | 5.2 | 0.4 | 2.99 | 15.69 | 80.97 | 0.06 | 11.78 |
| 2000 | 875 | 20 | 20 | 65 | 5.2 | 0.4 | 2.99 | 15.69 | 80.97 | 0.06 | 11.78 |
| 3000 | 980 | 20 | 20 | 65 | 5.2 | 0.4 | 2.99 | 15.69 | 80.97 | 0.06 | 11.78 |
| 4000 | 1060 | 20 | 20 | 65 | 5.2 | 0.4 | 2.99 | 15.69 | 80.97 | 0.06 | 11.78 |
| 5000 | 1130 | 20 | 20 | 65 | 5.2 | 0.4 | 2.99 | 15.69 | 80.97 | 0.06 | 11.78 |
| 6000 | 1195 | 20 | 20 | 65 | 5.2 | 0.4 | 2.99 | 15.69 | 80.97 | 0.06 | 11.78 |
| 7000 | 1250 | 20 | 20 | 65 | 5.2 | 0.4 | 2.99 | 15.69 | 80.97 | 0.06 | 11.78 |
| 8000 | 1300 | 20 | 20 | 65 | 5.2 | 0.4 | 2.99 | 15.69 | 80.97 | 0.06 | 11.78 |
| 9000 | 1345 | 20 | 20 | 65 | 5.2 | 0.4 | 2.99 | 15.69 | 80.97 | 0.06 | 11.78 |
| 10000 | 1385 | 20 | 20 | 65 | 5.2 | 0.4 | 2.99 | 15.69 | 80.97 | 0.06 | 11.78 |
| 1 | 450 | 20 | 30 | 65 | 4 | 0.4 | 6.55 | 17.15 | 61.79 | 0.06 | 8.748 |
| 2 | 545 | 20 | 30 | 65 | 4 | 0.4 | 6.55 | 17.15 | 61.79 | 0.06 | 8.748 |
| 10 | 855 | 20 | 30 | 65 | 4 | 0.4 | 6.55 | 17.15 | 61.79 | 0.06 | 8.748 |
| 100 | 1630 | 20 | 30 | 65 | 4 | 0.4 | 6.55 | 17.15 | 61.79 | 0.06 | 8.748 |
| 500 | 2550 | 20 | 30 | 65 | 4 | 0.4 | 6.55 | 17.15 | 61.79 | 0.06 | 8.748 |
| 1000 | 3100 | 20 | 30 | 65 | 4 | 0.4 | 6.55 | 17.15 | 61.79 | 0.06 | 8.748 |
| 2000 | 3760 | 20 | 30 | 65 | 4 | 0.4 | 6.55 | 17.15 | 61.79 | 0.06 | 8.748 |
| 3000 | 4215 | 20 | 30 | 65 | 4 | 0.4 | 6.55 | 17.15 | 61.79 | 0.06 | 8.748 |
| 4000 | 4560 | 20 | 30 | 65 | 4 | 0.4 | 6.55 | 17.15 | 61.79 | 0.06 | 8.748 |
| 5000 | 4860 | 20 | 30 | 65 | 4 | 0.4 | 6.55 | 17.15 | 61.79 | 0.06 | 8.748 |
| 6000 | 5115 | 20 | 30 | 65 | 4 | 0.4 | 6.55 | 17.15 | 61.79 | 0.06 | 8.748 |
| 7000 | 5340 | 20 | 30 | 65 | 4 | 0.4 | 6.55 | 17.15 | 61.79 | 0.06 | 8.748 |
| 8000 | 5540 | 20 | 30 | 65 | 4 | 0.4 | 6.55 | 17.15 | 61.79 | 0.06 | 8.748 |
| 9000 | 5730 | 20 | 30 | 65 | 4 | 0.4 | 6.55 | 17.15 | 61.79 | 0.06 | 8.748 |
| 10000 | 5900 | 20 | 30 | 65 | 4 | 0.4 | 6.55 | 17.15 | 61.79 | 0.06 | 8.748 |
| 1 | 335 | 20 | 30 | 65 | 4.6 | 0.4 | 4.15 | 15.86 | 73.82 | 0.06 | 10.03 |
| 2 | 420 | 20 | 30 | 65 | 4.6 | 0.4 | 4.15 | 15.86 | 73.82 | 0.06 | 10.03 |
| 10 | 715 | 20 | 30 | 65 | 4.6 | 0.4 | 4.15 | 15.86 | 73.82 | 0.06 | 10.03 |
| 100 | 1520 | 20 | 30 | 65 | 4.6 | 0.4 | 4.15 | 15.86 | 73.82 | 0.06 | 10.03 |
| 500 | 2580 | 20 | 30 | 65 | 4.6 | 0.4 | 4.15 | 15.86 | 73.82 | 0.06 | 10.03 |
| 1000 | 3250 | 20 | 30 | 65 | 4.6 | 0.4 | 4.15 | 15.86 | 73.82 | 0.06 | 10.03 |
| 2000 | 4080 | 20 | 30 | 65 | 4.6 | 0.4 | 4.15 | 15.86 | 73.82 | 0.06 | 10.03 |
| 3000 | 4660 | 20 | 30 | 65 | 4.6 | 0.4 | 4.15 | 15.86 | 73.82 | 0.06 | 10.03 |
| 4000 | 5120 | 20 | 30 | 65 | 4.6 | 0.4 | 4.15 | 15.86 | 73.82 | 0.06 | 10.03 |
| **N** | **E_p_** | **T** | **S** | **P** | **C** | **D** | **A** | **M** | **F** | **B** | **E** |
| 5000 | 5510 | 20 | 30 | 65 | 4.6 | 0.4 | 4.15 | 15.86 | 73.82 | 0.06 | 10.03 |
| 6000 | 5850 | 20 | 30 | 65 | 4.6 | 0.4 | 4.15 | 15.86 | 73.82 | 0.06 | 10.03 |
| 7000 | 6160 | 20 | 30 | 65 | 4.6 | 0.4 | 4.15 | 15.86 | 73.82 | 0.06 | 10.03 |
| 8000 | 6440 | 20 | 30 | 65 | 4.6 | 0.4 | 4.15 | 15.86 | 73.82 | 0.06 | 10.03 |
| 9000 | 6690 | 20 | 30 | 65 | 4.6 | 0.4 | 4.15 | 15.86 | 73.82 | 0.06 | 10.03 |
| 10000 | 6930 | 20 | 30 | 65 | 4.6 | 0.4 | 4.15 | 15.86 | 73.82 | 0.06 | 10.03 |
| 1 | 410 | 20 | 30 | 65 | 5.2 | 0.4 | 3 | 15.7 | 80.89 | 0.06 | 11.759 |
| 2 | 535 | 20 | 30 | 65 | 5.2 | 0.4 | 3 | 15.7 | 80.89 | 0.06 | 11.759 |
| 10 | 1000 | 20 | 30 | 65 | 5.2 | 0.4 | 3 | 15.7 | 80.89 | 0.06 | 11.759 |
| 100 | 2455 | 20 | 30 | 65 | 5.2 | 0.4 | 3 | 15.7 | 80.89 | 0.06 | 11.759 |
| 500 | 4585 | 20 | 30 | 65 | 5.2 | 0.4 | 3 | 15.7 | 80.89 | 0.06 | 11.759 |
| 1000 | 6000 | 20 | 30 | 65 | 5.2 | 0.4 | 3 | 15.7 | 80.89 | 0.06 | 11.759 |
| 2000 | 7850 | 20 | 30 | 65 | 5.2 | 0.4 | 3 | 15.7 | 80.89 | 0.06 | 11.759 |
| 3000 | 9190 | 20 | 30 | 65 | 5.2 | 0.4 | 3 | 15.7 | 80.89 | 0.06 | 11.759 |
| 4000 | 10280 | 20 | 30 | 65 | 5.2 | 0.4 | 3 | 15.7 | 80.89 | 0.06 | 11.759 |
| 5000 | 1120 | 20 | 30 | 65 | 5.2 | 0.4 | 3 | 15.7 | 80.89 | 0.06 | 11.759 |
| 6000 | 12040 | 20 | 30 | 65 | 5.2 | 0.4 | 3 | 15.7 | 80.89 | 0.06 | 11.759 |
| 7000 | 12780 | 20 | 30 | 65 | 5.2 | 0.4 | 3 | 15.7 | 80.89 | 0.06 | 11.759 |
| 8000 | 13460 | 20 | 30 | 65 | 5.2 | 0.4 | 3 | 15.7 | 80.89 | 0.06 | 11.759 |
| 9000 | 14090 | 20 | 30 | 65 | 5.2 | 0.4 | 3 | 15.7 | 80.89 | 0.06 | 11.759 |
| 10000 | 14680 | 20 | 30 | 65 | 5.2 | 0.4 | 3 | 15.7 | 80.89 | 0.06 | 11.759 |
| 1 | 80 | 40 | 10 | 44 | 4 | 0.1 | 6.75 | 16.64 | 59.46 | 0.061 | 8.689 |
| 2 | 100 | 40 | 10 | 44 | 4 | 0.1 | 6.75 | 16.64 | 59.46 | 0.061 | 8.689 |
| 10 | 180 | 40 | 10 | 44 | 4 | 0.1 | 6.75 | 16.64 | 59.46 | 0.061 | 8.689 |
| 100 | 405 | 40 | 10 | 44 | 4 | 0.1 | 6.75 | 16.64 | 59.46 | 0.061 | 8.689 |
| 500 | 710 | 40 | 10 | 44 | 4 | 0.1 | 6.75 | 16.64 | 59.46 | 0.061 | 8.689 |
| 1000 | 910 | 40 | 10 | 44 | 4 | 0.1 | 6.75 | 16.64 | 59.46 | 0.061 | 8.689 |
| 2000 | 1160 | 40 | 10 | 44 | 4 | 0.1 | 6.75 | 16.64 | 59.46 | 0.061 | 8.689 |
| 3000 | 1330 | 40 | 10 | 44 | 4 | 0.1 | 6.75 | 16.64 | 59.46 | 0.061 | 8.689 |
| 4000 | 1470 | 40 | 10 | 44 | 4 | 0.1 | 6.75 | 16.64 | 59.46 | 0.061 | 8.689 |
| 5000 | 1590 | 40 | 10 | 44 | 4 | 0.1 | 6.75 | 16.64 | 59.46 | 0.061 | 8.689 |
| 6000 | 1700 | 40 | 10 | 44 | 4 | 0.1 | 6.75 | 16.64 | 59.46 | 0.061 | 8.689 |
| 7000 | 1800 | 40 | 10 | 44 | 4 | 0.1 | 6.75 | 16.64 | 59.46 | 0.061 | 8.689 |
| 8000 | 1885 | 40 | 10 | 44 | 4 | 0.1 | 6.75 | 16.64 | 59.46 | 0.061 | 8.689 |
| 9000 | 1965 | 40 | 10 | 44 | 4 | 0.1 | 6.75 | 16.64 | 59.46 | 0.061 | 8.689 |
| 10000 | 2040 | 40 | 10 | 44 | 4 | 0.1 | 6.75 | 16.64 | 59.46 | 0.061 | 8.689 |
| 1 | 120 | 40 | 10 | 44 | 4.6 | 0.1 | 4.19 | 15.48 | 72.91 | 0.061 | 10.216 |
| 2 | 155 | 40 | 10 | 44 | 4.6 | 0.1 | 4.19 | 15.48 | 72.91 | 0.061 | 10.216 |
| 10 | 285 | 40 | 10 | 44 | 4.6 | 0.1 | 4.19 | 15.48 | 72.91 | 0.061 | 10.216 |
| 100 | 680 | 40 | 10 | 44 | 4.6 | 0.1 | 4.19 | 15.48 | 72.91 | 0.061 | 10.216 |
| 500 | 1240 | 40 | 10 | 44 | 4.6 | 0.1 | 4.19 | 15.48 | 72.91 | 0.061 | 10.216 |
| 1000 | 1610 | 40 | 10 | 44 | 4.6 | 0.1 | 4.19 | 15.48 | 72.91 | 0.061 | 10.216 |
| 2000 | 2090 | 40 | 10 | 44 | 4.6 | 0.1 | 4.19 | 15.48 | 72.91 | 0.061 | 10.216 |
| 3000 | 2430 | 40 | 10 | 44 | 4.6 | 0.1 | 4.19 | 15.48 | 72.91 | 0.061 | 10.216 |
| 4000 | 2710 | 40 | 10 | 44 | 4.6 | 0.1 | 4.19 | 15.48 | 72.91 | 0.061 | 10.216 |
| 5000 | 2950 | 40 | 10 | 44 | 4.6 | 0.1 | 4.19 | 15.48 | 72.91 | 0.061 | 10.216 |
| 6000 | 3160 | 40 | 10 | 44 | 4.6 | 0.1 | 4.19 | 15.48 | 72.91 | 0.061 | 10.216 |
| 7000 | 3350 | 40 | 10 | 44 | 4.6 | 0.1 | 4.19 | 15.48 | 72.91 | 0.061 | 10.216 |
| 8000 | 3520 | 40 | 10 | 44 | 4.6 | 0.1 | 4.19 | 15.48 | 72.91 | 0.061 | 10.216 |
| 9000 | 3680 | 40 | 10 | 44 | 4.6 | 0.1 | 4.19 | 15.48 | 72.91 | 0.061 | 10.216 |
| 10000 | 3830 | 40 | 10 | 44 | 4.6 | 0.1 | 4.19 | 15.48 | 72.91 | 0.061 | 10.216 |
| 1 | 30 | 40 | 10 | 44 | 5.2 | 0.1 | 3.22 | 15.44 | 79.15 | 0.061 | 11.646 |
| **N** | **E_p_** | **T** | **S** | **P** | **C** | **D** | **A** | **M** | **F** | **B** | **E** |
| 2 | 40 | 40 | 10 | 44 | 5.2 | 0.1 | 3.22 | 15.44 | 79.15 | 0.061 | 11.646 |
| 10 | 75 | 40 | 10 | 44 | 5.2 | 0.1 | 3.22 | 15.44 | 79.15 | 0.061 | 11.646 |
| 100 | 185 | 40 | 10 | 44 | 5.2 | 0.1 | 3.22 | 15.44 | 79.15 | 0.061 | 11.646 |
| 500 | 350 | 40 | 10 | 44 | 5.2 | 0.1 | 3.22 | 15.44 | 79.15 | 0.061 | 11.646 |
| 1000 | 460 | 40 | 10 | 44 | 5.2 | 0.1 | 3.22 | 15.44 | 79.15 | 0.061 | 11.646 |
| 2000 | 605 | 40 | 10 | 44 | 5.2 | 0.1 | 3.22 | 15.44 | 79.15 | 0.061 | 11.646 |
| 3000 | 710 | 40 | 10 | 44 | 5.2 | 0.1 | 3.22 | 15.44 | 79.15 | 0.061 | 11.646 |
| 4000 | 790 | 40 | 10 | 44 | 5.2 | 0.1 | 3.22 | 15.44 | 79.15 | 0.061 | 11.646 |
| 5000 | 870 | 40 | 10 | 44 | 5.2 | 0.1 | 3.22 | 15.44 | 79.15 | 0.061 | 11.646 |
| 6000 | 930 | 40 | 10 | 44 | 5.2 | 0.1 | 3.22 | 15.44 | 79.15 | 0.061 | 11.646 |
| 7000 | 990 | 40 | 10 | 44 | 5.2 | 0.1 | 3.22 | 15.44 | 79.15 | 0.061 | 11.646 |
| 8000 | 1050 | 40 | 10 | 44 | 5.2 | 0.1 | 3.22 | 15.44 | 79.15 | 0.061 | 11.646 |
| 9000 | 1100 | 40 | 10 | 44 | 5.2 | 0.1 | 3.22 | 15.44 | 79.15 | 0.061 | 11.646 |
| 10000 | 1140 | 40 | 10 | 44 | 5.2 | 0.1 | 3.22 | 15.44 | 79.15 | 0.061 | 11.646 |
| 1 | 130 | 40 | 20 | 44 | 4 | 0.1 | 6.75 | 16.64 | 59.46 | 0.061 | 8.689 |
| 2 | 165 | 40 | 20 | 44 | 4 | 0.1 | 6.75 | 16.64 | 59.46 | 0.061 | 8.689 |
| 10 | 300 | 40 | 20 | 44 | 4 | 0.1 | 6.75 | 16.64 | 59.46 | 0.061 | 8.689 |
| 100 | 700 | 40 | 20 | 44 | 4 | 0.1 | 6.75 | 16.64 | 59.46 | 0.061 | 8.689 |
| 500 | 1240 | 40 | 20 | 44 | 4 | 0.1 | 6.75 | 16.64 | 59.46 | 0.061 | 8.689 |
| 1000 | 1600 | 40 | 20 | 44 | 4 | 0.1 | 6.75 | 16.64 | 59.46 | 0.061 | 8.689 |
| 2000 | 2050 | 40 | 20 | 44 | 4 | 0.1 | 6.75 | 16.64 | 59.46 | 0.061 | 8.689 |
| 3000 | 2370 | 40 | 20 | 44 | 4 | 0.1 | 6.75 | 16.64 | 59.46 | 0.061 | 8.689 |
| 4000 | 2630 | 40 | 20 | 44 | 4 | 0.1 | 6.75 | 16.64 | 59.46 | 0.061 | 8.689 |
| 5000 | 2850 | 40 | 20 | 44 | 4 | 0.1 | 6.75 | 16.64 | 59.46 | 0.061 | 8.689 |
| 6000 | 1320 | 40 | 20 | 44 | 4 | 0.1 | 6.75 | 16.64 | 59.46 | 0.061 | 8.689 |
| 7000 | 3220 | 40 | 20 | 44 | 4 | 0.1 | 6.75 | 16.64 | 59.46 | 0.061 | 8.689 |
| 8000 | 3380 | 40 | 20 | 44 | 4 | 0.1 | 6.75 | 16.64 | 59.46 | 0.061 | 8.689 |
| 8500 | 3457 | 40 | 20 | 44 | 4 | 0.1 | 6.75 | 16.64 | 59.46 | 0.061 | 8.689 |
| 9000 | 3530 | 40 | 20 | 44 | 4 | 0.1 | 6.75 | 16.64 | 59.46 | 0.061 | 8.689 |
| 9250 | 3700 | 40 | 20 | 44 | 4 | 0.1 | 6.75 | 16.64 | 59.46 | 0.061 | 8.689 |
| 1 | 70 | 40 | 20 | 44 | 4.6 | 0.1 | 4.15 | 15.44 | 73.15 | 0.061 | 10.221 |
| 2 | 90 | 40 | 20 | 44 | 4.6 | 0.1 | 4.15 | 15.44 | 73.15 | 0.061 | 10.221 |
| 10 | 180 | 40 | 20 | 44 | 4.6 | 0.1 | 4.15 | 15.44 | 73.15 | 0.061 | 10.221 |
| 100 | 450 | 40 | 20 | 44 | 4.6 | 0.1 | 4.15 | 15.44 | 73.15 | 0.061 | 10.221 |
| 500 | 870 | 40 | 20 | 44 | 4.6 | 0.1 | 4.15 | 15.44 | 73.15 | 0.061 | 10.221 |
| 1000 | 1150 | 40 | 20 | 44 | 4.6 | 0.1 | 4.15 | 15.44 | 73.15 | 0.061 | 10.221 |
| 2000 | 1520 | 40 | 20 | 44 | 4.6 | 0.1 | 4.15 | 15.44 | 73.15 | 0.061 | 10.221 |
| 3000 | 1800 | 40 | 20 | 44 | 4.6 | 0.1 | 4.15 | 15.44 | 73.15 | 0.061 | 10.221 |
| 4000 | 2020 | 40 | 20 | 44 | 4.6 | 0.1 | 4.15 | 15.44 | 73.15 | 0.061 | 10.221 |
| 5000 | 2210 | 40 | 20 | 44 | 4.6 | 0.1 | 4.15 | 15.44 | 73.15 | 0.061 | 10.221 |
| 6000 | 2380 | 40 | 20 | 44 | 4.6 | 0.1 | 4.15 | 15.44 | 73.15 | 0.061 | 10.221 |
| 7000 | 2540 | 40 | 20 | 44 | 4.6 | 0.1 | 4.15 | 15.44 | 73.15 | 0.061 | 10.221 |
| 8000 | 2680 | 40 | 20 | 44 | 4.6 | 0.1 | 4.15 | 15.44 | 73.15 | 0.061 | 10.221 |
| 8500 | 2860 | 40 | 20 | 44 | 4.6 | 0.1 | 4.15 | 15.44 | 73.15 | 0.061 | 10.221 |
| 1 | 95 | 40 | 20 | 44 | 5.2 | 0.1 | 3.19 | 15.41 | 79.3 | 0.061 | 11.649 |
| 2 | 130 | 40 | 20 | 44 | 5.2 | 0.1 | 3.19 | 15.41 | 79.3 | 0.061 | 11.649 |
| 10 | 260 | 40 | 20 | 44 | 5.2 | 0.1 | 3.19 | 15.41 | 79.3 | 0.061 | 11.649 |
| 100 | 710 | 40 | 20 | 44 | 5.2 | 0.1 | 3.19 | 15.41 | 79.3 | 0.061 | 11.649 |
| 500 | 1430 | 40 | 20 | 44 | 5.2 | 0.1 | 3.19 | 15.41 | 79.3 | 0.061 | 11.649 |
| 1000 | 1940 | 40 | 20 | 44 | 5.2 | 0.1 | 3.19 | 15.41 | 79.3 | 0.061 | 11.649 |
| 2000 | 2630 | 40 | 20 | 44 | 5.2 | 0.1 | 3.19 | 15.41 | 79.3 | 0.061 | 11.649 |
| 3000 | 3130 | 40 | 20 | 44 | 5.2 | 0.1 | 3.19 | 15.41 | 79.3 | 0.061 | 11.649 |
| **N** | **E_p_** | **T** | **S** | **P** | **C** | **D** | **A** | **M** | **F** | **B** | **E** |
| 4000 | 3550 | 40 | 20 | 44 | 5.2 | 0.1 | 3.19 | 15.41 | 79.3 | 0.061 | 11.649 |
| 5000 | 3920 | 40 | 20 | 44 | 5.2 | 0.1 | 3.19 | 15.41 | 79.3 | 0.061 | 11.649 |
| 6000 | 4240 | 40 | 20 | 44 | 5.2 | 0.1 | 3.19 | 15.41 | 79.3 | 0.061 | 11.649 |
| 7000 | 4540 | 40 | 20 | 44 | 5.2 | 0.1 | 3.19 | 15.41 | 79.3 | 0.061 | 11.649 |
| 8000 | 4930 | 40 | 20 | 44 | 5.2 | 0.1 | 3.19 | 15.41 | 79.3 | 0.061 | 11.649 |
| 1 | 280 | 40 | 30 | 44 | 4 | 0.1 | 6.53 | 16.45 | 60.31 | 0.061 | 8.709 |
| 2 | 360 | 40 | 30 | 44 | 4 | 0.1 | 6.53 | 16.45 | 60.31 | 0.061 | 8.709 |
| 10 | 660 | 40 | 30 | 44 | 4 | 0.1 | 6.53 | 16.45 | 60.31 | 0.061 | 8.709 |
| 100 | 1540 | 40 | 30 | 44 | 4 | 0.1 | 6.53 | 16.45 | 60.31 | 0.061 | 8.709 |
| 500 | 2800 | 40 | 30 | 44 | 4 | 0.1 | 6.53 | 16.45 | 60.31 | 0.061 | 8.709 |
| 1000 | 3620 | 40 | 30 | 44 | 4 | 0.1 | 6.53 | 16.45 | 60.31 | 0.061 | 8.709 |
| 2000 | 4680 | 40 | 30 | 44 | 4 | 0.1 | 6.53 | 16.45 | 60.31 | 0.061 | 8.709 |
| 3000 | 5440 | 40 | 30 | 44 | 4 | 0.1 | 6.53 | 16.45 | 60.31 | 0.061 | 8.709 |
| 4000 | 6050 | 40 | 30 | 44 | 4 | 0.1 | 6.53 | 16.45 | 60.31 | 0.061 | 8.709 |
| 5000 | 6570 | 40 | 30 | 44 | 4 | 0.1 | 6.53 | 16.45 | 60.31 | 0.061 | 8.709 |
| 5500 | 6810 | 40 | 30 | 44 | 4 | 0.1 | 6.53 | 16.45 | 60.31 | 0.061 | 8.709 |
| 5900 | 7350 | 40 | 30 | 44 | 4 | 0.1 | 6.53 | 16.45 | 60.31 | 0.061 | 8.709 |
| 1 | 180 | 40 | 30 | 44 | 4.6 | 0.1 | 4.17 | 15.46 | 73.03 | 0.061 | 10.218 |
| 2 | 240 | 40 | 30 | 44 | 4.6 | 0.1 | 4.17 | 15.46 | 73.03 | 0.061 | 10.218 |
| 10 | 490 | 40 | 30 | 44 | 4.6 | 0.1 | 4.17 | 15.46 | 73.03 | 0.061 | 10.218 |
| 100 | 1330 | 40 | 30 | 44 | 4.6 | 0.1 | 4.17 | 15.46 | 73.03 | 0.061 | 10.218 |
| 500 | 2660 | 40 | 30 | 44 | 4.6 | 0.1 | 4.17 | 15.46 | 73.03 | 0.061 | 10.218 |
| 1000 | 3600 | 40 | 30 | 44 | 4.6 | 0.1 | 4.17 | 15.46 | 73.03 | 0.061 | 10.218 |
| 2000 | 4870 | 40 | 30 | 44 | 4.6 | 0.1 | 4.17 | 15.46 | 73.03 | 0.061 | 10.218 |
| 3000 | 5800 | 40 | 30 | 44 | 4.6 | 0.1 | 4.17 | 15.46 | 73.03 | 0.061 | 10.218 |
| 4000 | 6570 | 40 | 30 | 44 | 4.6 | 0.1 | 4.17 | 15.46 | 73.03 | 0.061 | 10.218 |
| 4500 | 6900 | 40 | 30 | 44 | 4.6 | 0.1 | 4.17 | 15.46 | 73.03 | 0.061 | 10.218 |
| 5000 | 7520 | 40 | 30 | 44 | 4.6 | 0.1 | 4.17 | 15.46 | 73.03 | 0.061 | 10.218 |
| 1 | 110 | 40 | 30 | 44 | 5.2 | 0.1 | 3.19 | 15.41 | 79.3 | 0.061 | 11.649 |
| 2 | 150 | 40 | 30 | 44 | 5.2 | 0.1 | 3.19 | 15.41 | 79.3 | 0.061 | 11.649 |
| 10 | 310 | 40 | 30 | 44 | 5.2 | 0.1 | 3.19 | 15.41 | 79.3 | 0.061 | 11.649 |
| 100 | 910 | 40 | 30 | 44 | 5.2 | 0.1 | 3.19 | 15.41 | 79.3 | 0.061 | 11.649 |
| 500 | 1900 | 40 | 30 | 44 | 5.2 | 0.1 | 3.19 | 15.41 | 79.3 | 0.061 | 11.649 |
| 1000 | 2610 | 40 | 30 | 44 | 5.2 | 0.1 | 3.19 | 15.41 | 79.3 | 0.061 | 11.649 |
| 2000 | 3600 | 40 | 30 | 44 | 5.2 | 0.1 | 3.19 | 15.41 | 79.3 | 0.061 | 11.649 |
| 3000 | 4330 | 40 | 30 | 44 | 5.2 | 0.1 | 3.19 | 15.41 | 79.3 | 0.061 | 11.649 |
| 4000 | 4940 | 40 | 30 | 44 | 5.2 | 0.1 | 3.19 | 15.41 | 79.3 | 0.061 | 11.649 |
| 4500 | 5220 | 40 | 30 | 44 | 5.2 | 0.1 | 3.19 | 15.41 | 79.3 | 0.061 | 11.649 |
| 4650 | 6100 | 40 | 30 | 44 | 5.2 | 0.1 | 3.19 | 15.41 | 79.3 | 0.061 | 11.649 |
| 1 | 150 | 40 | 10 | 44 | 4 | 0.4 | 6.77 | 16.66 | 59.36 | 0.061 | 8.687 |
| 2 | 200 | 40 | 10 | 44 | 4 | 0.4 | 6.77 | 16.66 | 59.36 | 0.061 | 8.687 |
| 10 | 370 | 40 | 10 | 44 | 4 | 0.4 | 6.77 | 16.66 | 59.36 | 0.061 | 8.687 |
| 100 | 920 | 40 | 10 | 44 | 4 | 0.4 | 6.77 | 16.66 | 59.36 | 0.061 | 8.687 |
| 500 | 1710 | 40 | 10 | 44 | 4 | 0.4 | 6.77 | 16.66 | 59.36 | 0.061 | 8.687 |
| 1000 | 2250 | 40 | 10 | 44 | 4 | 0.4 | 6.77 | 16.66 | 59.36 | 0.061 | 8.687 |
| 2000 | 2960 | 40 | 10 | 44 | 4 | 0.4 | 6.77 | 16.66 | 59.36 | 0.061 | 8.687 |
| 3000 | 3460 | 40 | 10 | 44 | 4 | 0.4 | 6.77 | 16.66 | 59.36 | 0.061 | 8.687 |
| 4000 | 3880 | 40 | 10 | 44 | 4 | 0.4 | 6.77 | 16.66 | 59.36 | 0.061 | 8.687 |
| 4250 | 3970 | 40 | 10 | 44 | 4 | 0.4 | 6.77 | 16.66 | 59.36 | 0.061 | 8.687 |
| 4500 | 4060 | 40 | 10 | 44 | 4 | 0.4 | 6.77 | 16.66 | 59.36 | 0.061 | 8.687 |
| 4600 | 4350 | 40 | 10 | 44 | 4 | 0.4 | 6.77 | 16.66 | 59.36 | 0.061 | 8.687 |
| 1 | 230 | 40 | 10 | 44 | 4.6 | 0.4 | 4.51 | 15.76 | 71.37 | 0.061 | 10.182 |
| **N** | **E_p_** | **T** | **S** | **P** | **C** | **D** | **A** | **M** | **F** | **B** | **E** |
| 2 | 305 | 40 | 10 | 44 | 4.6 | 0.4 | 4.51 | 15.76 | 71.37 | 0.061 | 10.182 |
| 10 | 600 | 40 | 10 | 44 | 4.6 | 0.4 | 4.51 | 15.76 | 71.37 | 0.061 | 10.182 |
| 100 | 1560 | 40 | 10 | 44 | 4.6 | 0.4 | 4.51 | 15.76 | 71.37 | 0.061 | 10.182 |
| 500 | 3050 | 40 | 10 | 44 | 4.6 | 0.4 | 4.51 | 15.76 | 71.37 | 0.061 | 10.182 |
| 1000 | 4060 | 40 | 10 | 44 | 4.6 | 0.4 | 4.51 | 15.76 | 71.37 | 0.061 | 10.182 |
| 2000 | 5420 | 40 | 10 | 44 | 4.6 | 0.4 | 4.51 | 15.76 | 71.37 | 0.061 | 10.182 |
| 3000 | 6410 | 40 | 10 | 44 | 4.6 | 0.4 | 4.51 | 15.76 | 71.37 | 0.061 | 10.182 |
| 4000 | 7230 | 40 | 10 | 44 | 4.6 | 0.4 | 4.51 | 15.76 | 71.37 | 0.061 | 10.182 |
| 4250 | 7410 | 40 | 10 | 44 | 4.6 | 0.4 | 4.51 | 15.76 | 71.37 | 0.061 | 10.182 |
| 4500 | 7590 | 40 | 10 | 44 | 4.6 | 0.4 | 4.51 | 15.76 | 71.37 | 0.061 | 10.182 |
| 4580 | 7750 | 40 | 10 | 44 | 4.6 | 0.4 | 4.51 | 15.76 | 71.37 | 0.061 | 10.182 |
| 1 | 60 | 40 | 10 | 44 | 5.2 | 0.4 | 2.95 | 15.2 | 80.58 | 0.061 | 11.678 |
| 2 | 80 | 40 | 10 | 44 | 5.2 | 0.4 | 2.95 | 15.2 | 80.58 | 0.061 | 11.678 |
| 10 | 160 | 40 | 10 | 44 | 5.2 | 0.4 | 2.95 | 15.2 | 80.58 | 0.061 | 11.678 |
| 100 | 440 | 40 | 10 | 44 | 5.2 | 0.4 | 2.95 | 15.2 | 80.58 | 0.061 | 11.678 |
| 500 | 890 | 40 | 10 | 44 | 5.2 | 0.4 | 2.95 | 15.2 | 80.58 | 0.061 | 11.678 |
| 1000 | 1210 | 40 | 10 | 44 | 5.2 | 0.4 | 2.95 | 15.2 | 80.58 | 0.061 | 11.678 |
| 2000 | 1630 | 40 | 10 | 44 | 5.2 | 0.4 | 2.95 | 15.2 | 80.58 | 0.061 | 11.678 |
| 3000 | 1950 | 40 | 10 | 44 | 5.2 | 0.4 | 2.95 | 15.2 | 80.58 | 0.061 | 11.678 |
| 4000 | 2210 | 40 | 10 | 44 | 5.2 | 0.4 | 2.95 | 15.2 | 80.58 | 0.061 | 11.678 |
| 4250 | 2270 | 40 | 10 | 44 | 5.2 | 0.4 | 2.95 | 15.2 | 80.58 | 0.061 | 11.678 |
| 4500 | 2330 | 40 | 10 | 44 | 5.2 | 0.4 | 2.95 | 15.2 | 80.58 | 0.061 | 11.678 |
| 4550 | 2450 | 40 | 10 | 44 | 5.2 | 0.4 | 2.95 | 15.2 | 80.58 | 0.061 | 11.678 |
| 1 | 300 | 40 | 20 | 44 | 4 | 0.4 | 6.73 | 16.63 | 59.51 | 0.061 | 8.69 |
| 2 | 400 | 40 | 20 | 44 | 4 | 0.4 | 6.73 | 16.63 | 59.51 | 0.061 | 8.69 |
| 10 | 770 | 40 | 20 | 44 | 4 | 0.4 | 6.73 | 16.63 | 59.51 | 0.061 | 8.69 |
| 100 | 1990 | 40 | 20 | 44 | 4 | 0.4 | 6.73 | 16.63 | 59.51 | 0.061 | 8.69 |
| 500 | 3870 | 40 | 20 | 44 | 4 | 0.4 | 6.73 | 16.63 | 59.51 | 0.061 | 8.69 |
| 1000 | 5140 | 40 | 20 | 44 | 4 | 0.4 | 6.73 | 16.63 | 59.51 | 0.061 | 8.69 |
| 2000 | 6840 | 40 | 20 | 44 | 4 | 0.4 | 6.73 | 16.63 | 59.51 | 0.061 | 8.69 |
| 3000 | 8070 | 40 | 20 | 44 | 4 | 0.4 | 6.73 | 16.63 | 59.51 | 0.061 | 8.69 |
| 4000 | 9090 | 40 | 20 | 44 | 4 | 0.4 | 6.73 | 16.63 | 59.51 | 0.061 | 8.69 |
| 4500 | 9540 | 40 | 20 | 44 | 4 | 0.4 | 6.73 | 16.63 | 59.51 | 0.061 | 8.69 |
| 4625 | 10700 | 40 | 20 | 44 | 4 | 0.4 | 6.73 | 16.63 | 59.51 | 0.061 | 8.69 |
| 1 | 150 | 40 | 20 | 44 | 4.6 | 0.4 | 4.07 | 15.38 | 73.51 | 0.061 | 10.229 |
| 2 | 210 | 40 | 20 | 44 | 4.6 | 0.4 | 4.07 | 15.38 | 73.51 | 0.061 | 10.229 |
| 10 | 430 | 40 | 20 | 44 | 4.6 | 0.4 | 4.07 | 15.38 | 73.51 | 0.061 | 10.229 |
| 100 | 1220 | 40 | 20 | 44 | 4.6 | 0.4 | 4.07 | 15.38 | 73.51 | 0.061 | 10.229 |
| 500 | 2540 | 40 | 20 | 44 | 4.6 | 0.4 | 4.07 | 15.38 | 73.51 | 0.061 | 10.229 |
| 1000 | 3480 | 40 | 20 | 44 | 4.6 | 0.4 | 4.07 | 15.38 | 73.51 | 0.061 | 10.229 |
| 2000 | 4770 | 40 | 20 | 44 | 4.6 | 0.4 | 4.07 | 15.38 | 73.51 | 0.061 | 10.229 |
| 3000 | 5740 | 40 | 20 | 44 | 4.6 | 0.4 | 4.07 | 15.38 | 73.51 | 0.061 | 10.229 |
| 4000 | 6540 | 40 | 20 | 44 | 4.6 | 0.4 | 4.07 | 15.38 | 73.51 | 0.061 | 10.229 |
| 4500 | 6900 | 40 | 20 | 44 | 4.6 | 0.4 | 4.07 | 15.38 | 73.51 | 0.061 | 10.229 |
| 4550 | 7400 | 40 | 20 | 44 | 4.6 | 0.4 | 4.07 | 15.38 | 73.51 | 0.061 | 10.229 |
| 1 | 220 | 40 | 20 | 44 | 5.2 | 0.4 | 3.19 | 15.41 | 79.3 | 0.061 | 11.649 |
| 2 | 310 | 40 | 20 | 44 | 5.2 | 0.4 | 3.19 | 15.41 | 79.3 | 0.061 | 11.649 |
| 10 | 670 | 40 | 20 | 44 | 5.2 | 0.4 | 3.19 | 15.41 | 79.3 | 0.061 | 11.649 |
| 100 | 2060 | 40 | 20 | 44 | 5.2 | 0.4 | 3.19 | 15.41 | 79.3 | 0.061 | 11.649 |
| 500 | 4490 | 40 | 20 | 44 | 5.2 | 0.4 | 3.19 | 15.41 | 79.3 | 0.061 | 11.649 |
| 1000 | 6300 | 40 | 20 | 44 | 5.2 | 0.4 | 3.19 | 15.41 | 79.3 | 0.061 | 11.649 |
| 2000 | 8810 | 40 | 20 | 44 | 5.2 | 0.4 | 3.19 | 15.41 | 79.3 | 0.061 | 11.649 |
| **N** | **E_p_** | **T** | **S** | **P** | **C** | **D** | **A** | **M** | **F** | **B** | **E** |
| 3000 | 10730 | 40 | 20 | 44 | 5.2 | 0.4 | 3.19 | 15.41 | 79.3 | 0.061 | 11.649 |
| 4000 | 12330 | 40 | 20 | 44 | 5.2 | 0.4 | 3.19 | 15.41 | 79.3 | 0.061 | 11.649 |
| 4200 | 13230 | 40 | 20 | 44 | 5.2 | 0.4 | 3.19 | 15.41 | 79.3 | 0.061 | 11.649 |
| 1 | 465 | 40 | 30 | 44 | 4 | 0.4 | 6.5 | 16.42 | 60.41 | 0.061 | 8.712 |
| 2 | 630 | 40 | 30 | 44 | 4 | 0.4 | 6.5 | 16.42 | 60.41 | 0.061 | 8.712 |
| 10 | 1270 | 40 | 30 | 44 | 4 | 0.4 | 6.5 | 16.42 | 60.41 | 0.061 | 8.712 |
| 100 | 3500 | 40 | 30 | 44 | 4 | 0.4 | 6.5 | 16.42 | 60.41 | 0.061 | 8.712 |
| 500 | 7100 | 40 | 30 | 44 | 4 | 0.4 | 6.5 | 16.42 | 60.41 | 0.061 | 8.712 |
| 1000 | 9630 | 40 | 30 | 44 | 4 | 0.4 | 6.5 | 16.42 | 60.41 | 0.061 | 8.712 |
| 1250 | 10620 | 40 | 30 | 44 | 4 | 0.4 | 6.5 | 16.42 | 60.41 | 0.061 | 8.712 |
| 1500 | 11500 | 40 | 30 | 44 | 4 | 0.4 | 6.5 | 16.42 | 60.41 | 0.061 | 8.712 |
| 1750 | 12300 | 40 | 30 | 44 | 4 | 0.4 | 6.5 | 16.42 | 60.41 | 0.061 | 8.712 |
| 1950 | 13260 | 40 | 30 | 44 | 4 | 0.4 | 6.5 | 16.42 | 60.41 | 0.061 | 8.712 |
| 1 | 290 | 40 | 30 | 44 | 4.6 | 0.4 | 4.22 | 15.5 | 72.79 | 0.061 | 10.213 |
| 2 | 410 | 40 | 30 | 44 | 4.6 | 0.4 | 4.22 | 15.5 | 72.79 | 0.061 | 10.213 |
| 10 | 920 | 40 | 30 | 44 | 4.6 | 0.4 | 4.22 | 15.5 | 72.79 | 0.061 | 10.213 |
| 100 | 2930 | 40 | 30 | 44 | 4.6 | 0.4 | 4.22 | 15.5 | 72.79 | 0.061 | 10.213 |
| 500 | 6590 | 40 | 30 | 44 | 4.6 | 0.4 | 4.22 | 15.5 | 72.79 | 0.061 | 10.213 |
| 1000 | 9340 | 40 | 30 | 44 | 4.6 | 0.4 | 4.22 | 15.5 | 72.79 | 0.061 | 10.213 |
| 1250 | 10450 | 40 | 30 | 44 | 4.6 | 0.4 | 4.22 | 15.5 | 72.79 | 0.061 | 10.213 |
| 1500 | 11460 | 40 | 30 | 44 | 4.6 | 0.4 | 4.22 | 15.5 | 72.79 | 0.061 | 10.213 |
| 1750 | 12380 | 40 | 30 | 44 | 4.6 | 0.4 | 4.22 | 15.5 | 72.79 | 0.061 | 10.213 |
| 1800 | 12550 | 40 | 30 | 44 | 4.6 | 0.4 | 4.22 | 15.5 | 72.79 | 0.061 | 10.213 |
| 1850 | 12730 | 40 | 30 | 44 | 4.6 | 0.4 | 4.22 | 15.5 | 72.79 | 0.061 | 10.213 |
| 1900 | 12900 | 40 | 30 | 44 | 4.6 | 0.4 | 4.22 | 15.5 | 72.79 | 0.061 | 10.213 |
| 1925 | 13250 | 40 | 30 | 44 | 4.6 | 0.4 | 4.22 | 15.5 | 72.79 | 0.061 | 10.213 |
| 1 | 180 | 40 | 30 | 44 | 5.2 | 0.4 | 2.84 | 15.11 | 81.18 | 0.061 | 11.691 |
| 2 | 260 | 40 | 30 | 44 | 5.2 | 0.4 | 2.84 | 15.11 | 81.18 | 0.061 | 11.691 |
| 10 | 610 | 40 | 30 | 44 | 5.2 | 0.4 | 2.84 | 15.11 | 81.18 | 0.061 | 11.691 |
| 100 | 2080 | 40 | 30 | 44 | 5.2 | 0.4 | 2.84 | 15.11 | 81.18 | 0.061 | 11.691 |
| 500 | 4890 | 40 | 30 | 44 | 5.2 | 0.4 | 2.84 | 15.11 | 81.18 | 0.061 | 11.691 |
| 1000 | 7070 | 40 | 30 | 44 | 5.2 | 0.4 | 2.84 | 15.11 | 81.18 | 0.061 | 11.691 |
| 1250 | 7960 | 40 | 30 | 44 | 5.2 | 0.4 | 2.84 | 15.11 | 81.18 | 0.061 | 11.691 |
| 1500 | 8770 | 40 | 30 | 44 | 5.2 | 0.4 | 2.84 | 15.11 | 81.18 | 0.061 | 11.691 |
| 1750 | 9520 | 40 | 30 | 44 | 5.2 | 0.4 | 2.84 | 15.11 | 81.18 | 0.061 | 11.691 |
| 1775 | 9850 | 40 | 30 | 44 | 5.2 | 0.4 | 2.84 | 15.11 | 81.18 | 0.061 | 11.691 |
| 1 | 110 | 40 | 10 | 65 | 4 | 0.1 | 6.16 | 16.8 | 63.33 | 0.06 | 8.785 |
| 2 | 140 | 40 | 10 | 65 | 4 | 0.1 | 6.16 | 16.8 | 63.33 | 0.06 | 8.785 |
| 10 | 250 | 40 | 10 | 65 | 4 | 0.1 | 6.16 | 16.8 | 63.33 | 0.06 | 8.785 |
| 100 | 560 | 40 | 10 | 65 | 4 | 0.1 | 6.16 | 16.8 | 63.33 | 0.06 | 8.785 |
| 500 | 990 | 40 | 10 | 65 | 4 | 0.1 | 6.16 | 16.8 | 63.33 | 0.06 | 8.785 |
| 1000 | 1270 | 40 | 10 | 65 | 4 | 0.1 | 6.16 | 16.8 | 63.33 | 0.06 | 8.785 |
| 2000 | 1620 | 40 | 10 | 65 | 4 | 0.1 | 6.16 | 16.8 | 63.33 | 0.06 | 8.785 |
| 3000 | 1870 | 40 | 10 | 65 | 4 | 0.1 | 6.16 | 16.8 | 63.33 | 0.06 | 8.785 |
| 4000 | 2070 | 40 | 10 | 65 | 4 | 0.1 | 6.16 | 16.8 | 63.33 | 0.06 | 8.785 |
| 5000 | 2240 | 40 | 10 | 65 | 4 | 0.1 | 6.16 | 16.8 | 63.33 | 0.06 | 8.785 |
| 6000 | 2390 | 40 | 10 | 65 | 4 | 0.1 | 6.16 | 16.8 | 63.33 | 0.06 | 8.785 |
| 7000 | 2520 | 40 | 10 | 65 | 4 | 0.1 | 6.16 | 16.8 | 63.33 | 0.06 | 8.785 |
| 8000 | 2650 | 40 | 10 | 65 | 4 | 0.1 | 6.16 | 16.8 | 63.33 | 0.06 | 8.785 |
| 9000 | 2760 | 40 | 10 | 65 | 4 | 0.1 | 6.16 | 16.8 | 63.33 | 0.06 | 8.785 |
| 10000 | 2860 | 40 | 10 | 65 | 4 | 0.1 | 6.16 | 16.8 | 63.33 | 0.06 | 8.785 |
| 1 | 65 | 40 | 10 | 65 | 4.6 | 0.1 | 4.31 | 16 | 73.06 | 0.06 | 10.284 |
| **N** | **E_p_** | **T** | **S** | **P** | **C** | **D** | **A** | **M** | **F** | **B** | **E** |
| 2 | 85 | 40 | 10 | 65 | 4.6 | 0.1 | 4.31 | 16 | 73.06 | 0.06 | 10.284 |
| 10 | 155 | 40 | 10 | 65 | 4.6 | 0.1 | 4.31 | 16 | 73.06 | 0.06 | 10.284 |
| 100 | 370 | 40 | 10 | 65 | 4.6 | 0.1 | 4.31 | 16 | 73.06 | 0.06 | 10.284 |
| 500 | 680 | 40 | 10 | 65 | 4.6 | 0.1 | 4.31 | 16 | 73.06 | 0.06 | 10.284 |
| 1000 | 890 | 40 | 10 | 65 | 4.6 | 0.1 | 4.31 | 16 | 73.06 | 0.06 | 10.284 |
| 2000 | 1155 | 40 | 10 | 65 | 4.6 | 0.1 | 4.31 | 16 | 73.06 | 0.06 | 10.284 |
| 3000 | 1345 | 40 | 10 | 65 | 4.6 | 0.1 | 4.31 | 16 | 73.06 | 0.06 | 10.284 |
| 4000 | 1500 | 40 | 10 | 65 | 4.6 | 0.1 | 4.31 | 16 | 73.06 | 0.06 | 10.284 |
| 5000 | 1635 | 40 | 10 | 65 | 4.6 | 0.1 | 4.31 | 16 | 73.06 | 0.06 | 10.284 |
| 6000 | 1750 | 40 | 10 | 65 | 4.6 | 0.1 | 4.31 | 16 | 73.06 | 0.06 | 10.284 |
| 7000 | 1850 | 40 | 10 | 65 | 4.6 | 0.1 | 4.31 | 16 | 73.06 | 0.06 | 10.284 |
| 8000 | 1950 | 40 | 10 | 65 | 4.6 | 0.1 | 4.31 | 16 | 73.06 | 0.06 | 10.284 |
| 9000 | 2045 | 40 | 10 | 65 | 4.6 | 0.1 | 4.31 | 16 | 73.06 | 0.06 | 10.284 |
| 10000 | 2125 | 40 | 10 | 65 | 4.6 | 0.1 | 4.31 | 16 | 73.06 | 0.06 | 10.284 |
| 1 | 85 | 40 | 10 | 65 | 5.2 | 0.1 | 2.95 | 15.65 | 81.16 | 0.06 | 11.765 |
| 2 | 115 | 40 | 10 | 65 | 5.2 | 0.1 | 2.95 | 15.65 | 81.16 | 0.06 | 11.765 |
| 10 | 215 | 40 | 10 | 65 | 5.2 | 0.1 | 2.95 | 15.65 | 81.16 | 0.06 | 11.765 |
| 100 | 530 | 40 | 10 | 65 | 5.2 | 0.1 | 2.95 | 15.65 | 81.16 | 0.06 | 11.765 |
| 500 | 1010 | 40 | 10 | 65 | 5.2 | 0.1 | 2.95 | 15.65 | 81.16 | 0.06 | 11.765 |
| 1000 | 1330 | 40 | 10 | 65 | 5.2 | 0.1 | 2.95 | 15.65 | 81.16 | 0.06 | 11.765 |
| 2000 | 1755 | 40 | 10 | 65 | 5.2 | 0.1 | 2.95 | 15.65 | 81.16 | 0.06 | 11.765 |
| 3000 | 2060 | 40 | 10 | 65 | 5.2 | 0.1 | 2.95 | 15.65 | 81.16 | 0.06 | 11.765 |
| 4000 | 2315 | 40 | 10 | 65 | 5.2 | 0.1 | 2.95 | 15.65 | 81.16 | 0.06 | 11.765 |
| 5000 | 2530 | 40 | 10 | 65 | 5.2 | 0.1 | 2.95 | 15.65 | 81.16 | 0.06 | 11.765 |
| 6000 | 2720 | 40 | 10 | 65 | 5.2 | 0.1 | 2.95 | 15.65 | 81.16 | 0.06 | 11.765 |
| 7000 | 2890 | 40 | 10 | 65 | 5.2 | 0.1 | 2.95 | 15.65 | 81.16 | 0.06 | 11.765 |
| 8000 | 3050 | 40 | 10 | 65 | 5.2 | 0.1 | 2.95 | 15.65 | 81.16 | 0.06 | 11.765 |
| 9000 | 3190 | 40 | 10 | 65 | 5.2 | 0.1 | 2.95 | 15.65 | 81.16 | 0.06 | 11.765 |
| 10000 | 3330 | 40 | 10 | 65 | 5.2 | 0.1 | 2.95 | 15.65 | 81.16 | 0.06 | 11.765 |
| 1 | 240 | 40 | 20 | 65 | 4 | 0.1 | 5.99 | 16.65 | 64.01 | 0.06 | 8.8 |
| 2 | 310 | 40 | 20 | 65 | 4 | 0.1 | 5.99 | 16.65 | 64.01 | 0.06 | 8.8 |
| 10 | 550 | 40 | 20 | 65 | 4 | 0.1 | 5.99 | 16.65 | 64.01 | 0.06 | 8.8 |
| 100 | 1300 | 40 | 20 | 65 | 4 | 0.1 | 5.99 | 16.65 | 64.01 | 0.06 | 8.8 |
| 500 | 2320 | 40 | 20 | 65 | 4 | 0.1 | 5.99 | 16.65 | 64.01 | 0.06 | 8.8 |
| 1000 | 3000 | 40 | 20 | 65 | 4 | 0.1 | 5.99 | 16.65 | 64.01 | 0.06 | 8.8 |
| 2000 | 3850 | 40 | 20 | 65 | 4 | 0.1 | 5.99 | 16.65 | 64.01 | 0.06 | 8.8 |
| 3000 | 4470 | 40 | 20 | 65 | 4 | 0.1 | 5.99 | 16.65 | 64.01 | 0.06 | 8.8 |
| 4000 | 4960 | 40 | 20 | 65 | 4 | 0.1 | 5.99 | 16.65 | 64.01 | 0.06 | 8.8 |
| 5000 | 5390 | 40 | 20 | 65 | 4 | 0.1 | 5.99 | 16.65 | 64.01 | 0.06 | 8.8 |
| 6000 | 5760 | 40 | 20 | 65 | 4 | 0.1 | 5.99 | 16.65 | 64.01 | 0.06 | 8.8 |
| 7000 | 6100 | 40 | 20 | 65 | 4 | 0.1 | 5.99 | 16.65 | 64.01 | 0.06 | 8.8 |
| 8000 | 6400 | 40 | 20 | 65 | 4 | 0.1 | 5.99 | 16.65 | 64.01 | 0.06 | 8.8 |
| 8500 | 6540 | 40 | 20 | 65 | 4 | 0.1 | 5.99 | 16.65 | 64.01 | 0.06 | 8.8 |
| 9000 | 6850 | 40 | 20 | 65 | 4 | 0.1 | 5.99 | 16.65 | 64.01 | 0.06 | 8.8 |
| 1 | 190 | 40 | 20 | 65 | 4.6 | 0.1 | 4.28 | 15.98 | 73.19 | 0.06 | 10.287 |
| 2 | 250 | 40 | 20 | 65 | 4.6 | 0.1 | 4.28 | 15.98 | 73.19 | 0.06 | 10.287 |
| 10 | 450 | 40 | 20 | 65 | 4.6 | 0.1 | 4.28 | 15.98 | 73.19 | 0.06 | 10.287 |
| 100 | 1250 | 40 | 20 | 65 | 4.6 | 0.1 | 4.28 | 15.98 | 73.19 | 0.06 | 10.287 |
| 500 | 2410 | 40 | 20 | 65 | 4.6 | 0.1 | 4.28 | 15.98 | 73.19 | 0.06 | 10.287 |
| 1000 | 3200 | 40 | 20 | 65 | 4.6 | 0.1 | 4.28 | 15.98 | 73.19 | 0.06 | 10.287 |
| 2000 | 4250 | 40 | 20 | 65 | 4.6 | 0.1 | 4.28 | 15.98 | 73.19 | 0.06 | 10.287 |
| 3000 | 5010 | 40 | 20 | 65 | 4.6 | 0.1 | 4.28 | 15.98 | 73.19 | 0.06 | 10.287 |
| **N** | **E_p_** | **T** | **S** | **P** | **C** | **D** | **A** | **M** | **F** | **B** | **E** |
| 4000 | 5640 | 40 | 20 | 65 | 4.6 | 0.1 | 4.28 | 15.98 | 73.19 | 0.06 | 10.287 |
| 5000 | 6170 | 40 | 20 | 65 | 4.6 | 0.1 | 4.28 | 15.98 | 73.19 | 0.06 | 10.287 |
| 6000 | 6650 | 40 | 20 | 65 | 4.6 | 0.1 | 4.28 | 15.98 | 73.19 | 0.06 | 10.287 |
| 7000 | 7080 | 40 | 20 | 65 | 4.6 | 0.1 | 4.28 | 15.98 | 73.19 | 0.06 | 10.287 |
| 8000 | 7480 | 40 | 20 | 65 | 4.6 | 0.1 | 4.28 | 15.98 | 73.19 | 0.06 | 10.287 |
| 8250 | 7690 | 40 | 20 | 65 | 4.6 | 0.1 | 4.28 | 15.98 | 73.19 | 0.06 | 10.287 |
| 1 | 175 | 40 | 20 | 65 | 5.2 | 0.1 | 2.75 | 15.48 | 82.24 | 0.06 | 11.789 |
| 2 | 240 | 40 | 20 | 65 | 5.2 | 0.1 | 2.75 | 15.48 | 82.24 | 0.06 | 11.789 |
| 10 | 480 | 40 | 20 | 65 | 5.2 | 0.1 | 2.75 | 15.48 | 82.24 | 0.06 | 11.789 |
| 100 | 1330 | 40 | 20 | 65 | 5.2 | 0.1 | 2.75 | 15.48 | 82.24 | 0.06 | 11.789 |
| 500 | 2700 | 40 | 20 | 65 | 5.2 | 0.1 | 2.75 | 15.48 | 82.24 | 0.06 | 11.789 |
| 1000 | 3660 | 40 | 20 | 65 | 5.2 | 0.1 | 2.75 | 15.48 | 82.24 | 0.06 | 11.789 |
| 2000 | 4970 | 40 | 20 | 65 | 5.2 | 0.1 | 2.75 | 15.48 | 82.24 | 0.06 | 11.789 |
| 3000 | 5940 | 40 | 20 | 65 | 5.2 | 0.1 | 2.75 | 15.48 | 82.24 | 0.06 | 11.789 |
| 4000 | 6740 | 40 | 20 | 65 | 5.2 | 0.1 | 2.75 | 15.48 | 82.24 | 0.06 | 11.789 |
| 5000 | 7440 | 40 | 20 | 65 | 5.2 | 0.1 | 2.75 | 15.48 | 82.24 | 0.06 | 11.789 |
| 600 | 2920 | 40 | 20 | 65 | 5.2 | 0.1 | 2.75 | 15.48 | 82.24 | 0.06 | 11.789 |
| 7000 | 8630 | 40 | 20 | 65 | 5.2 | 0.1 | 2.75 | 15.48 | 82.24 | 0.06 | 11.789 |
| 7500 | 9010 | 40 | 20 | 65 | 5.2 | 0.1 | 2.75 | 15.48 | 82.24 | 0.06 | 11.789 |
| 1 | 320 | 40 | 30 | 65 | 4 | 0.1 | 5.96 | 16.62 | 64.13 | 0.06 | 8.803 |
| 2 | 420 | 40 | 30 | 65 | 4 | 0.1 | 5.96 | 16.62 | 64.13 | 0.06 | 8.803 |
| 10 | 760 | 40 | 30 | 65 | 4 | 0.1 | 5.96 | 16.62 | 64.13 | 0.06 | 8.803 |
| 100 | 1790 | 40 | 30 | 65 | 4 | 0.1 | 5.96 | 16.62 | 64.13 | 0.06 | 8.803 |
| 500 | 3260 | 40 | 30 | 65 | 4 | 0.1 | 5.96 | 16.62 | 64.13 | 0.06 | 8.803 |
| 1000 | 4220 | 40 | 30 | 65 | 4 | 0.1 | 5.96 | 16.62 | 64.13 | 0.06 | 8.803 |
| 2000 | 5460 | 40 | 30 | 65 | 4 | 0.1 | 5.96 | 16.62 | 64.13 | 0.06 | 8.803 |
| 3000 | 6360 | 40 | 30 | 65 | 4 | 0.1 | 5.96 | 16.62 | 64.13 | 0.06 | 8.803 |
| 4000 | 7080 | 40 | 30 | 65 | 4 | 0.1 | 5.96 | 16.62 | 64.13 | 0.06 | 8.803 |
| 4500 | 7400 | 40 | 30 | 65 | 4 | 0.1 | 5.96 | 16.62 | 64.13 | 0.06 | 8.803 |
| 4850 | 7860 | 40 | 30 | 65 | 4 | 0.1 | 5.96 | 16.62 | 64.13 | 0.06 | 8.803 |
| 1 | 220 | 40 | 30 | 65 | 4.6 | 0.1 | 4.23 | 15.93 | 73.44 | 0.06 | 10.292 |
| 2 | 300 | 40 | 30 | 65 | 4.6 | 0.1 | 4.23 | 15.93 | 73.44 | 0.06 | 10.292 |
| 10 | 600 | 40 | 30 | 65 | 4.6 | 0.1 | 4.23 | 15.93 | 73.44 | 0.06 | 10.292 |
| 100 | 1640 | 40 | 30 | 65 | 4.6 | 0.1 | 4.23 | 15.93 | 73.44 | 0.06 | 10.292 |
| 500 | 3320 | 40 | 30 | 65 | 4.6 | 0.1 | 4.23 | 15.93 | 73.44 | 0.06 | 10.292 |
| 1000 | 4490 | 40 | 30 | 65 | 4.6 | 0.1 | 4.23 | 15.93 | 73.44 | 0.06 | 10.292 |
| 2000 | 6090 | 40 | 30 | 65 | 4.6 | 0.1 | 4.23 | 15.93 | 73.44 | 0.06 | 10.292 |
| 3000 | 7270 | 40 | 30 | 65 | 4.6 | 0.1 | 4.23 | 15.93 | 73.44 | 0.06 | 10.292 |
| 4000 | 8240 | 40 | 30 | 65 | 4.6 | 0.1 | 4.23 | 15.93 | 73.44 | 0.06 | 10.292 |
| 4500 | 8930 | 40 | 30 | 65 | 4.6 | 0.1 | 4.23 | 15.93 | 73.44 | 0.06 | 10.292 |
| 1 | 280 | 40 | 30 | 65 | 5.2 | 0.1 | 2.67 | 15.41 | 82.68 | 0.06 | 11.799 |
| 2 | 390 | 40 | 30 | 65 | 5.2 | 0.1 | 2.67 | 15.41 | 82.68 | 0.06 | 11.799 |
| 10 | 810 | 40 | 30 | 65 | 5.2 | 0.1 | 2.67 | 15.41 | 82.68 | 0.06 | 11.799 |
| 100 | 2350 | 40 | 30 | 65 | 5.2 | 0.1 | 2.67 | 15.41 | 82.68 | 0.06 | 11.799 |
| 500 | 4960 | 40 | 30 | 65 | 5.2 | 0.1 | 2.67 | 15.41 | 82.68 | 0.06 | 11.799 |
| 1000 | 6840 | 40 | 30 | 65 | 5.2 | 0.1 | 2.67 | 15.41 | 82.68 | 0.06 | 11.799 |
| 2000 | 9420 | 40 | 30 | 65 | 5.2 | 0.1 | 2.67 | 15.41 | 82.68 | 0.06 | 11.799 |
| 3000 | 11360 | 40 | 30 | 65 | 5.2 | 0.1 | 2.67 | 15.41 | 82.68 | 0.06 | 11.799 |
| 4000 | 12980 | 40 | 30 | 65 | 5.2 | 0.1 | 2.67 | 15.41 | 82.68 | 0.06 | 11.799 |
| 4250 | 13340 | 40 | 30 | 65 | 5.2 | 0.1 | 2.67 | 15.41 | 82.68 | 0.06 | 11.799 |
| 4350 | 14050 | 40 | 30 | 65 | 5.2 | 0.1 | 2.67 | 15.41 | 82.68 | 0.06 | 11.799 |
| 1 | 220 | 40 | 10 | 65 | 4 | 0.4 | 6.63 | 17.21 | 61.49 | 0.06 | 8.741 |
| **N** | **E_p_** | **T** | **S** | **P** | **C** | **D** | **A** | **M** | **F** | **B** | **E** |
| 2 | 290 | 40 | 10 | 65 | 4 | 0.4 | 6.63 | 17.21 | 61.49 | 0.06 | 8.741 |
| 10 | 550 | 40 | 10 | 65 | 4 | 0.4 | 6.63 | 17.21 | 61.49 | 0.06 | 8.741 |
| 100 | 1380 | 40 | 10 | 65 | 4 | 0.4 | 6.63 | 17.21 | 61.49 | 0.06 | 8.741 |
| 500 | 2630 | 40 | 10 | 65 | 4 | 0.4 | 6.63 | 17.21 | 61.49 | 0.06 | 8.741 |
| 1000 | 3470 | 40 | 10 | 65 | 4 | 0.4 | 6.63 | 17.21 | 61.49 | 0.06 | 8.741 |
| 2000 | 4580 | 40 | 10 | 65 | 4 | 0.4 | 6.63 | 17.21 | 61.49 | 0.06 | 8.741 |
| 3000 | 5380 | 40 | 10 | 65 | 4 | 0.4 | 6.63 | 17.21 | 61.49 | 0.06 | 8.741 |
| 4000 | 6040 | 40 | 10 | 65 | 4 | 0.4 | 6.63 | 17.21 | 61.49 | 0.06 | 8.741 |
| 4250 | 6190 | 40 | 10 | 65 | 4 | 0.4 | 6.63 | 17.21 | 61.49 | 0.06 | 8.741 |
| 4350 | 6320 | 40 | 10 | 65 | 4 | 0.4 | 6.63 | 17.21 | 61.49 | 0.06 | 8.741 |
| 1 | 120 | 40 | 10 | 65 | 4.6 | 0.4 | 4.08 | 15.79 | 74.19 | 0.06 | 10.309 |
| 2 | 160 | 40 | 10 | 65 | 4.6 | 0.4 | 4.08 | 15.79 | 74.19 | 0.06 | 10.309 |
| 10 | 320 | 40 | 10 | 65 | 4.6 | 0.4 | 4.08 | 15.79 | 74.19 | 0.06 | 10.309 |
| 100 | 840 | 40 | 10 | 65 | 4.6 | 0.4 | 4.08 | 15.79 | 74.19 | 0.06 | 10.309 |
| 500 | 1670 | 40 | 10 | 65 | 4.6 | 0.4 | 4.08 | 15.79 | 74.19 | 0.06 | 10.309 |
| 1000 | 2240 | 40 | 10 | 65 | 4.6 | 0.4 | 4.08 | 15.79 | 74.19 | 0.06 | 10.309 |
| 2000 | 3010 | 40 | 10 | 65 | 4.6 | 0.4 | 4.08 | 15.79 | 74.19 | 0.06 | 10.309 |
| 3000 | 3580 | 40 | 10 | 65 | 4.6 | 0.4 | 4.08 | 15.79 | 74.19 | 0.06 | 10.309 |
| 4000 | 4040 | 40 | 10 | 65 | 4.6 | 0.4 | 4.08 | 15.79 | 74.19 | 0.06 | 10.309 |
| 4250 | 4150 | 40 | 10 | 65 | 4.6 | 0.4 | 4.08 | 15.79 | 74.19 | 0.06 | 10.309 |
| 4446 | 4460 | 40 | 10 | 65 | 4.6 | 0.4 | 4.08 | 15.79 | 74.19 | 0.06 | 10.309 |
| 1 | 155 | 40 | 10 | 65 | 5.2 | 0.4 | 2.74 | 15.47 | 82.3 | 0.06 | 11.79 |
| 2 | 210 | 40 | 10 | 65 | 5.2 | 0.4 | 2.74 | 15.47 | 82.3 | 0.06 | 11.79 |
| 10 | 430 | 40 | 10 | 65 | 5.2 | 0.4 | 2.74 | 15.47 | 82.3 | 0.06 | 11.79 |
| 100 | 1200 | 40 | 10 | 65 | 5.2 | 0.4 | 2.74 | 15.47 | 82.3 | 0.06 | 11.79 |
| 500 | 2460 | 40 | 10 | 65 | 5.2 | 0.4 | 2.74 | 15.47 | 82.3 | 0.06 | 11.79 |
| 1000 | 3350 | 40 | 10 | 65 | 5.2 | 0.4 | 2.74 | 15.47 | 82.3 | 0.06 | 11.79 |
| 2000 | 4560 | 40 | 10 | 65 | 5.2 | 0.4 | 2.74 | 15.47 | 82.3 | 0.06 | 11.79 |
| 3000 | 5470 | 40 | 10 | 65 | 5.2 | 0.4 | 2.74 | 15.47 | 82.3 | 0.06 | 11.79 |
| 4000 | 6210 | 40 | 10 | 65 | 5.2 | 0.4 | 2.74 | 15.47 | 82.3 | 0.06 | 11.79 |
| 4250 | 6450 | 40 | 10 | 65 | 5.2 | 0.4 | 2.74 | 15.47 | 82.3 | 0.06 | 11.79 |
| 1 | 580 | 40 | 20 | 65 | 4 | 0.4 | 6.36 | 16.97 | 62.55 | 0.06 | 8.766 |
| 2 | 770 | 40 | 20 | 65 | 4 | 0.4 | 6.36 | 16.97 | 62.55 | 0.06 | 8.766 |
| 10 | 1520 | 40 | 20 | 65 | 4 | 0.4 | 6.36 | 16.97 | 62.55 | 0.06 | 8.766 |
| 100 | 4010 | 40 | 20 | 65 | 4 | 0.4 | 6.36 | 16.97 | 62.55 | 0.06 | 8.766 |
| 500 | 7880 | 40 | 20 | 65 | 4 | 0.4 | 6.36 | 16.97 | 62.55 | 0.06 | 8.766 |
| 1000 | 10540 | 40 | 20 | 65 | 4 | 0.4 | 6.36 | 16.97 | 62.55 | 0.06 | 8.766 |
| 2000 | 14110 | 40 | 20 | 65 | 4 | 0.4 | 6.36 | 16.97 | 62.55 | 0.06 | 8.766 |
| 3000 | 16730 | 40 | 20 | 65 | 4 | 0.4 | 6.36 | 16.97 | 62.55 | 0.06 | 8.766 |
| 4000 | 18880 | 40 | 20 | 65 | 4 | 0.4 | 6.36 | 16.97 | 62.55 | 0.06 | 8.766 |
| 4500 | 21300 | 40 | 20 | 65 | 4 | 0.4 | 6.36 | 16.97 | 62.55 | 0.06 | 8.766 |
| 1 | 420 | 40 | 20 | 65 | 4.6 | 0.4 | 4.36 | 16.04 | 72.82 | 0.06 | 10.278 |
| 2 | 580 | 40 | 20 | 65 | 4.6 | 0.4 | 4.36 | 16.04 | 72.82 | 0.06 | 10.278 |
| 10 | 1220 | 40 | 20 | 65 | 4.6 | 0.4 | 4.36 | 16.04 | 72.82 | 0.06 | 10.278 |
| 100 | 3570 | 40 | 20 | 65 | 4.6 | 0.4 | 4.36 | 16.04 | 72.82 | 0.06 | 10.278 |
| 500 | 7540 | 40 | 20 | 65 | 4.6 | 0.4 | 4.36 | 16.04 | 72.82 | 0.06 | 10.278 |
| 1000 | 10420 | 40 | 20 | 65 | 4.6 | 0.4 | 4.36 | 16.04 | 72.82 | 0.06 | 10.278 |
| 2000 | 14380 | 40 | 20 | 65 | 4.6 | 0.4 | 4.36 | 16.04 | 72.82 | 0.06 | 10.278 |
| 3000 | 17360 | 40 | 20 | 65 | 4.6 | 0.4 | 4.36 | 16.04 | 72.82 | 0.06 | 10.278 |
| 4000 | 19840 | 40 | 20 | 65 | 4.6 | 0.4 | 4.36 | 16.04 | 72.82 | 0.06 | 10.278 |
| 4400 | 21890 | 40 | 20 | 65 | 4.6 | 0.4 | 4.36 | 16.04 | 72.82 | 0.06 | 10.278 |
| 1 | 400 | 40 | 20 | 65 | 5.2 | 0.4 | 2.83 | 15.55 | 81.81 | 0.06 | 11.78 |
| **N** | **E_p_** | **T** | **S** | **P** | **C** | **D** | **A** | **M** | **F** | **B** | **E** |
| 2 | 560 | 40 | 20 | 65 | 5.2 | 0.4 | 2.83 | 15.55 | 81.81 | 0.06 | 11.78 |
| 10 | 1250 | 40 | 20 | 65 | 5.2 | 0.4 | 2.83 | 15.55 | 81.81 | 0.06 | 11.78 |
| 100 | 3920 | 40 | 20 | 65 | 5.2 | 0.4 | 2.83 | 15.55 | 81.81 | 0.06 | 11.78 |
| 500 | 8700 | 40 | 20 | 65 | 5.2 | 0.4 | 2.83 | 15.55 | 81.81 | 0.06 | 11.78 |
| 1000 | 12270 | 40 | 20 | 65 | 5.2 | 0.4 | 2.83 | 15.55 | 81.81 | 0.06 | 11.78 |
| 2000 | 17300 | 40 | 20 | 65 | 5.2 | 0.4 | 2.83 | 15.55 | 81.81 | 0.06 | 11.78 |
| 3000 | 21150 | 40 | 20 | 65 | 5.2 | 0.4 | 2.83 | 15.55 | 81.81 | 0.06 | 11.78 |
| 4000 | 25890 | 40 | 20 | 65 | 5.2 | 0.4 | 2.83 | 15.55 | 81.81 | 0.06 | 11.78 |
| 1 | 590 | 40 | 30 | 65 | 4 | 0.4 | 6.54 | 17.13 | 61.84 | 0.06 | 8.749 |
| 2 | 800 | 40 | 30 | 65 | 4 | 0.4 | 6.54 | 17.13 | 61.84 | 0.06 | 8.749 |
| 10 | 1650 | 40 | 30 | 65 | 4 | 0.4 | 6.54 | 17.13 | 61.84 | 0.06 | 8.749 |
| 100 | 4630 | 40 | 30 | 65 | 4 | 0.4 | 6.54 | 17.13 | 61.84 | 0.06 | 8.749 |
| 500 | 9500 | 40 | 30 | 65 | 4 | 0.4 | 6.54 | 17.13 | 61.84 | 0.06 | 8.749 |
| 1000 | 12960 | 40 | 30 | 65 | 4 | 0.4 | 6.54 | 17.13 | 61.84 | 0.06 | 8.749 |
| 1250 | 14320 | 40 | 30 | 65 | 4 | 0.4 | 6.54 | 17.13 | 61.84 | 0.06 | 8.749 |
| 1500 | 15540 | 40 | 30 | 65 | 4 | 0.4 | 6.54 | 17.13 | 61.84 | 0.06 | 8.749 |
| 1750 | 16650 | 40 | 30 | 65 | 4 | 0.4 | 6.54 | 17.13 | 61.84 | 0.06 | 8.749 |
| 1900 | 17850 | 40 | 30 | 65 | 4 | 0.4 | 6.54 | 17.13 | 61.84 | 0.06 | 8.749 |
| 1 | 440 | 40 | 30 | 65 | 4.6 | 0.4 | 4.11 | 15.82 | 74.04 | 0.06 | 10.305 |
| 2 | 620 | 40 | 30 | 65 | 4.6 | 0.4 | 4.11 | 15.82 | 74.04 | 0.06 | 10.305 |
| 10 | 1400 | 40 | 30 | 65 | 4.6 | 0.4 | 4.11 | 15.82 | 74.04 | 0.06 | 10.305 |
| 100 | 4460 | 40 | 30 | 65 | 4.6 | 0.4 | 4.11 | 15.82 | 74.04 | 0.06 | 10.305 |
| 500 | 10040 | 40 | 30 | 65 | 4.6 | 0.4 | 4.11 | 15.82 | 74.04 | 0.06 | 10.305 |
| 1000 | 14230 | 40 | 30 | 65 | 4.6 | 0.4 | 4.11 | 15.82 | 74.04 | 0.06 | 10.305 |
| 1250 | 15930 | 40 | 30 | 65 | 4.6 | 0.4 | 4.11 | 15.82 | 74.04 | 0.06 | 10.305 |
| 1500 | 17450 | 40 | 30 | 65 | 4.6 | 0.4 | 4.11 | 15.82 | 74.04 | 0.06 | 10.305 |
| 1750 | 18860 | 40 | 30 | 65 | 4.6 | 0.4 | 4.11 | 15.82 | 74.04 | 0.06 | 10.305 |
| 1800 | 19130 | 40 | 30 | 65 | 4.6 | 0.4 | 4.11 | 15.82 | 74.04 | 0.06 | 10.305 |
| 1850 | 20530 | 40 | 30 | 65 | 4.6 | 0.4 | 4.11 | 15.82 | 74.04 | 0.06 | 10.305 |
| 1 | 455 | 40 | 30 | 65 | 5.2 | 0.4 | 2.89 | 15.6 | 81.49 | 0.06 | 11.772 |
| 2 | 660 | 40 | 30 | 65 | 5.2 | 0.4 | 2.89 | 15.6 | 81.49 | 0.06 | 11.772 |
| 10 | 1550 | 40 | 30 | 65 | 5.2 | 0.4 | 2.89 | 15.6 | 81.49 | 0.06 | 11.772 |
| 100 | 5320 | 40 | 30 | 65 | 5.2 | 0.4 | 2.89 | 15.6 | 81.49 | 0.06 | 11.772 |
| 500 | 12570 | 40 | 30 | 65 | 5.2 | 0.4 | 2.89 | 15.6 | 81.49 | 0.06 | 11.772 |
| 1000 | 18210 | 40 | 30 | 65 | 5.2 | 0.4 | 2.89 | 15.6 | 81.49 | 0.06 | 11.772 |
| 1250 | 20510 | 40 | 30 | 65 | 5.2 | 0.4 | 2.89 | 15.6 | 81.49 | 0.06 | 11.772 |
| 1500 | 22610 | 40 | 30 | 65 | 5.2 | 0.4 | 2.89 | 15.6 | 81.49 | 0.06 | 11.772 |
| 1750 | 25640 | 40 | 30 | 65 | 5.2 | 0.4 | 2.89 | 15.6 | 81.49 | 0.06 | 11.772 |
| 1 | 195 | 60 | 10 | 44 | 4 | 0.1 | 6.36 | 16.3 | 60.95 | 0.061 | 8.725 |
| 2 | 280 | 60 | 10 | 44 | 4 | 0.1 | 6.36 | 16.3 | 60.95 | 0.061 | 8.725 |
| 10 | 660 | 60 | 10 | 44 | 4 | 0.1 | 6.36 | 16.3 | 60.95 | 0.061 | 8.725 |
| 100 | 2230 | 60 | 10 | 44 | 4 | 0.1 | 6.36 | 16.3 | 60.95 | 0.061 | 8.725 |
| 500 | 5230 | 60 | 10 | 44 | 4 | 0.1 | 6.36 | 16.3 | 60.95 | 0.061 | 8.725 |
| 1000 | 7550 | 60 | 10 | 44 | 4 | 0.1 | 6.36 | 16.3 | 60.95 | 0.061 | 8.725 |
| 2000 | 10900 | 60 | 10 | 44 | 4 | 0.1 | 6.36 | 16.3 | 60.95 | 0.061 | 8.725 |
| 3000 | 13510 | 60 | 10 | 44 | 4 | 0.1 | 6.36 | 16.3 | 60.95 | 0.061 | 8.725 |
| 4000 | 15740 | 60 | 10 | 44 | 4 | 0.1 | 6.36 | 16.3 | 60.95 | 0.061 | 8.725 |
| 4250 | 16250 | 60 | 10 | 44 | 4 | 0.1 | 6.36 | 16.3 | 60.95 | 0.061 | 8.725 |
| 4325 | 16890 | 60 | 10 | 44 | 4 | 0.1 | 6.36 | 16.3 | 60.95 | 0.061 | 8.725 |
| 1 | 90 | 60 | 10 | 44 | 4.6 | 0.1 | 3.95 | 15.27 | 74.11 | 0.061 | 10.241 |
| 2 | 130 | 60 | 10 | 44 | 4.6 | 0.1 | 3.95 | 15.27 | 74.11 | 0.061 | 10.241 |
| 10 | 330 | 60 | 10 | 44 | 4.6 | 0.1 | 3.95 | 15.27 | 74.11 | 0.061 | 10.241 |
| **N** | **E_p_** | **T** | **S** | **P** | **C** | **D** | **A** | **M** | **F** | **B** | **E** |
| 100 | 1240 | 60 | 10 | 44 | 4.6 | 0.1 | 3.95 | 15.27 | 74.11 | 0.061 | 10.241 |
| 500 | 3090 | 60 | 10 | 44 | 4.6 | 0.1 | 3.95 | 15.27 | 74.11 | 0.061 | 10.241 |
| 1000 | 4590 | 60 | 10 | 44 | 4.6 | 0.1 | 3.95 | 15.27 | 74.11 | 0.061 | 10.241 |
| 2000 | 6820 | 60 | 10 | 44 | 4.6 | 0.1 | 3.95 | 15.27 | 74.11 | 0.061 | 10.241 |
| 3000 | 8590 | 60 | 10 | 44 | 4.6 | 0.1 | 3.95 | 15.27 | 74.11 | 0.061 | 10.241 |
| 4000 | 10120 | 60 | 10 | 44 | 4.6 | 0.1 | 3.95 | 15.27 | 74.11 | 0.061 | 10.241 |
| 4200 | 11000 | 60 | 10 | 44 | 4.6 | 0.1 | 3.95 | 15.27 | 74.11 | 0.061 | 10.241 |
| 1 | 50 | 60 | 10 | 44 | 5.2 | 0.1 | 3.02 | 15.27 | 80.19 | 0.061 | 11.669 |
| 2 | 70 | 60 | 10 | 44 | 5.2 | 0.1 | 3.02 | 15.27 | 80.19 | 0.061 | 11.669 |
| 10 | 190 | 60 | 10 | 44 | 5.2 | 0.1 | 3.02 | 15.27 | 80.19 | 0.061 | 11.669 |
| 100 | 780 | 60 | 10 | 44 | 5.2 | 0.1 | 3.02 | 15.27 | 80.19 | 0.061 | 11.669 |
| 500 | 2040 | 60 | 10 | 44 | 5.2 | 0.1 | 3.02 | 15.27 | 80.19 | 0.061 | 11.669 |
| 1000 | 3090 | 60 | 10 | 44 | 5.2 | 0.1 | 3.02 | 15.27 | 80.19 | 0.061 | 11.669 |
| 2000 | 4670 | 60 | 10 | 44 | 5.2 | 0.1 | 3.02 | 15.27 | 80.19 | 0.061 | 11.669 |
| 3000 | 5950 | 60 | 10 | 44 | 5.2 | 0.1 | 3.02 | 15.27 | 80.19 | 0.061 | 11.669 |
| 3750 | 6800 | 60 | 10 | 44 | 5.2 | 0.1 | 3.02 | 15.27 | 80.19 | 0.061 | 11.669 |
| 3800 | 7350 | 60 | 10 | 44 | 5.2 | 0.1 | 3.02 | 15.27 | 80.19 | 0.061 | 11.669 |
| 1 | 335 | 60 | 20 | 44 | 4 | 0.1 | 6.53 | 16.45 | 60.31 | 0.061 | 8.709 |
| 2 | 490 | 60 | 20 | 44 | 4 | 0.1 | 6.53 | 16.45 | 60.31 | 0.061 | 8.709 |
| 10 | 1160 | 60 | 20 | 44 | 4 | 0.1 | 6.53 | 16.45 | 60.31 | 0.061 | 8.709 |
| 100 | 4000 | 60 | 20 | 44 | 4 | 0.1 | 6.53 | 16.45 | 60.31 | 0.061 | 8.709 |
| 500 | 9540 | 60 | 20 | 44 | 4 | 0.1 | 6.53 | 16.45 | 60.31 | 0.061 | 8.709 |
| 1000 | 13860 | 60 | 20 | 44 | 4 | 0.1 | 6.53 | 16.45 | 60.31 | 0.061 | 8.709 |
| 1500 | 17240 | 60 | 20 | 44 | 4 | 0.1 | 6.53 | 16.45 | 60.31 | 0.061 | 8.709 |
| 1600 | 17850 | 60 | 20 | 44 | 4 | 0.1 | 6.53 | 16.45 | 60.31 | 0.061 | 8.709 |
| 1650 | 18150 | 60 | 20 | 44 | 4 | 0.1 | 6.53 | 16.45 | 60.31 | 0.061 | 8.709 |
| 1685 | 19000 | 60 | 20 | 44 | 4 | 0.1 | 6.53 | 16.45 | 60.31 | 0.061 | 8.709 |
| 1 | 310 | 60 | 20 | 44 | 4.6 | 0.1 | 4.39 | 15.65 | 71.98 | 0.061 | 10.195 |
| 2 | 470 | 60 | 20 | 44 | 4.6 | 0.1 | 4.39 | 15.65 | 71.98 | 0.061 | 10.195 |
| 10 | 1260 | 60 | 20 | 44 | 4.6 | 0.1 | 4.39 | 15.65 | 71.98 | 0.061 | 10.195 |
| 100 | 5140 | 60 | 20 | 44 | 4.6 | 0.1 | 4.39 | 15.65 | 71.98 | 0.061 | 10.195 |
| 500 | 13720 | 60 | 20 | 44 | 4.6 | 0.1 | 4.39 | 15.65 | 71.98 | 0.061 | 10.195 |
| 1000 | 20940 | 60 | 20 | 44 | 4.6 | 0.1 | 4.39 | 15.65 | 71.98 | 0.061 | 10.195 |
| 1500 | 26820 | 60 | 20 | 44 | 4.6 | 0.1 | 4.39 | 15.65 | 71.98 | 0.061 | 10.195 |
| 1550 | 27360 | 60 | 20 | 44 | 4.6 | 0.1 | 4.39 | 15.65 | 71.98 | 0.061 | 10.195 |
| 1600 | 29100 | 60 | 20 | 44 | 4.6 | 0.1 | 4.39 | 15.65 | 71.98 | 0.061 | 10.195 |
| 1 | 390 | 60 | 20 | 44 | 5.2 | 0.1 | 3.12 | 15.36 | 79.65 | 0.061 | 11.657 |
| 2 | 600 | 60 | 20 | 44 | 5.2 | 0.1 | 3.12 | 15.36 | 79.65 | 0.061 | 11.657 |
| 10 | 1690 | 60 | 20 | 44 | 5.2 | 0.1 | 3.12 | 15.36 | 79.65 | 0.061 | 11.657 |
| 100 | 7340 | 60 | 20 | 44 | 5.2 | 0.1 | 3.12 | 15.36 | 79.65 | 0.061 | 11.657 |
| 500 | 20470 | 60 | 20 | 44 | 5.2 | 0.1 | 3.12 | 15.36 | 79.65 | 0.061 | 11.657 |
| 1000 | 31840 | 60 | 20 | 44 | 5.2 | 0.1 | 3.12 | 15.36 | 79.65 | 0.061 | 11.657 |
| 1300 | 37630 | 60 | 20 | 44 | 5.2 | 0.1 | 3.12 | 15.36 | 79.65 | 0.061 | 11.657 |
| 1400 | 39450 | 60 | 20 | 44 | 5.2 | 0.1 | 3.12 | 15.36 | 79.65 | 0.061 | 11.657 |
| 1425 | 40300 | 60 | 20 | 44 | 5.2 | 0.1 | 3.12 | 15.36 | 79.65 | 0.061 | 11.657 |
| 1 | 560 | 60 | 30 | 44 | 4 | 0.1 | 6.58 | 16.49 | 60.09 | 0.061 | 8.704 |
| 2 | 850 | 60 | 30 | 44 | 4 | 0.1 | 6.58 | 16.49 | 60.09 | 0.061 | 8.704 |
| 10 | 2250 | 60 | 30 | 44 | 4 | 0.1 | 6.58 | 16.49 | 60.09 | 0.061 | 8.704 |
| 100 | 9060 | 60 | 30 | 44 | 4 | 0.1 | 6.58 | 16.49 | 60.09 | 0.061 | 8.704 |
| 500 | 23970 | 60 | 30 | 44 | 4 | 0.1 | 6.58 | 16.49 | 60.09 | 0.061 | 8.704 |
| 600 | 26760 | 60 | 30 | 44 | 4 | 0.1 | 6.58 | 16.49 | 60.09 | 0.061 | 8.704 |
| 650 | 28090 | 60 | 30 | 44 | 4 | 0.1 | 6.58 | 16.49 | 60.09 | 0.061 | 8.704 |
| **N** | **E_p_** | **T** | **S** | **P** | **C** | **D** | **A** | **M** | **F** | **B** | **E** |
| 700 | 29380 | 60 | 30 | 44 | 4 | 0.1 | 6.58 | 16.49 | 60.09 | 0.061 | 8.704 |
| 710 | 30600 | 60 | 30 | 44 | 4 | 0.1 | 6.58 | 16.49 | 60.09 | 0.061 | 8.704 |
| 1 | 610 | 60 | 30 | 44 | 4.6 | 0.1 | 3.98 | 15.29 | 73.99 | 0.061 | 10.239 |
| 2 | 990 | 60 | 30 | 44 | 4.6 | 0.1 | 3.98 | 15.29 | 73.99 | 0.061 | 10.239 |
| 10 | 3000 | 60 | 30 | 44 | 4.6 | 0.1 | 3.98 | 15.29 | 73.99 | 0.061 | 10.239 |
| 100 | 14800 | 60 | 30 | 44 | 4.6 | 0.1 | 3.98 | 15.29 | 73.99 | 0.061 | 10.239 |
| 500 | 45120 | 60 | 30 | 44 | 4.6 | 0.1 | 3.98 | 15.29 | 73.99 | 0.061 | 10.239 |
| 600 | 51190 | 60 | 30 | 44 | 4.6 | 0.1 | 3.98 | 15.29 | 73.99 | 0.061 | 10.239 |
| 650 | 54110 | 60 | 30 | 44 | 4.6 | 0.1 | 3.98 | 15.29 | 73.99 | 0.061 | 10.239 |
| 700 | 56960 | 60 | 30 | 44 | 4.6 | 0.1 | 3.98 | 15.29 | 73.99 | 0.061 | 10.239 |
| 725 | 60000 | 60 | 30 | 44 | 4.6 | 0.1 | 3.98 | 15.29 | 73.99 | 0.061 | 10.239 |
| 1 | 680 | 60 | 30 | 44 | 5.2 | 0.1 | 2.92 | 15.17 | 80.78 | 0.061 | 11.682 |
| 2 | 1140 | 60 | 30 | 44 | 5.2 | 0.1 | 2.92 | 15.17 | 80.78 | 0.061 | 11.682 |
| 10 | 3780 | 60 | 30 | 44 | 5.2 | 0.1 | 2.92 | 15.17 | 80.78 | 0.061 | 11.682 |
| 100 | 21030 | 60 | 30 | 44 | 5.2 | 0.1 | 2.92 | 15.17 | 80.78 | 0.061 | 11.682 |
| 500 | 69780 | 60 | 30 | 44 | 5.2 | 0.1 | 2.92 | 15.17 | 80.78 | 0.061 | 11.682 |
| 600 | 79940 | 60 | 30 | 44 | 5.2 | 0.1 | 2.92 | 15.17 | 80.78 | 0.061 | 11.682 |
| 630 | 82900 | 60 | 30 | 44 | 5.2 | 0.1 | 2.92 | 15.17 | 80.78 | 0.061 | 11.682 |
| 1 | 275 | 60 | 10 | 44 | 4 | 0.4 | 6.36 | 16.3 | 60.95 | 0.061 | 8.725 |
| 2 | 415 | 60 | 10 | 44 | 4 | 0.4 | 6.36 | 16.3 | 60.95 | 0.061 | 8.725 |
| 10 | 1070 | 60 | 10 | 44 | 4 | 0.4 | 6.36 | 16.3 | 60.95 | 0.061 | 8.725 |
| 100 | 4220 | 60 | 10 | 44 | 4 | 0.4 | 6.36 | 16.3 | 60.95 | 0.061 | 8.725 |
| 500 | 10970 | 60 | 10 | 44 | 4 | 0.4 | 6.36 | 16.3 | 60.95 | 0.061 | 8.725 |
| 1000 | 16550 | 60 | 10 | 44 | 4 | 0.4 | 6.36 | 16.3 | 60.95 | 0.061 | 8.725 |
| 1500 | 21050 | 60 | 10 | 44 | 4 | 0.4 | 6.36 | 16.3 | 60.95 | 0.061 | 8.725 |
| 1600 | 21870 | 60 | 10 | 44 | 4 | 0.4 | 6.36 | 16.3 | 60.95 | 0.061 | 8.725 |
| 1700 | 22680 | 60 | 10 | 44 | 4 | 0.4 | 6.36 | 16.3 | 60.95 | 0.061 | 8.725 |
| 1800 | 23460 | 60 | 10 | 44 | 4 | 0.4 | 6.36 | 16.3 | 60.95 | 0.061 | 8.725 |
| 1900 | 24900 | 60 | 10 | 44 | 4 | 0.4 | 6.36 | 16.3 | 60.95 | 0.061 | 8.725 |
| 2000 | 28300 | 60 | 10 | 44 | 4 | 0.4 | 6.36 | 16.3 | 60.95 | 0.061 | 8.725 |
| 1 | 130 | 60 | 10 | 44 | 4.6 | 0.4 | 4.01 | 15.32 | 73.84 | 0.061 | 10.236 |
| 2 | 200 | 60 | 10 | 44 | 4.6 | 0.4 | 4.01 | 15.32 | 73.84 | 0.061 | 10.236 |
| 10 | 560 | 60 | 10 | 44 | 4.6 | 0.4 | 4.01 | 15.32 | 73.84 | 0.061 | 10.236 |
| 100 | 2430 | 60 | 10 | 44 | 4.6 | 0.4 | 4.01 | 15.32 | 73.84 | 0.061 | 10.236 |
| 500 | 6770 | 60 | 10 | 44 | 4.6 | 0.4 | 4.01 | 15.32 | 73.84 | 0.061 | 10.236 |
| 1000 | 10530 | 60 | 10 | 44 | 4.6 | 0.4 | 4.01 | 15.32 | 73.84 | 0.061 | 10.236 |
| 1500 | 13630 | 60 | 10 | 44 | 4.6 | 0.4 | 4.01 | 15.32 | 73.84 | 0.061 | 10.236 |
| 1600 | 14200 | 60 | 10 | 44 | 4.6 | 0.4 | 4.01 | 15.32 | 73.84 | 0.061 | 10.236 |
| 1700 | 14760 | 60 | 10 | 44 | 4.6 | 0.4 | 4.01 | 15.32 | 73.84 | 0.061 | 10.236 |
| 1800 | 16200 | 60 | 10 | 44 | 4.6 | 0.4 | 4.01 | 15.32 | 73.84 | 0.061 | 10.236 |
| 1900 | 18700 | 60 | 10 | 44 | 4.6 | 0.4 | 4.01 | 15.32 | 73.84 | 0.061 | 10.236 |
| 1 | 140 | 60 | 10 | 44 | 5.2 | 0.4 | 2.74 | 15.02 | 81.78 | 0.061 | 11.704 |
| 2 | 220 | 60 | 10 | 44 | 5.2 | 0.4 | 2.74 | 15.02 | 81.78 | 0.061 | 11.704 |
| 10 | 640 | 60 | 10 | 44 | 5.2 | 0.4 | 2.74 | 15.02 | 81.78 | 0.061 | 11.704 |
| 100 | 2970 | 60 | 10 | 44 | 5.2 | 0.4 | 2.74 | 15.02 | 81.78 | 0.061 | 11.704 |
| 500 | 8630 | 60 | 10 | 44 | 5.2 | 0.4 | 2.74 | 15.02 | 81.78 | 0.061 | 11.704 |
| 1000 | 13670 | 60 | 10 | 44 | 5.2 | 0.4 | 2.74 | 15.02 | 81.78 | 0.061 | 11.704 |
| 1500 | 17900 | 60 | 10 | 44 | 5.2 | 0.4 | 2.74 | 15.02 | 81.78 | 0.061 | 11.704 |
| 1600 | 18680 | 60 | 10 | 44 | 5.2 | 0.4 | 2.74 | 15.02 | 81.78 | 0.061 | 11.704 |
| 1700 | 19450 | 60 | 10 | 44 | 5.2 | 0.4 | 2.74 | 15.02 | 81.78 | 0.061 | 11.704 |
| 1800 | 20200 | 60 | 10 | 44 | 5.2 | 0.4 | 2.74 | 15.02 | 81.78 | 0.061 | 11.704 |
| 1850 | 21500 | 60 | 10 | 44 | 5.2 | 0.4 | 2.74 | 15.02 | 81.78 | 0.061 | 11.704 |
| **N** | **E_p_** | **T** | **S** | **P** | **C** | **D** | **A** | **M** | **F** | **B** | **E** |
| 1 | 380 | 60 | 20 | 44 | 4 | 0.4 | 6.51 | 16.43 | 60.36 | 0.061 | 8.711 |
| 2 | 580 | 60 | 20 | 44 | 4 | 0.4 | 6.51 | 16.43 | 60.36 | 0.061 | 8.711 |
| 10 | 1540 | 60 | 20 | 44 | 4 | 0.4 | 6.51 | 16.43 | 60.36 | 0.061 | 8.711 |
| 100 | 6240 | 60 | 20 | 44 | 4 | 0.4 | 6.51 | 16.43 | 60.36 | 0.061 | 8.711 |
| 500 | 16600 | 60 | 20 | 44 | 4 | 0.4 | 6.51 | 16.43 | 60.36 | 0.061 | 8.711 |
| 525 | 17100 | 60 | 20 | 44 | 4 | 0.4 | 6.51 | 16.43 | 60.36 | 0.061 | 8.711 |
| 550 | 17590 | 60 | 20 | 44 | 4 | 0.4 | 6.51 | 16.43 | 60.36 | 0.061 | 8.711 |
| 600 | 18550 | 60 | 20 | 44 | 4 | 0.4 | 6.51 | 16.43 | 60.36 | 0.061 | 8.711 |
| 650 | 19470 | 60 | 20 | 44 | 4 | 0.4 | 6.51 | 16.43 | 60.36 | 0.061 | 8.711 |
| 700 | 19900 | 60 | 20 | 44 | 4 | 0.4 | 6.51 | 16.43 | 60.36 | 0.061 | 8.711 |
| 750 | 21240 | 60 | 20 | 44 | 4 | 0.4 | 6.51 | 16.43 | 60.36 | 0.061 | 8.711 |
| 800 | 24000 | 60 | 20 | 44 | 4 | 0.4 | 6.51 | 16.43 | 60.36 | 0.061 | 8.711 |
| 1 | 350 | 60 | 20 | 44 | 4.6 | 0.4 | 4 | 15.31 | 73.87 | 0.061 | 10.236 |
| 2 | 560 | 60 | 20 | 44 | 4.6 | 0.4 | 4 | 15.31 | 73.87 | 0.061 | 10.236 |
| 10 | 1680 | 60 | 20 | 44 | 4.6 | 0.4 | 4 | 15.31 | 73.87 | 0.061 | 10.236 |
| 100 | 8130 | 60 | 20 | 44 | 4.6 | 0.4 | 4 | 15.31 | 73.87 | 0.061 | 10.236 |
| 500 | 24420 | 60 | 20 | 44 | 4.6 | 0.4 | 4 | 15.31 | 73.87 | 0.061 | 10.236 |
| 525 | 25240 | 60 | 20 | 44 | 4.6 | 0.4 | 4 | 15.31 | 73.87 | 0.061 | 10.236 |
| 550 | 26060 | 60 | 20 | 44 | 4.6 | 0.4 | 4 | 15.31 | 73.87 | 0.061 | 10.236 |
| 600 | 27660 | 60 | 20 | 44 | 4.6 | 0.4 | 4 | 15.31 | 73.87 | 0.061 | 10.236 |
| 650 | 29200 | 60 | 20 | 44 | 4.6 | 0.4 | 4 | 15.31 | 73.87 | 0.061 | 10.236 |
| 700 | 33100 | 60 | 20 | 44 | 4.6 | 0.4 | 4 | 15.31 | 73.87 | 0.061 | 10.236 |
| 1 | 435 | 60 | 20 | 44 | 5.2 | 0.4 | 2.71 | 15 | 81.9 | 0.061 | 11.706 |
| 2 | 710 | 60 | 20 | 44 | 5.2 | 0.4 | 2.71 | 15 | 81.9 | 0.061 | 11.706 |
| 10 | 2240 | 60 | 20 | 44 | 5.2 | 0.4 | 2.71 | 15 | 81.9 | 0.061 | 11.706 |
| 100 | 11580 | 60 | 20 | 44 | 5.2 | 0.4 | 2.71 | 15 | 81.9 | 0.061 | 11.706 |
| 500 | 36460 | 60 | 20 | 44 | 5.2 | 0.4 | 2.71 | 15 | 81.9 | 0.061 | 11.706 |
| 525 | 37750 | 60 | 20 | 44 | 5.2 | 0.4 | 2.71 | 15 | 81.9 | 0.061 | 11.706 |
| 550 | 39020 | 60 | 20 | 44 | 5.2 | 0.4 | 2.71 | 15 | 81.9 | 0.061 | 11.706 |
| 600 | 41520 | 60 | 20 | 44 | 5.2 | 0.4 | 2.71 | 15 | 81.9 | 0.061 | 11.706 |
| 630 | 44900 | 60 | 20 | 44 | 5.2 | 0.4 | 2.71 | 15 | 81.9 | 0.061 | 11.706 |
| 1 | 820 | 60 | 30 | 44 | 4 | 0.4 | 6.57 | 16.48 | 60.15 | 0.061 | 8.706 |
| 2 | 1350 | 60 | 30 | 44 | 4 | 0.4 | 6.57 | 16.48 | 60.15 | 0.061 | 8.706 |
| 10 | 4300 | 60 | 30 | 44 | 4 | 0.4 | 6.57 | 16.48 | 60.15 | 0.061 | 8.706 |
| 100 | 22580 | 60 | 30 | 44 | 4 | 0.4 | 6.57 | 16.48 | 60.15 | 0.061 | 8.706 |
| 150 | 30240 | 60 | 30 | 44 | 4 | 0.4 | 6.57 | 16.48 | 60.15 | 0.061 | 8.706 |
| 200 | 37200 | 60 | 30 | 44 | 4 | 0.4 | 6.57 | 16.48 | 60.15 | 0.061 | 8.706 |
| 250 | 43680 | 60 | 30 | 44 | 4 | 0.4 | 6.57 | 16.48 | 60.15 | 0.061 | 8.706 |
| 300 | 49810 | 60 | 30 | 44 | 4 | 0.4 | 6.57 | 16.48 | 60.15 | 0.061 | 8.706 |
| 350 | 55660 | 60 | 30 | 44 | 4 | 0.4 | 6.57 | 16.48 | 60.15 | 0.061 | 8.706 |
| 397 | 58400 | 60 | 30 | 44 | 4 | 0.4 | 6.57 | 16.48 | 60.15 | 0.061 | 8.706 |
| 425 | 63200 | 60 | 30 | 44 | 4 | 0.4 | 6.57 | 16.48 | 60.15 | 0.061 | 8.706 |
| 1 | 900 | 60 | 30 | 44 | 4.6 | 0.4 | 3.91 | 15.23 | 74.35 | 0.061 | 10.246 |
| 2 | 1540 | 60 | 30 | 44 | 4.6 | 0.4 | 3.91 | 15.23 | 74.35 | 0.061 | 10.246 |
| 10 | 5430 | 60 | 30 | 44 | 4.6 | 0.4 | 3.91 | 15.23 | 74.35 | 0.061 | 10.246 |
| 100 | 32790 | 60 | 30 | 44 | 4.6 | 0.4 | 3.91 | 15.23 | 74.35 | 0.061 | 10.246 |
| 150 | 45010 | 60 | 30 | 44 | 4.6 | 0.4 | 3.91 | 15.23 | 74.35 | 0.061 | 10.246 |
| 200 | 56340 | 60 | 30 | 44 | 4.6 | 0.4 | 3.91 | 15.23 | 74.35 | 0.061 | 10.246 |
| 250 | 67070 | 60 | 30 | 44 | 4.6 | 0.4 | 3.91 | 15.23 | 74.35 | 0.061 | 10.246 |
| 300 | 77330 | 60 | 30 | 44 | 4.6 | 0.4 | 3.91 | 15.23 | 74.35 | 0.061 | 10.246 |
| 350 | 87220 | 60 | 30 | 44 | 4.6 | 0.4 | 3.91 | 15.23 | 74.35 | 0.061 | 10.246 |
| 400 | 92000 | 60 | 30 | 44 | 4.6 | 0.4 | 3.91 | 15.23 | 74.35 | 0.061 | 10.246 |
| **N** | **E_p_** | **T** | **S** | **P** | **C** | **D** | **A** | **M** | **F** | **B** | **E** |
| 410 | 96600 | 60 | 30 | 44 | 4.6 | 0.4 | 3.91 | 15.23 | 74.35 | 0.061 | 10.246 |
| 1 | 990 | 60 | 30 | 44 | 5.2 | 0.4 | 3.03 | 15.27 | 80.15 | 0.061 | 11.668 |
| 2 | 1670 | 60 | 30 | 44 | 5.2 | 0.4 | 3.03 | 15.27 | 80.15 | 0.061 | 11.668 |
| 10 | 5630 | 60 | 30 | 44 | 5.2 | 0.4 | 3.03 | 15.27 | 80.15 | 0.061 | 11.668 |
| 100 | 32110 | 60 | 30 | 44 | 5.2 | 0.4 | 3.03 | 15.27 | 80.15 | 0.061 | 11.668 |
| 150 | 43620 | 60 | 30 | 44 | 5.2 | 0.4 | 3.03 | 15.27 | 80.15 | 0.061 | 11.668 |
| 200 | 54210 | 60 | 30 | 44 | 5.2 | 0.4 | 3.03 | 15.27 | 80.15 | 0.061 | 11.668 |
| 250 | 64160 | 60 | 30 | 44 | 5.2 | 0.4 | 3.03 | 15.27 | 80.15 | 0.061 | 11.668 |
| 300 | 73640 | 60 | 30 | 44 | 5.2 | 0.4 | 3.03 | 15.27 | 80.15 | 0.061 | 11.668 |
| 350 | 82730 | 60 | 30 | 44 | 5.2 | 0.4 | 3.03 | 15.27 | 80.15 | 0.061 | 11.668 |
| 380 | 92000 | 60 | 30 | 44 | 5.2 | 0.4 | 3.03 | 15.27 | 80.15 | 0.061 | 11.668 |
| 1 | 320 | 60 | 10 | 65 | 4 | 0.1 | 6.27 | 16.9 | 62.88 | 0.06 | 8.77 |
| 2 | 460 | 60 | 10 | 65 | 4 | 0.1 | 6.27 | 16.9 | 62.88 | 0.06 | 8.77 |
| 10 | 1100 | 60 | 10 | 65 | 4 | 0.1 | 6.27 | 16.9 | 62.88 | 0.06 | 8.77 |
| 100 | 3740 | 60 | 10 | 65 | 4 | 0.1 | 6.27 | 16.9 | 62.88 | 0.06 | 8.77 |
| 500 | 8830 | 60 | 10 | 65 | 4 | 0.1 | 6.27 | 16.9 | 62.88 | 0.06 | 8.77 |
| 1000 | 12790 | 60 | 10 | 65 | 4 | 0.1 | 6.27 | 16.9 | 62.88 | 0.06 | 8.77 |
| 2000 | 18510 | 60 | 10 | 65 | 4 | 0.1 | 6.27 | 16.9 | 62.88 | 0.06 | 8.77 |
| 3000 | 22000 | 60 | 10 | 65 | 4 | 0.1 | 6.27 | 16.9 | 62.88 | 0.06 | 8.77 |
| 4000 | 26810 | 60 | 10 | 65 | 4 | 0.1 | 6.27 | 16.9 | 62.88 | 0.06 | 8.77 |
| 4250 | 27980 | 60 | 10 | 65 | 4 | 0.1 | 6.27 | 16.9 | 62.88 | 0.06 | 8.77 |
| 1 | 190 | 60 | 10 | 65 | 4.6 | 0.1 | 4.18 | 15.89 | 73.69 | 0.06 | 10.298 |
| 2 | 280 | 60 | 10 | 65 | 4.6 | 0.1 | 4.18 | 15.89 | 73.69 | 0.06 | 10.298 |
| 10 | 710 | 60 | 10 | 65 | 4.6 | 0.1 | 4.18 | 15.89 | 73.69 | 0.06 | 10.298 |
| 100 | 2670 | 60 | 10 | 65 | 4.6 | 0.1 | 4.18 | 15.89 | 73.69 | 0.06 | 10.298 |
| 500 | 6750 | 60 | 10 | 65 | 4.6 | 0.1 | 4.18 | 15.89 | 73.69 | 0.06 | 10.298 |
| 1000 | 10050 | 60 | 10 | 65 | 4.6 | 0.1 | 4.18 | 15.89 | 73.69 | 0.06 | 10.298 |
| 2000 | 14960 | 60 | 10 | 65 | 4.6 | 0.1 | 4.18 | 15.89 | 73.69 | 0.06 | 10.298 |
| 3000 | 18890 | 60 | 10 | 65 | 4.6 | 0.1 | 4.18 | 15.89 | 73.69 | 0.06 | 10.298 |
| 4000 | 22600 | 60 | 10 | 65 | 4.6 | 0.1 | 4.18 | 15.89 | 73.69 | 0.06 | 10.298 |
| 1 | 130 | 60 | 10 | 65 | 5.2 | 0.1 | 3.47 | 16.11 | 78.44 | 0.06 | 11.701 |
| 2 | 200 | 60 | 10 | 65 | 5.2 | 0.1 | 3.47 | 16.11 | 78.44 | 0.06 | 11.701 |
| 10 | 520 | 60 | 10 | 65 | 5.2 | 0.1 | 3.47 | 16.11 | 78.44 | 0.06 | 11.701 |
| 100 | 2080 | 60 | 10 | 65 | 5.2 | 0.1 | 3.47 | 16.11 | 78.44 | 0.06 | 11.701 |
| 500 | 5480 | 60 | 10 | 65 | 5.2 | 0.1 | 3.47 | 16.11 | 78.44 | 0.06 | 11.701 |
| 1000 | 8310 | 60 | 10 | 65 | 5.2 | 0.1 | 3.47 | 16.11 | 78.44 | 0.06 | 11.701 |
| 2000 | 12620 | 60 | 10 | 65 | 5.2 | 0.1 | 3.47 | 16.11 | 78.44 | 0.06 | 11.701 |
| 3000 | 16110 | 60 | 10 | 65 | 5.2 | 0.1 | 3.47 | 16.11 | 78.44 | 0.06 | 11.701 |
| 3500 | 17680 | 60 | 10 | 65 | 5.2 | 0.1 | 3.47 | 16.11 | 78.44 | 0.06 | 11.701 |
| 3750 | 19000 | 60 | 10 | 65 | 5.2 | 0.1 | 3.47 | 16.11 | 78.44 | 0.06 | 11.701 |
| 1 | 500 | 60 | 20 | 65 | 4 | 0.1 | 6.67 | 17.25 | 61.33 | 0.06 | 8.737 |
| 2 | 730 | 60 | 20 | 65 | 4 | 0.1 | 6.67 | 17.25 | 61.33 | 0.06 | 8.737 |
| 10 | 1750 | 60 | 20 | 65 | 4 | 0.1 | 6.67 | 17.25 | 61.33 | 0.06 | 8.737 |
| 100 | 6120 | 60 | 20 | 65 | 4 | 0.1 | 6.67 | 17.25 | 61.33 | 0.06 | 8.737 |
| 500 | 14690 | 60 | 20 | 65 | 4 | 0.1 | 6.67 | 17.25 | 61.33 | 0.06 | 8.737 |
| 1000 | 21420 | 60 | 20 | 65 | 4 | 0.1 | 6.67 | 17.25 | 61.33 | 0.06 | 8.737 |
| 1250 | 24190 | 60 | 20 | 65 | 4 | 0.1 | 6.67 | 17.25 | 61.33 | 0.06 | 8.737 |
| 1350 | 25230 | 60 | 20 | 65 | 4 | 0.1 | 6.67 | 17.25 | 61.33 | 0.06 | 8.737 |
| 1400 | 25730 | 60 | 20 | 65 | 4 | 0.1 | 6.67 | 17.25 | 61.33 | 0.06 | 8.737 |
| 1420 | 26540 | 60 | 20 | 65 | 4 | 0.1 | 6.67 | 17.25 | 61.33 | 0.06 | 8.737 |
| 1 | 620 | 60 | 20 | 65 | 4.6 | 0.1 | 4.57 | 16.23 | 71.84 | 0.06 | 10.256 |
| 2 | 950 | 60 | 20 | 65 | 4.6 | 0.1 | 4.57 | 16.23 | 71.84 | 0.06 | 10.256 |
| **N** | **E_p_** | **T** | **S** | **P** | **C** | **D** | **A** | **M** | **F** | **B** | **E** |
| 10 | 2560 | 60 | 20 | 65 | 4.6 | 0.1 | 4.57 | 16.23 | 71.84 | 0.06 | 10.256 |
| 100 | 10560 | 60 | 20 | 65 | 4.6 | 0.1 | 4.57 | 16.23 | 71.84 | 0.06 | 10.256 |
| 500 | 28430 | 60 | 20 | 65 | 4.6 | 0.1 | 4.57 | 16.23 | 71.84 | 0.06 | 10.256 |
| 1000 | 43570 | 60 | 20 | 65 | 4.6 | 0.1 | 4.57 | 16.23 | 71.84 | 0.06 | 10.256 |
| 1250 | 49980 | 60 | 20 | 65 | 4.6 | 0.1 | 4.57 | 16.23 | 71.84 | 0.06 | 10.256 |
| 1500 | 56840 | 60 | 20 | 65 | 4.6 | 0.1 | 4.57 | 16.23 | 71.84 | 0.06 | 10.256 |
| 1 | 430 | 60 | 20 | 65 | 5.2 | 0.1 | 2.97 | 15.67 | 81.05 | 0.06 | 11.762 |
| 2 | 670 | 60 | 20 | 65 | 5.2 | 0.1 | 2.97 | 15.67 | 81.05 | 0.06 | 11.762 |
| 10 | 1890 | 60 | 20 | 65 | 5.2 | 0.1 | 2.97 | 15.67 | 81.05 | 0.06 | 11.762 |
| 100 | 8320 | 60 | 20 | 65 | 5.2 | 0.1 | 2.97 | 15.67 | 81.05 | 0.06 | 11.762 |
| 500 | 23440 | 60 | 20 | 65 | 5.2 | 0.1 | 2.97 | 15.67 | 81.05 | 0.06 | 11.762 |
| 1000 | 36610 | 60 | 20 | 65 | 5.2 | 0.1 | 2.97 | 15.67 | 81.05 | 0.06 | 11.762 |
| 1250 | 42270 | 60 | 20 | 65 | 5.2 | 0.1 | 2.97 | 15.67 | 81.05 | 0.06 | 11.762 |
| 1350 | 45420 | 60 | 20 | 65 | 5.2 | 0.1 | 2.97 | 15.67 | 81.05 | 0.06 | 11.762 |
| 1 | 780 | 60 | 30 | 65 | 4 | 0.1 | 6.69 | 17.27 | 61.25 | 0.06 | 8.735 |
| 2 | 1190 | 60 | 30 | 65 | 4 | 0.1 | 6.69 | 17.27 | 61.25 | 0.06 | 8.735 |
| 10 | 3190 | 60 | 30 | 65 | 4 | 0.1 | 6.69 | 17.27 | 61.25 | 0.06 | 8.735 |
| 100 | 13060 | 60 | 30 | 65 | 4 | 0.1 | 6.69 | 17.27 | 61.25 | 0.06 | 8.735 |
| 500 | 34960 | 60 | 30 | 65 | 4 | 0.1 | 6.69 | 17.27 | 61.25 | 0.06 | 8.735 |
| 535 | 36440 | 60 | 30 | 65 | 4 | 0.1 | 6.69 | 17.27 | 61.25 | 0.06 | 8.735 |
| 552 | 38600 | 60 | 30 | 65 | 4 | 0.1 | 6.69 | 17.27 | 61.25 | 0.06 | 8.735 |
| 1 | 750 | 60 | 30 | 65 | 4 | 0.1 | 6.69 | 17.27 | 61.25 | 0.06 | 8.735 |
| 2 | 1250 | 60 | 30 | 65 | 4 | 0.1 | 6.69 | 17.27 | 61.25 | 0.06 | 8.735 |
| 10 | 4050 | 60 | 30 | 65 | 4 | 0.1 | 6.69 | 17.27 | 61.25 | 0.06 | 8.735 |
| 100 | 21870 | 60 | 30 | 65 | 4 | 0.1 | 6.69 | 17.27 | 61.25 | 0.06 | 8.735 |
| 500 | 71080 | 60 | 30 | 65 | 4 | 0.1 | 6.69 | 17.27 | 61.25 | 0.06 | 8.735 |
| 600 | 81240 | 60 | 30 | 65 | 4 | 0.1 | 6.69 | 17.27 | 61.25 | 0.06 | 8.735 |
| 625 | 87200 | 60 | 30 | 65 | 4 | 0.1 | 6.69 | 17.27 | 61.25 | 0.06 | 8.735 |
| 1 | 730 | 60 | 30 | 65 | 5.2 | 0.1 | 3.1 | 15.78 | 80.37 | 0.06 | 11.747 |
| 2 | 1230 | 60 | 30 | 65 | 5.2 | 0.1 | 3.1 | 15.78 | 80.37 | 0.06 | 11.747 |
| 10 | 4120 | 60 | 30 | 65 | 5.2 | 0.1 | 3.1 | 15.78 | 80.37 | 0.06 | 11.747 |
| 100 | 23330 | 60 | 30 | 65 | 5.2 | 0.1 | 3.1 | 15.78 | 80.37 | 0.06 | 11.747 |
| 500 | 78300 | 60 | 30 | 65 | 5.2 | 0.1 | 3.1 | 15.78 | 80.37 | 0.06 | 11.747 |
| 600 | 89810 | 60 | 30 | 65 | 5.2 | 0.1 | 3.1 | 15.78 | 80.37 | 0.06 | 11.747 |
| 630 | 93160 | 60 | 30 | 65 | 5.2 | 0.1 | 3.1 | 15.78 | 80.37 | 0.06 | 11.747 |
| 650 | 95380 | 60 | 30 | 65 | 5.2 | 0.1 | 3.1 | 15.78 | 80.37 | 0.06 | 11.747 |
| 1 | 450 | 60 | 10 | 65 | 4 | 0.4 | 6.02 | 16.67 | 63.9 | 0.06 | 8.798 |
| 2 | 690 | 60 | 10 | 65 | 4 | 0.4 | 6.02 | 16.67 | 63.9 | 0.06 | 8.798 |
| 10 | 1870 | 60 | 10 | 65 | 4 | 0.4 | 6.02 | 16.67 | 63.9 | 0.06 | 8.798 |
| 100 | 7780 | 60 | 10 | 65 | 4 | 0.4 | 6.02 | 16.67 | 63.9 | 0.06 | 8.798 |
| 500 | 21090 | 60 | 10 | 65 | 4 | 0.4 | 6.02 | 16.67 | 63.9 | 0.06 | 8.798 |
| 1000 | 32390 | 60 | 10 | 65 | 4 | 0.4 | 6.02 | 16.67 | 63.9 | 0.06 | 8.798 |
| 1500 | 41640 | 60 | 10 | 65 | 4 | 0.4 | 6.02 | 16.67 | 63.9 | 0.06 | 8.798 |
| 1600 | 43340 | 60 | 10 | 65 | 4 | 0.4 | 6.02 | 16.67 | 63.9 | 0.06 | 8.798 |
| 1700 | 44990 | 60 | 10 | 65 | 4 | 0.4 | 6.02 | 16.67 | 63.9 | 0.06 | 8.798 |
| 1800 | 48000 | 60 | 10 | 65 | 4 | 0.4 | 6.02 | 16.67 | 63.9 | 0.06 | 8.798 |
| 1900 | 52800 | 60 | 10 | 65 | 4 | 0.4 | 6.02 | 16.67 | 63.9 | 0.06 | 8.798 |
| 1 | 265 | 60 | 10 | 65 | 4.6 | 0.4 | 4.08 | 15.79 | 74.2 | 0.06 | 10.309 |
| 2 | 420 | 60 | 10 | 65 | 4.6 | 0.4 | 4.08 | 15.79 | 74.2 | 0.06 | 10.309 |
| 10 | 1190 | 60 | 10 | 65 | 4.6 | 0.4 | 4.08 | 15.79 | 74.2 | 0.06 | 10.309 |
| 100 | 5390 | 60 | 10 | 65 | 4.6 | 0.4 | 4.08 | 15.79 | 74.2 | 0.06 | 10.309 |
| 500 | 15460 | 60 | 10 | 65 | 4.6 | 0.4 | 4.08 | 15.79 | 74.2 | 0.06 | 10.309 |
| **N** | **E_p_** | **T** | **S** | **P** | **C** | **D** | **A** | **M** | **F** | **B** | **E** |
| 1000 | 24330 | 60 | 10 | 65 | 4.6 | 0.4 | 4.08 | 15.79 | 74.2 | 0.06 | 10.309 |
| 1500 | 31720 | 60 | 10 | 65 | 4.6 | 0.4 | 4.08 | 15.79 | 74.2 | 0.06 | 10.309 |
| 1600 | 33090 | 60 | 10 | 65 | 4.6 | 0.4 | 4.08 | 15.79 | 74.2 | 0.06 | 10.309 |
| 1700 | 34430 | 60 | 10 | 65 | 4.6 | 0.4 | 4.08 | 15.79 | 74.2 | 0.06 | 10.309 |
| 1800 | 37800 | 60 | 10 | 65 | 4.6 | 0.4 | 4.08 | 15.79 | 74.2 | 0.06 | 10.309 |
| 1 | 180 | 60 | 10 | 65 | 5.2 | 0.4 | 2.54 | 15.29 | 83.43 | 0.06 | 11.815 |
| 2 | 290 | 60 | 10 | 65 | 5.2 | 0.4 | 2.54 | 15.29 | 83.43 | 0.06 | 11.815 |
| 10 | 830 | 60 | 10 | 65 | 5.2 | 0.4 | 2.54 | 15.29 | 83.43 | 0.06 | 11.815 |
| 100 | 3830 | 60 | 10 | 65 | 5.2 | 0.4 | 2.54 | 15.29 | 83.43 | 0.06 | 11.815 |
| 500 | 11160 | 60 | 10 | 65 | 5.2 | 0.4 | 2.54 | 15.29 | 83.43 | 0.06 | 11.815 |
| 1000 | 17700 | 60 | 10 | 65 | 5.2 | 0.4 | 2.54 | 15.29 | 83.43 | 0.06 | 11.815 |
| 1500 | 23160 | 60 | 10 | 65 | 5.2 | 0.4 | 2.54 | 15.29 | 83.43 | 0.06 | 11.815 |
| 1600 | 24180 | 60 | 10 | 65 | 5.2 | 0.4 | 2.54 | 15.29 | 83.43 | 0.06 | 11.815 |
| 1714 | 25170 | 60 | 10 | 65 | 5.2 | 0.4 | 2.54 | 15.29 | 83.43 | 0.06 | 11.815 |
| 1800 | 27300 | 60 | 10 | 65 | 5.2 | 0.4 | 2.54 | 15.29 | 83.43 | 0.06 | 11.815 |
| 1 | 515 | 60 | 20 | 65 | 4 | 0.4 | 6.68 | 17.26 | 61.3 | 0.06 | 8.736 |
| 2 | 790 | 60 | 20 | 65 | 4 | 0.4 | 6.68 | 17.26 | 61.3 | 0.06 | 8.736 |
| 10 | 2150 | 60 | 20 | 65 | 4 | 0.4 | 6.68 | 17.26 | 61.3 | 0.06 | 8.736 |
| 100 | 8940 | 60 | 20 | 65 | 4 | 0.4 | 6.68 | 17.26 | 61.3 | 0.06 | 8.736 |
| 500 | 24260 | 60 | 20 | 65 | 4 | 0.4 | 6.68 | 17.26 | 61.3 | 0.06 | 8.736 |
| 525 | 25000 | 60 | 20 | 65 | 4 | 0.4 | 6.68 | 17.26 | 61.3 | 0.06 | 8.736 |
| 550 | 25740 | 60 | 20 | 65 | 4 | 0.4 | 6.68 | 17.26 | 61.3 | 0.06 | 8.736 |
| 600 | 26500 | 60 | 20 | 65 | 4 | 0.4 | 6.68 | 17.26 | 61.3 | 0.06 | 8.736 |
| 650 | 28550 | 60 | 20 | 65 | 4 | 0.4 | 6.68 | 17.26 | 61.3 | 0.06 | 8.736 |
| 1 | 650 | 60 | 20 | 65 | 4.6 | 0.4 | 4.41 | 16.08 | 72.6 | 0.06 | 10.273 |
| 2 | 1050 | 60 | 20 | 65 | 4.6 | 0.4 | 4.41 | 16.08 | 72.6 | 0.06 | 10.273 |
| 10 | 3270 | 60 | 20 | 65 | 4.6 | 0.4 | 4.41 | 16.08 | 72.6 | 0.06 | 10.273 |
| 100 | 16480 | 60 | 20 | 65 | 4.6 | 0.4 | 4.41 | 16.08 | 72.6 | 0.06 | 10.273 |
| 500 | 51030 | 60 | 20 | 65 | 4.6 | 0.4 | 4.41 | 16.08 | 72.6 | 0.06 | 10.273 |
| 525 | 52810 | 60 | 20 | 65 | 4.6 | 0.4 | 4.41 | 16.08 | 72.6 | 0.06 | 10.273 |
| 550 | 54560 | 60 | 20 | 65 | 4.6 | 0.4 | 4.41 | 16.08 | 72.6 | 0.06 | 10.273 |
| 600 | 57100 | 60 | 20 | 65 | 4.6 | 0.4 | 4.41 | 16.08 | 72.6 | 0.06 | 10.273 |
| 650 | 64000 | 60 | 20 | 65 | 4.6 | 0.4 | 4.41 | 16.08 | 72.6 | 0.06 | 10.273 |
| 1 | 485 | 60 | 20 | 65 | 5.2 | 0.4 | 2.64 | 15.39 | 82.84 | 0.06 | 11.802 |
| 2 | 800 | 60 | 20 | 65 | 5.2 | 0.4 | 2.64 | 15.39 | 82.84 | 0.06 | 11.802 |
| 10 | 2580 | 60 | 20 | 65 | 5.2 | 0.4 | 2.64 | 15.39 | 82.84 | 0.06 | 11.802 |
| 100 | 13680 | 60 | 20 | 65 | 5.2 | 0.4 | 2.64 | 15.39 | 82.84 | 0.06 | 11.802 |
| 500 | 43960 | 60 | 20 | 65 | 5.2 | 0.4 | 2.64 | 15.39 | 82.84 | 0.06 | 11.802 |
| 525 | 45540 | 60 | 20 | 65 | 5.2 | 0.4 | 2.64 | 15.39 | 82.84 | 0.06 | 11.802 |
| 550 | 47100 | 60 | 20 | 65 | 5.2 | 0.4 | 2.64 | 15.39 | 82.84 | 0.06 | 11.802 |
| 600 | 50170 | 60 | 20 | 65 | 5.2 | 0.4 | 2.64 | 15.39 | 82.84 | 0.06 | 11.802 |
| 620 | 53100 | 60 | 20 | 65 | 5.2 | 0.4 | 2.64 | 15.39 | 82.84 | 0.06 | 11.802 |
| 1 | 1150 | 60 | 30 | 65 | 4 | 0.4 | 6.49 | 17.09 | 62.03 | 0.06 | 8.754 |
| 2 | 1930 | 60 | 30 | 65 | 4 | 0.4 | 6.49 | 17.09 | 62.03 | 0.06 | 8.754 |
| 10 | 6440 | 60 | 30 | 65 | 4 | 0.4 | 6.49 | 17.09 | 62.03 | 0.06 | 8.754 |
| 100 | 36110 | 60 | 30 | 65 | 4 | 0.4 | 6.49 | 17.09 | 62.03 | 0.06 | 8.754 |
| 150 | 48920 | 60 | 30 | 65 | 4 | 0.4 | 6.49 | 17.09 | 62.03 | 0.06 | 8.754 |
| 200 | 60670 | 60 | 30 | 65 | 4 | 0.4 | 6.49 | 17.09 | 62.03 | 0.06 | 8.754 |
| 250 | 71700 | 60 | 30 | 65 | 4 | 0.4 | 6.49 | 17.09 | 62.03 | 0.06 | 8.754 |
| 300 | 80000 | 60 | 30 | 65 | 4 | 0.4 | 6.49 | 17.09 | 62.03 | 0.06 | 8.754 |
| 350 | 89200 | 60 | 30 | 65 | 4 | 0.4 | 6.49 | 17.09 | 62.03 | 0.06 | 8.754 |
| 390 | 103000 | 60 | 30 | 65 | 4 | 0.4 | 6.49 | 17.09 | 62.03 | 0.06 | 8.754 |
| **N** | **E_p_** | **T** | **S** | **P** | **C** | **D** | **A** | **M** | **F** | **B** | **E** |
| 1 | 1080 | 60 | 30 | 65 | 4.6 | 0.4 | 4.1 | 15.81 | 74.1 | 0.06 | 10.307 |
| 2 | 1860 | 60 | 30 | 65 | 4.6 | 0.4 | 4.1 | 15.81 | 74.1 | 0.06 | 10.307 |
| 10 | 6570 | 60 | 30 | 65 | 4.6 | 0.4 | 4.1 | 15.81 | 74.1 | 0.06 | 10.307 |
| 100 | 39960 | 60 | 30 | 65 | 4.6 | 0.4 | 4.1 | 15.81 | 74.1 | 0.06 | 10.307 |
| 150 | 54910 | 60 | 30 | 65 | 4.6 | 0.4 | 4.1 | 15.81 | 74.1 | 0.06 | 10.307 |
| 200 | 68810 | 60 | 30 | 65 | 4.6 | 0.4 | 4.1 | 15.81 | 74.1 | 0.06 | 10.307 |
| 250 | 81970 | 60 | 30 | 65 | 4.6 | 0.4 | 4.1 | 15.81 | 74.1 | 0.06 | 10.307 |
| 300 | 91000 | 60 | 30 | 65 | 4.6 | 0.4 | 4.1 | 15.81 | 74.1 | 0.06 | 10.307 |
| 350 | 99100 | 60 | 30 | 65 | 4.6 | 0.4 | 4.1 | 15.81 | 74.1 | 0.06 | 10.307 |
| 1 | 1050 | 60 | 30 | 65 | 5.2 | 0.4 | 2.97 | 15.67 | 81.05 | 0.06 | 11.762 |
| 2 | 1780 | 60 | 30 | 65 | 5.2 | 0.4 | 2.97 | 15.67 | 81.05 | 0.06 | 11.762 |
| 10 | 6160 | 60 | 30 | 65 | 5.2 | 0.4 | 2.97 | 15.67 | 81.05 | 0.06 | 11.762 |
| 100 | 36140 | 60 | 30 | 65 | 5.2 | 0.4 | 2.97 | 15.67 | 81.05 | 0.06 | 11.762 |
| 150 | 49350 | 60 | 30 | 65 | 5.2 | 0.4 | 2.97 | 15.67 | 81.05 | 0.06 | 11.762 |
| 200 | 61560 | 60 | 30 | 65 | 5.2 | 0.4 | 2.97 | 15.67 | 81.05 | 0.06 | 11.762 |
| 250 | 73070 | 60 | 30 | 65 | 5.2 | 0.4 | 2.97 | 15.67 | 81.05 | 0.06 | 11.762 |
| 300 | 84060 | 60 | 30 | 65 | 5.2 | 0.4 | 2.97 | 15.67 | 81.05 | 0.06 | 11.762 |
| 350 | 99300 | 60 | 30 | 65 | 5.2 | 0.4 | 2.97 | 15.67 | 81.05 | 0.06 | 11.762 |
